# Sustainability in Health care by Allocating Resources Effectively (SHARE) 11: reporting outcomes of an evidence-driven approach to disinvestment in a local healthcare setting

**DOI:** 10.1186/s12913-018-3172-0

**Published:** 2018-05-30

**Authors:** Claire Harris, Kelly Allen, Wayne Ramsey, Richard King, Sally Green

**Affiliations:** 1 School of Public Health and Preventive Medicine, Monash University, Melbourne, VIC Australia; 2 Centre for Clinical Effectiveness, Monash Health, Melbourne, VIC Australia; 3 Medical Services and Quality, Monash Health, Melbourne, VIC Australia; 4 Medicine Program, Monash Health, Melbourne, VIC Australia

**Keywords:** Disinvestment, Decommission, de-adopt, de-list, de-implement, Health technology, TCP, Resource allocation, Decision-making, Implementation

## Abstract

**Background:**

This is the final paper in a thematic series reporting a program of Sustainability in Health care by Allocating Resources Effectively (SHARE) in a local healthcare setting. The SHARE Program was established to explore a systematic, integrated, evidence-based organisation-wide approach to disinvestment in a large Australian health service network. This paper summarises the findings, discusses the contribution of the SHARE Program to the body of knowledge and understanding of disinvestment in the local healthcare setting, and considers implications for policy, practice and research.

**Discussion:**

The SHARE program was conducted in three phases. Phase One was undertaken to understand concepts and practices related to disinvestment and the implications for a local health service and, based on this information, to identify potential settings and methods for decision-making about disinvestment. The aim of Phase Two was to implement and evaluate the proposed methods to determine which were sustainable, effective and appropriate in a local health service. A review of the current literature incorporating the SHARE findings was conducted in Phase Three to contribute to the understanding of systematic approaches to disinvestment in the local healthcare context.

SHARE differed from many other published examples of disinvestment in several ways: by seeking to identify and implement disinvestment opportunities within organisational infrastructure rather than as standalone projects; considering disinvestment in the context of all resource allocation decisions rather than in isolation; including allocation of non-monetary resources as well as financial decisions; and focusing on effective use of limited resources to optimise healthcare outcomes.

**Conclusion:**

The SHARE findings provide a rich source of new information about local health service decision-making, in a level of detail not previously reported, to inform others in similar situations. Multiple innovations related to disinvestment were found to be acceptable and feasible in the local setting. Factors influencing decision-making, implementation processes and final outcomes were identified; and methods for further exploration, or avoidance, in attempting disinvestment in this context are proposed based on these findings. The settings, frameworks, models, methods and tools arising from the SHARE findings have potential to enhance health care and patient outcomes.

**Electronic supplementary material:**

The online version of this article (10.1186/s12913-018-3172-0) contains supplementary material, which is available to authorized users.

## About SHARE

*This is the eleventh in a series of papers reporting Sustainability in Health care by Allocating Resources Effectively (SHARE). The SHARE Program is an investigation of concepts, opportunities, methods and implications for evidence-based investment and disinvestment in health technologies and clinical practices in a local healthcare setting. The papers in this series are targeted at clinicians, managers, policy makers, health service researchers and implementation scientists working in this context. This paper presents the findings and key messages from investigation of an organisation-wide, systematic, integrated, evidence-based approach to disinvestment taken by one Australian healthcare network.*

## Background

The concept of disinvestment has emerged in response to rising healthcare costs, continuing advances in expensive health technologies and increasing recognition of ineffective practices and systemic waste in health services [1–7]. There are three main areas of opportunity for removal, reduction or restriction of health technologies and clinical practices (TCPs): 1) TCPs in current use that were not evaluated rigorously prior to their introduction and have subsequently been identified as unsafe, ineffective or not cost-effective; 2) TCPs that are safe, effective and cost-effective but which have alternatives offering greater benefit; and 3) TCPs that are overused or misused [8].

Following successful implementation of a rigorous evidence-based program for introduction of new TCPs [9], members of the Technology/Clinical Practice Committee at Monash Health, a large health service network in Melbourne, Australia, sought to implement a similar program for disinvestment. The ‘Sustainability in Health care by Allocating Resources Effectively’ (SHARE) Program was established in 2009 to investigate a systematic, integrated, evidence-based approach to disinvestment in the context of organisation-wide systems and processes.

Research and debate in disinvestment have broadened considerably over the past decade, yet a number of significant gaps remain. There is little evidence to guide local healthcare facilities in how they might take a systematic organisation-wide approach [10–19]. There is also a lack of information about the factors that influence resource allocation, the processes involved in implementation of disinvestment decisions, and the perspectives and experiences of healthcare staff undertaking disinvestment [10, 19–22].

**Fig. 1:**
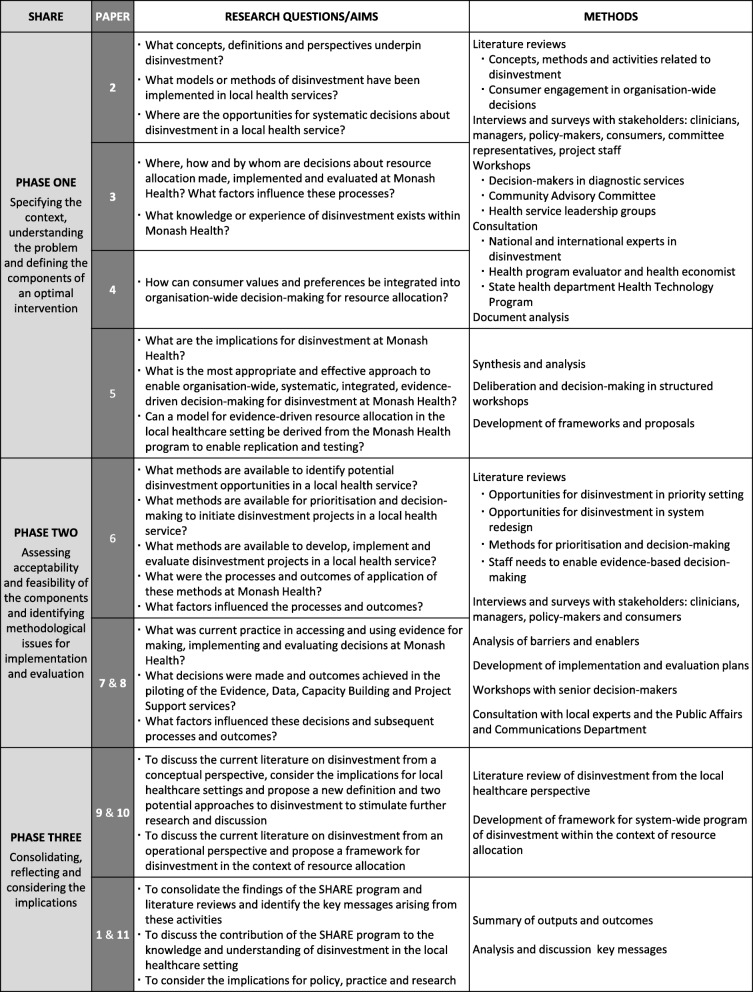
Overview of the SHARE Program

In the absence of guidance from the literature, a two-phased process was implemented to identify and then evaluate potential opportunities for disinvestment at Monash Health (Fig. 1). These investigations are presented using a case study approach to describe, explore and explain the decisions, processes and outcomes to address some of the gaps in knowledge and facilitate development of theory and interventions [23–29]. A review and synthesis of the disinvestment literature incorporating the SHARE findings was undertaken as a third phase [8, 30].

Monash Health is a network of six acute hospitals, subacute and rehabilitation services, mental health and community health services, and residential aged care [31]. The SHARE Program was funded as a three-year demonstration project by the Victorian Government Department of Human Services (DHS) and was undertaken by the Centre for Clinical Effectiveness (CCE), an in-house resource at Monash Health aiming to facilitate evidence-based practice. The overall approach to SHARE program activities was underpinned by the UK Medical Research Council framework for design and evaluation of complex interventions [32] and the SEAchange model for Sustainable, Effective and Appropriate evidence-based change in health services [33]. To address the limited understanding of resource allocation processes in health services, and the lack of detail in reporting of implementation of change in the literature [34–36], the SHARE papers are presented using appropriate case study methods [37–40] and reporting guidelines [41–43].

An overview of the SHARE Program, guide to the SHARE publications and further details about Monash Health are provided in the first paper in this series [44].

## Aims

The aims of this paper are to 1) consolidate the SHARE findings, 2) discuss the contribution of the SHARE Program to the knowledge and understanding of disinvestment in the local healthcare setting, and 3) consider the implications for policy, practice and research.

## Findings of the SHARE Program

A complete summary of SHARE Papers 2–10, including tables of findings and all figures, are presented and discussed in the context of the current literature in Additional file 1. A brief overview is presented below.

### Phase One

Based on the UK Medical Research Council framework for complex interventions [32], Phase One involved specifying the context, understanding the problem and defining the components of an optimal intervention (Fig. 1).

#### Specifying the context

The activities focused on understanding disinvestment from the local health service perspective and identifying potential mechanisms for a systematic organisation-wide approach [23, 45]. No models, methods or practical advice regarding an organisation-wide approach to disinvestment were identified. Hence, a conceptual list of issues to consider was compiled and a framework of six potential mechanisms to systematically introduce disinvestment decisions within health service infrastructure was developed to provide direction for further investigation (Additional file 1: Table S1 and Figure S1) [23].

#### Understanding the problem

In order to introduce the proposed organisation-wide program for disinvestment, knowledge of existing decision-making systems and processes for investment within Monash Health was required. While there was a broad understanding of where resource allocation decisions were made, detailed knowledge of who made them and how they were made, implemented and evaluated was lacking, and this information was also unavailable in the literature [24]. This investigation identified, and enabled development of classifications for, groups and individuals authorised to make decisions on behalf of the organisation, decision-making settings, and type and scope of decisions (Additional file 1: Table S2). The findings also included recognition of eight components in the resource allocation process, the elements of structure and practice for each component and the relationships between them represented as a framework for resource allocation in the local setting. The eight components are Governance, Administration, Stakeholder engagement, Resources, Decision-making, Implementation, Evaluation and, where appropriate, Reinvestment of savings (Additional file 1: Figure S3 and Table S3). Strengths, weaknesses, barriers and enablers to the resource allocation process; examples of criteria used by different decision-making groups; the types and sources of data used in evaluation; and differences in the decision-making processes and information needs of medical, nursing, allied health and management/support groups were reported (Additional file 1: Tables S4-S7).

The term ‘disinvestment’ was generally unfamiliar to local decision-makers; but the concept was readily understood. At Monash Health, removal, reduction or restriction of current practices or reallocation of resources were initiated by quality and safety issues, evidence-based practice (EBP), or a need to find resource savings, and not by a primary aim ‘to disinvest’ [24].

Consumer engagement was integral to the proposed program; however there was a lack of guidance about systematic approaches to identify, capture and incorporate consumer perspectives into resource allocation decision-making, implementation and evaluation [25]. Findings from the literature and local research were used to develop a model to integrate consumer values and preferences into organisation-wide decision-making based on the framework for resource allocation noted above (Additional file 1: Figure S4 and Tables S8-S11) [25].

#### Defining the components

**Fig. 2:**
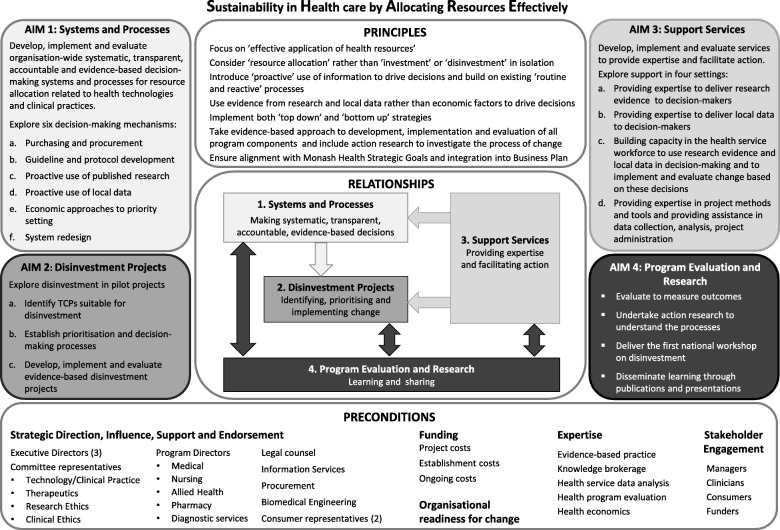
Model for exploring Sustainability in Health care by Allocating Resources Effectively in the local healthcare setting (Reproduced with permission from SHARE Paper 5 [26])

The findings of the investigations above (Fig. 1) were synthesised and analysed to identify the most sustainable, effective and appropriate approach to disinvestment at Monash Health [26]. Multiple factors for consideration in establishment of the new program were extracted (Additional file 1: Table S12). This led to definition of the program elements: four components, their aims and objectives, relationships between the components, principles that underpin the program and preconditions for success and sustainability. The principles were agreed upon, the preconditions were established, and implementation and evaluation plans were developed. The program elements were incorporated into a model for sustainability in health care by allocating resources effectively (SHARE) in the local healthcare setting (Fig. 2) [26].

### Phase Two

Phase Two involved a series of exploratory trials assessing acceptability and feasibility of the four components (Fig. 2) to determine which were effective, appropriate and sustainable at Monash Health and to identify methodological issues for implementation and evaluation [32].

Funding was reduced in the final year of the program resulting in limitation of some implementation and evaluation activities due to the shortened timelines.

**Fig. 3:**
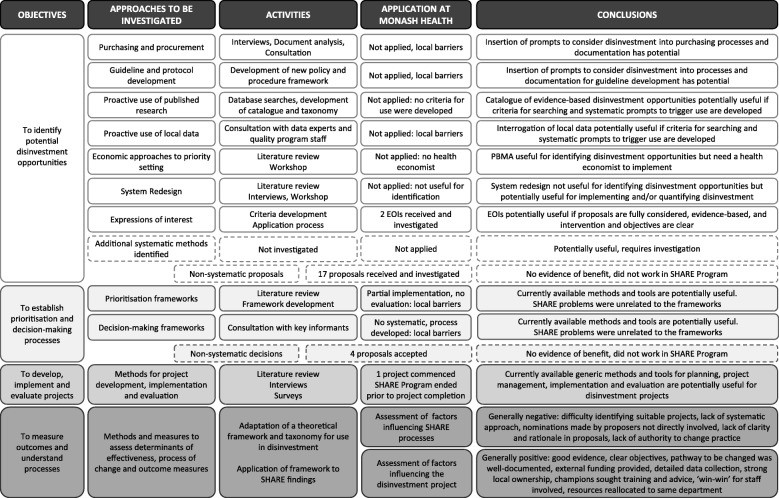
Overview of activities for SHARE Aims 1 and 2 (Reproduced with permission from SHARE Paper 6 [27])

Summaries of the activities in Aims 1 and 2 are provided in Fig. 3.

#### Aim 1. Systems and processes

The focus of Aim 1 was to explore the six proposed decision-making mechanisms with potential to systematically identify opportunities for disinvestment within organisational systems and processes [23].

##### Aim 1.1 Purchasing and procurement

Incorporating prompts, triggers and mandatory requirements to consider disinvestment within existing systems and processes for purchasing of drugs and clinical consumables and capital procurement of equipment was proposed [23]. The SHARE activities resulted in some positive outcomes related to introduction of new TCPs, but no changes regarding identification of opportunities for disinvestment were implemented [27]. This was due to local barriers; in particular that the relevant processes were outside the control of the SHARE team.

##### Aim 1.2 Guideline and protocol development

Similarly, prompts, triggers and mandatory requirements to consider disinvestment could be introduced into document development and authorisation processes, implementation and evaluation activities for local guidelines and protocols that determine use of drugs and equipment, diagnostic tests, surgical procedures, clinic capacity, etc. [23]. The SHARE team included a prompt in the instructions to document developers to consider whether any current practices could be discontinued in the new Monash Health Policy and Procedure Framework [46], however this was removed by the implementers (from another department with responsibility for governance of the new framework) who felt the process was too onerous [27].

##### Aim 1.3 Proactive use of published research

Proactive searches for evidence-based disinvestment opportunities from the research literature could be undertaken and the findings delivered directly to decision-makers [23]. The SHARE team developed a catalogue of potential disinvestment targets from known sources of high quality synthesised evidence [47–51] and evidence-based publications focused on disinvestment [52, 53]. Use of the catalogue to identify disinvestment projects is discussed in Aim 2.1 below [27]. A broader approach to proactive use of research evidence was piloted as an Evidence Dissemination Service which is discussed in Aim 3.1 below [29].

##### Aim 1.4 Proactive use of local data

Similarly, routinely-collected health service data could be searched proactively to identify areas where disinvestment might have the greatest impact such as high cost, high volume, high rates of adverse events, etc.; and to investigate variations in practice between campuses, departments or individuals within the health service, or with other equivalent institutions, to identify inappropriate or suboptimal practices [23]. These approaches were to be explored within the Data Service which is discussed in Aim 3.2 [28].

##### Aim 1.5 Economic approaches to priority setting

Priority setting exercises use economic principles to weigh up options for investment and disinvestment and select preferred alternatives using pre-determined criteria [23]. Four methods of priority setting met the criteria of economic analysis applicable at the local health service level; however all had limitations in their ability to identify disinvestment opportunities in this context [27]. The lack of in-house health economics capability was the key factor in the decision that economic approaches to priority setting were not feasible at Monash Health [27].

##### Aim 1.6 System redesign

System redesign describes a range of methods and tools to review whole systems of care. It is a familiar process in health services, it offers a well-accepted context to introduce practice change, and it could be integrated into a systematic organisation-wide approach to disinvestment [23]. No examples of system redesign specifically related to disinvestment were identified from the literature or by Monash Health respondents with expertise in this area [27]. The SHARE Steering Committee decided that system redesign methods would not be used to identify opportunities for disinvestment, but may be useful in implementing decisions to disinvest.

#### Aim 2. Disinvestment projects

Investigation of pilot disinvestment projects was proposed to understand the processes involved, assess the resources required, provide practical guidance for future projects and, if successful, be used as positive examples to promote subsequent disinvestment activities.

##### Aim 2.1 Identification of disinvestment opportunities

An ‘Expression of Interest’ (EOI) process where health service staff nominated their own disinvestment projects was added to the six methods to be investigated in Aim 1 [27].

Although an evidence-based catalogue of disinvestment opportunities had been developed, an ad hoc process whereby SHARE Steering Committee members submitted disinvestment proposals at meetings dominated the decision-making process and the catalogue was not used [27]. An algorithm for identifying disinvestment projects from the catalogue was developed (Additional file 1: Figure S7), however the planned development of transparent criteria to be used in its application was not undertaken [27]. Two EOIs and 17 ad hoc proposals were investigated as potential pilot disinvestment projects (Additional file 1: Table S14) [27].

##### Aim 2.2 Prioritisation and decision-making

A literature review found guidelines and systematic reviews for prioritisation of new and existing TCPs. These were adapted into a tool which was to be piloted in the annual capital expenditure funding round. The tool was not tested; the capital expenditure process was cancelled as Monash Health had no spare capital [27].

Prioritisation tools primarily focus on characteristics intrinsic to the TCP. However additional criteria may influence whether a TCP is selected for a local practice change initiative; for example likelihood of success or sustainability, availability of external funds, or value of the evaluation to other processes (Additional file 1: Tables S15 and S16). Due to the dominance of the ad hoc process, no explicit decision-making criteria were developed. Decisions were pragmatic, based on likelihood of ‘quick wins’ and other unspecified factors related to the proposed TCPs.

Of the 19 proposed TCPs, four were not investigated as subsequent proposals were thought to have greater potential; two had incomplete investigations for the same reason; nine were rejected for a range of issues; and four were accepted as pilot projects (Additional file 1: Table S14).

##### Aim 2.3 Development, implementation and evaluation of disinvestment projects

No published guidance for disinvestment projects in the local context was identified; however Monash Health staff provided details of strengths, weaknesses, barriers and enablers in these processes (Additional file 1: Table S4) [24] and needs for assistance to undertake projects [28]. Implementation and evaluation methods were planned for the SHARE disinvestment pilot projects, however only one reached the implementation stage and evaluation was limited due to the reduction of funding in the final year [27].

##### Influencing factors

Factors influencing the SHARE process for identification, prioritisation and decision-making, implementation and evaluation of potential projects and those influencing the single pilot project are outlined in Additional file 1: Tables S17 and S18.

#### Aim 3. Support services

**Fig. 4:**
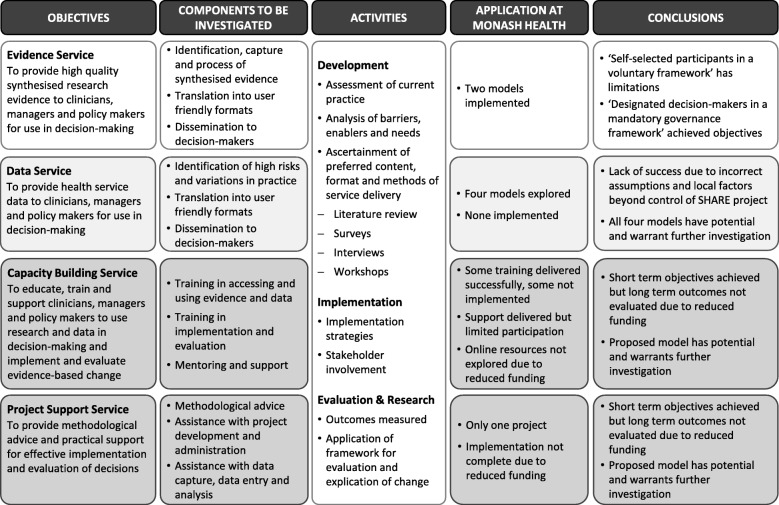
Overview of SHARE Aim 3 (Reproduced with permission from SHARE Paper 7 [28])

Local research confirmed the findings of other studies that evidence from research and local data is not used systematically or proactively to drive decisions; that health service personnel usually lack the time, knowledge, skills and resources to access and identify the information they require and appraise it for quality and relevance; that clinicians charged with undertaking projects commonly do not know how to implement and evaluate change or manage projects effectively; and that projects are generally under-resourced [28, 29]. Respondents were aware of their limitations and those of their colleagues in undertaking projects and they welcomed advice and support [28]. Four support services were proposed to address these barriers in Aim 3 (Fig. 2). An overview of the investigation is provided (Fig. 4) and summaries of factors that influenced development, processes and outcomes of the support services are found in Additional file 1: Tables S19 and S20.

##### Aim 3.1 Evidence Dissemination Service

The Evidence Dissemination Service (EDS) was conceived as a method of identifying disinvestment opportunities by delivering recently published, high quality, synthesised evidence directly to decision-makers [29]. It became clear during development that this could also be a way to ensure that all practice at Monash Health was consistent with current evidence. Two models were implemented (Additional file 1: Figure S9).

Model 1 sent weekly email ‘Evidence Alerts’ containing citations, hyperlinked to abstracts, hyperlinked to full text, to EDS subscribers. This model could not achieve its aims. The main factor was lack of governance; there was no process to ensure that the appropriate person with authority in the area affected by the evidence had considered the information, made a decision or taken any action. The second factor was lack of time to undertake the steps required in production and utilisation of the Evidence Alerts; this was reported by both the EDS team who captured, processed and disseminated the publications and the decision-makers who were required to appraise for quality and applicability and take appropriate action. In addition, many publications were already known to recipients, not relevant to their area of practice, not applicable at Monash Health, consistent with current practice, not important enough to instigate change, or reported lack of evidence; hence required no action. This resulted in time wasted by both the EDS team and the decision-makers.

Model 2 addressed these issues (Additional file 1: Figure S10). Publications were limited to those demonstrating evidence of harm, lack of effect, and availability of a cost-effective alternative, which were priorities of Monash Health at the time and consistent with the aim of identifying opportunities for disinvestment. The findings of these studies were compared with current documented practice in local policies and procedures. If there was no local documentation, or it was inconsistent with the evidence, the publication was appraised for quality and forwarded to the governing body, the Technology/Clinical Practice Committee, to assess local applicability and identify the relevant organisational decision-maker, usually a department head or committee chair. An ‘Evidence Bulletin’ which included information extracted from the publication, the quality appraisal findings and a reporting template was then sent to the relevant authorised decision-maker (Additional file 1: Figure S11). This became an organisational priority; when there was evidence of harm, responses were required within one month and were reported to the Chief Executive the following month at her request.

There are other services disseminating evidence to subscribers. The unique characteristics of the EDS are outlined in Additional file 1: Table S21).

While this was successful in aligning local practice with current evidence, it was a very resource-intensive process and CCE had insufficient staff capacity to maintain it while meeting other commitments. The EDS was suspended in the last few months of the SHARE Program, however it has subsequently been reinstated and is focused on the ‘Choosing Wisely’ literature [54].

##### Aim 3.2 Data Service

The Data Service was initiated to complement the EDS by delivering local data to decision-makers. Four models of a Data Service were explored, but none were implemented due to local factors such as limited staff capacity and problems with access and coordination of local data [28]. As a result, proactive use of health service data was not employed to identify disinvestment targets for pilot projects.

##### Aim 3.3 Capacity Building Service

The aim of this service was to train and support staff to use research evidence and local data in decision-making and then implement and evaluate these decisions in successful projects [28]. A summary of the education and support programs provided is included in Additional file 1: Table S22. Evaluation immediately after workshops showed participants’ knowledge and confidence improved in all aspects of the evidence-based change process and the concepts of EBP, implementation and evaluation. There were further improvements after three months, however there were only a small number of responses. Participants reported high rates of satisfaction and noted that the workshops met or exceeded their expectations [28]. Due to the reduced funding in the final year of the SHARE Program, the service was not expanded beyond the pilot.

##### Aim 3.4 Project Support Service

The Project Support Service was established to support the clinical staff undertaking SHARE disinvestment pilot projects [27]. It was anticipated that methodological advice and support would be delivered in a range of activities related to project planning, governance and administration; implementation and evaluation and practical assistance would be provided for data capture, entry and analysis (Additional file 1: Table S23). One of the four clinical teams required support in all of these areas. The other three were still in the decision-making and development phase and needed assistance in finding evidence and data, determining the nature and scope of the problem, clarifying the intervention and assessing feasibility and risk. These projects were subsequently withdrawn based on the outcomes of this process.

Each of the teams acknowledged their lack of skills and experience in using evidence in decision-making, project management, implementation and evaluation. They were appreciative that support was available and were willing to accept guidance.

#### Aim 4. Program evaluation and research

Although each of the first three aims included evaluation in their pilot and implementation phases, a fourth aim was specified to highlight the importance of evaluation, research and dissemination in capturing and understanding what happened and sharing this with others interested in developing similar models.

##### Aim 4.1 Evaluation and explication

An evaluation framework and plan was developed for the overall SHARE Program and included evaluation domains, audience, scope, evaluation questions, outcomes hierarchy, sources of data, methods of collection and analysis, reporting and timelines [55]. More detailed evaluation plans were developed for individual projects.

Factors that influenced development, processes and outcomes of individual projects were identified using four adaptations of an existing framework and taxonomy for evaluation and explication of evidence-based innovations [56] which were used in a range of applications in the SHARE Program (Additional file 1: Figure S12).

##### Aim 4.2 Action research

Action research was undertaken based on the “*researcher as facilitator for change*” model defined by Meyer [57, 58]. An agenda item for ‘Learnings’ was scheduled at the beginning of every team meeting. Participants were invited to consider anything that had affected the project since the last meeting using the framework ‘what worked, what didn’t, why and how it could be improved’. Each issue, its effect on the project, and potential changes that would build on positive outcomes or remove or minimise future problems were discussed. The learnings and actions were documented; actions were assigned, given timeframes and followed up. These methods worked well.

##### Aim 4.3 National workshop

The first Australian national workshop on disinvestment was conducted to share knowledge and develop links for future collaboration. Disinvestment was considered from three perspectives: health policy researchers, health economists and health service decision-makers. All findings and presentation materials were published [59, 60].

##### Aim 4.4 Dissemination

To address some of the gaps in knowledge and contribute to the understanding of systematic approaches to disinvestment and resource allocation in the local healthcare context, the SHARE Program activities are presented in this thematic series and a review of the current literature incorporating the SHARE findings was undertaken in Phase Three.

### Phase Three

The literature reviews are presented in two debate papers (Table 1). Paper 9 considers the conceptual elements of disinvestment from the perspective of local healthcare services and proposes a new definition and two potential approaches to disinvestment [8]. Paper 10 presents the operational elements in the context of a new framework for disinvestment in the local setting [30].

**Table 1:** Contents of the literature reviews (Reproduced with permission from SHARE Paper 9 [8])

Conceptual review (Paper 9)	Operational review (Paper 10)
▪ Terminology and concepts - Health technologies - Disinvestment - Resource allocation - Optimising health care - Reinvestment ▪ Motivation and purpose - Impetus for disinvestment - Rationale for disinvestment ▪ Relationships with other health paradigms - Evidence based health care - Quality improvement - System redesign - Health economic approaches ▪ Challenges ▪ New approach to disinvestment	▪ Existing theories, frameworks and models ▪ New framework - Audience - Application - Definitions - Concepts - Components ▪ Principles of decision-making ▪ Settings - Decision-making infrastructure - Specific initiatives - Individual decision-makers ▪ Prompts and triggers ▪ Steps in the disinvestment process ▪ Methods and tools ▪ Barriers and enablers

#### Terminology and concepts

There are multiple definitions for the terms ‘disinvestment’ and ‘health technology’, a lack of common understanding of the reasons or objectives that underpin the concepts, and disparity in use of the terms between the research and practice settings (Additional file 1: Tables S25 and S26). This creates difficulties in the interpretation of disinvestment, application of research findings, and establishment of a systematic approach in the local healthcare setting.

In the absence of common terminology, there is one notably consistent message: that the word ‘disinvestment’ has negative connotations and is likely to be a barrier to successful implementation of disinvestment-related change. To reduce undesirable effects, other terms have been intentionally introduced to replace ‘disinvestment’ (Additional file 1: Table S27) and other concepts such as ‘resource allocation’, ‘optimisation of healthcare’ and ‘safely doing less’ have been proposed as alternative approaches [8, 61].

#### Motivation and purpose

The reasons underpinning specific disinvestment activities are not widely discussed although many of the definitions include or imply a reason for disinvestment which can be summarised in seven main themes. An eighth option, ‘for any reason’, is added for completeness (Additional file 1: Table S28 and Figure S13). There are many more reasons for removing, reducing or restricting use of TCPs from the perspective of a local healthcare service than those captured in the definitions for disinvestment (Additional file 1: Table S29). Understanding the rationale for a disinvestment initiative is crucial to project planning as it is likely to affect all aspects of the process from identification and prioritisation through to implementation and evaluation.

#### Relationship with other healthcare improvement paradigms

Disinvestment is frequently portrayed as if it is a new paradigm for health improvement. It has been described as an ‘emerging field’. Disinvestment approaches, processes and initiatives are discussed; ‘research agendas’ are considered; and a need for mechanisms, frameworks, methods and tools is noted. Although there are existing health improvement paradigms that address disinvestment-type activities, these are not routinely promoted in implementation and evaluation of disinvestment. For example, EBP, quality improvement and system redesign all have mature frameworks with validated methods that are widely-used and well-accepted in local health services. It is not clear why there is a need for new methods specific to disinvestment in preference to building on existing familiar processes.

#### Challenges

The nature of disinvestment brings some particular challenges to achieving change. These include a sense of loss; challenges to professional expertise and autonomy; need for more convincing evidence; possibility of benefit in some cases; heterogeneity of outcomes; lack of data and formal methods for quantifying savings and benefits; lack of standardised methods for disinvestment decisions; lack of transparency in disinvestment processes; nomination of disinvestment targets by ‘outsiders’; lack of clarity and rationale and insufficient information to support disinvestment proposals; and difficulties for those who make decisions in multiple roles with potentially conflicting perspectives.

#### Redefining disinvestment

There is little evidence of active and successful implementation of specific ‘disinvestment initiatives’ in the local healthcare setting and specifically seeking out targets when the expressed aim is ‘to disinvest’ has not been effective. Yet successful removal, reduction, restriction and replacement of technologies, clinical practices, programs and services are commonplace at the health service level. This suggests that the construct of ‘disinvestment’ may be problematic in the local healthcare setting. To stimulate research and debate, we put forward two options that address some of the issues identified in Paper 9 [8].

The first proposed that if the concept of ‘disinvestment’ is to remain as a specific aim and activity, the terminology, research paradigm and methods of application must be clarified, consolidated and agreed upon.

The second proposed that the concept of disinvestment is simplified, so that it is not a specific aim or activity, and assimilated within familiar health improvement paradigms so that it builds on existing knowledge and expertise in the health workforce. The term ‘disinvestment’ would be used in the broadest sense, effectively the opposite of investment; as ‘removal, reduction or restriction of any aspect of the health system for any reason’. Unlike most of the research definitions for disinvestment, this version is not constrained by a specified purpose, defined criteria or anticipated outcomes. Disinvestment becomes the outcome of, rather than the reason for, a resource allocation decision. In contrast, we propose that ‘health technologies’ is defined in the narrowest sense; as products, devices and equipment used to deliver health care (eg prostheses, implantable devices, vaccines, pharmaceuticals, surgical instruments, telehealth, interactive IT and diagnostic tools) which reflects common use by health service staff and consumers.

#### Theories, frameworks and models

There is little discussion of the role of theory or theoretical approaches to disinvestment in the literature, however 15 frameworks and models related to disinvestment, resource allocation and priority setting were identified (Additional file 1: Table S30) [30].

#### New framework for an organisation-wide approach to disinvestment in the local healthcare setting

**Fig. 5:**
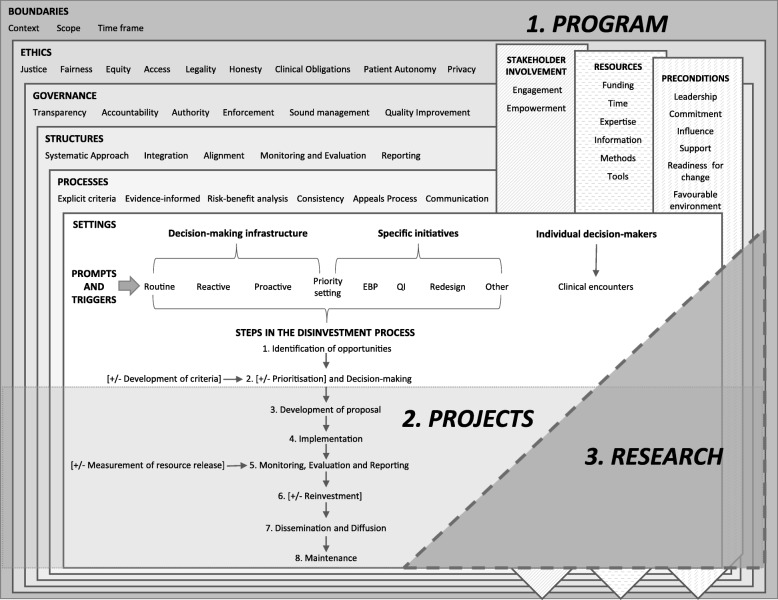
Framework for an organisation-wide approach to disinvestment in the local healthcare setting (Reproduced with permission from SHARE Paper 10 [30])

There is no overarching framework for disinvestment in this setting. However, there are clear and consistent messages in the literature which, along with the detailed findings from the SHARE projects, were used as the basis for a new framework for operationalising disinvestment (Fig. 5).

The framework is proposed as an organisation-wide application, embedded within existing systems and processes, which can be responsive to local needs and priorities, and employed in policy, management or clinical contexts.

It brings together the definitions, concepts, principles, decision-making settings, potential prompts and triggers to consider disinvestment, and steps in the disinvestment process found in the literature.

The framework is composed of three interconnected and interdependent components: 1) a program for organisation-wide decision-making, 2) projects to implement decisions and evaluate outcomes, and 3) research to understand and improve the program and project activities. The program consists of principles for decision-making and settings that provide opportunities to introduce systematic prompts and triggers to initiate disinvestment. The projects follow the steps in the disinvestment process. Each component has a number of elements which are outlined in detail in Paper 10 and summarised in Additional file 1: Tables S31-S35. There is potential for research in all elements of the program and projects.

Potential methods and tools are presented and discussed in Paper 10, however the framework does not stipulate project design or conduct; allowing application of any theories, methods or tools at each step. Barriers are discussed and examples illustrating constituent elements are provided (Additional file 1: Table S36).

## Strengths and limitations

The main strengths of the SHARE Program were the explicit evidence-based approach, adequate resources for most of the program, support at the highest levels, favourable timing, and strong, consistent messages from a diverse range of stakeholders.

Views of Monash Health staff and consumers were sought including executives, senior managers, clinical managers, clinicians, project staff with experience in disinvestment-type activities, and representatives of committees with responsibility for resource allocation decisions. Participants represented all clinical disciplines, all levels of seniority and all campuses.

Decisions were based on information from the research literature and local data, integrated with the views of experts in the field and local health service staff and consumers. This approach facilitates development of strategies that are more likely to be sustainable, effective and appropriate [21, 33]. Stakeholder feedback was sought during development, implementation and evaluation of interventions and revisions were made accordingly.

This rigorous approach was possible due to the provision of funding from the Victorian DHS and Monash Health. The SHARE team had appropriate skills for most of the activities and adequate time was allocated to undertake it; consultants were engaged to add specific expertise that was not available in-house. Loss of funding towards the end of the program is noted below as a limitation.

The 20 member SHARE Steering Committee included broad senior representation from executives, clinical and non-clinical program directors, committee chairs, legal counsel and consumer representatives. Major strategic decisions were approved by the Executive Management Team and the Monash Health Board, the program was an organisational priority, and the activities were integrated into the health service Business Plan.

The timing of the program was opportune as internal and external environments were amenable to exploration of disinvestment. The disinvestment literature was building, the DHS was exploring disinvestment at state level and local stakeholders were constructive in their responses. Monash Health had already demonstrated commitment and leadership to evidence-based decision-making (EBDM) by establishing the program for introduction of new TCPs [9]. The SHARE Program was able to capitalise on this momentum.

Staff and consumers were in agreement in their responses. Themes regarding current practice, proposals for change and barriers and enablers were strong and consistent across all participant groups. The key messages from participants were consistent with publications at the time and remain consistent with the current literature [8, 30].

The main limitations of the SHARE Program relate to generalisability, internal evaluation and loss of funding.

SHARE is a series of case studies from a single institution and there may be many points of difference with other health services. In particular, Australian public hospitals operate under a state-allocated activity-based fixed-budget model of financing [62], staff are salaried and are bound by organisational policies and procedures; all limiting the generalisability to other settings and models of health service delivery.

The SHARE model utilised in-house expertise in EBDM, knowledge brokerage and data analysis and engaged a health program evaluator and health economist as consultants; this level of expertise is unusual in the local health service context. While this was noted as a strength for SHARE, it limits generalisability to other settings that do not have access to this expertise. Although hospital-based resources for knowledge brokering are becoming more common [63, 64], they are not widespread, and the additional skills in implementation, evaluation and health economics are less common.

The project team delivering the SHARE Program were also the researchers investigating it. This has the potential to introduce subjectivity into evaluations and limit insight if organisational assumptions are accepted without challenge. Extensive stakeholder involvement, transparency of methods and participation of an external evaluator in the role of ‘critical friend’ [55] were included in the SHARE processes to minimise these limitations.

Funding was reduced in the final year of the program. As a result, some planned implementation and evaluation activities were not completed when the program concluded prematurely, limiting our ability to draw firm conclusions in some areas. Although Monash Health provided funding for the EDS after the loss of program funding, processing the volume of literature in the governance model was not sustainable.

## Contribution of the SHARE Program

These investigations in one local health service have produced important new contributions in several areas, which are captured in the tables and figures in Additional file 1. Some of these findings can be summarised as key messages or recommendations (Table 2).

**Table 2:** Key messages and recommendations

Disinvestment in general – key messages	Source^a^
▪ Understanding of systems, processes and influencing factors at the local health service level are important for successful disinvestment.	A
▪ Single definitions for disinvestment and health technologies, are needed with agreement between researchers, policy makers and health service decision-makers [8, 30]. We propose the following definitions. ‑ Disinvestment is removal, reduction or restriction of any aspect of the health system for any reason. Removal indicates complete cessation, reduction is a decrease in current volume or delivery sites, and restriction is narrowing of current indications or eligible populations. This is a broad definition, in essence the conceptual opposite of investment. It is an outcome of, rather than a reason for, a resource allocation decision. It is not burdened with the explanations and caveats of current research definitions. This could apply equally to products, devices and equipment; clinical practices and procedures; health services and programs; information technology and corporate systems. ‑ Health technologies are products, devices and equipment used to deliver health care (eg prostheses, implantable devices, vaccines, pharmaceuticals, surgical instruments, telehealth, interactive IT and diagnostic tools). This is a narrow definition which reflects the common use by decision-makers and consumers in the local health care setting. Clinical practices, support systems, and organisational and managerial systems are not considered to be health technologies in this context. ‑ Health technologies and clinical practices (TCPs) are therapeutic, diagnostic and preventative interventions (eg use of products, devices and equipment PLUS medical, surgical, nursing, allied health and population health activities). This is a pragmatic definition that reflects the scope of most resource allocation decisions related to delivery of health care in the local setting. ‑ Health programs and services are agencies, facilities, institutions and the components within them that deliver acute health care, rehabilitation or population health practices such as health promotion and education.	C
Disinvestment in general – recommendations	
▪ Avoid the term ‘disinvestment’, it is viewed negatively and perceived as ‘cost-cutting’. [8, 23, 26, 30]	A
▪ Do not to aim ‘to disinvest’ [8, 27] ‑ TCPs, services and programs that harm patients, diminish health outcomes, impair health care delivery, increase costs unnecessarily or result in organisational waste should be removed, reduced or restricted to address these adverse outcomes. ‑ If there are opportunities to replace TCPs, services and programs that are safe, effective and cost-effective with others that offer greater advantage no explanation is needed other than the expected benefit. ‑ If budgets are cut or funding is required for high priority activities it is worth remembering that health service staff place a high value on transparency and are disillusioned by attempts to disguise cost reduction methods.	A
▪ Do not develop ‘disinvestment’ as a health improvement strategy or research domain [8, 27]. ▪ Expand existing healthcare improvement paradigms and research domains (eg EBP, health technology assessment, guideline development, implementation science, knowledge translation, quality improvement, system redesign, health economics, etc) to address the need for theories, frameworks, methods and tools for [8, 23, 24, 26–30]: ‑ systematic and proactive identification of harmful, ineffective and inefficient TCPs, services and programs ‑ implementation of interventions to remove, reduce or restrict TCPs, services and programs ‑ evaluation of the process, impact and outcomes of these changes ‑ measurement of savings if possible ‑ reallocation of resources if appropriate	A
▪ The principles for a rigorous, evidence-based approach to decision-making for disinvestment in the context of all resource allocation decisions are incorporated into the Framework for an organisation-wide approach to disinvestment in the local healthcare setting (Figure 5)	A
Disinvestment in the local health service setting – key messages	
▪ Decisions to proceed with a project to implement change are often made without consideration of research evidence and local data and are not well-defined in terms of the intervention, practitioner group, patient population, indications, etc. ‑ Clinicians are frequently asked to undertake projects in their area of clinical expertise but they lack knowledge and skills in project management, implementation and evaluation. ‑ Clinicians are usually required to conduct a project in addition to their normal duties but without additional time or resources. ‑ Health service staff are well aware of their limitations and those of their colleagues in undertaking projects and they welcome advice and support. ‑ There are many decision-making settings and processes within health services ‑ There are many components in the research allocation process in addition to decision-making that need to be addressed ‑ There are insufficient resources and skills in decision-making, implementation and evaluation ‑ Staff need support	A
▪ Decision-making for resource allocation at the local level is not homogenous. Contrary to some assumptions in previous studies, there are multiple layers of decision-making with different actors, criteria, systems and processes. [24]	D
▪ There is a need for proactive methods to access and utilise high quality synthesised evidence in the research literature, routinely-collected local health service data and sources of consumer information to identify and drive disinvestment initiatives [23, 25, 30]	A
Disinvestment in the local health service setting – recommendations	
▪ Introduce a framework for an organisation-wide approach to disinvestment underpinned by evidence-based principles [30]	A
▪ Focus on optimising health care and using resource effectively rather than cost-cutting	A
▪ Implement systematic, transparent, evidence-based methods that integrate with, or build upon, existing decision-making systems and processes to identify TCPs that should be removed, reduced or restricted. [23, 30]	D
▪ Consider settings for decisions about both monetary (eg capital procurement and clinical purchasing) and non-monetary (eg development and authorisation of guidelines and protocols that stipulate use of drugs or equipment, recommend diagnostic tests, specify referral mechanisms etc) resources as opportunities to identify TCPs that should be removed, reduced or restricted. [23, 26, 27, 30]	D
▪ If seeking opportunities to save money by removing, reducing or restricting TCPs, use a systematic transparent process rather than *ad hoc* nominations from individuals. [8, 27]	A
▪ Ensure that proposals are fully developed before making decisions to proceed including consideration of research evidence and local data to determine the nature and scope of the problem and the most effective solution; clarification of the intervention and scope of the project in terms of practitioner group, patient population, indications, etc; and assessment of feasibility, risk and cost of implementation and evaluation. [28]	D
▪ Ensure appropriate knowledge and skills and adequate resources are available for effective project design, management and governance; implementation and evaluation	A
▪ Integrate activities to remove, reduce or restrict TCPs within the language and methods and tools of familiar health service improvement paradigms such as EBP, quality improvement and system redesign rather than the construct of ‘disinvestment’. [8, 24, 30]	A
▪ Include appropriate stakeholder consultation and involvement in making, implementing and evaluating decisions to disinvest. [25, 30]	A
▪ Develop mechanisms to receive and act upon consumer or community-initiated feedback on resource allocation decisions. [25]	D

^a^ Key

A: Based on findings from literature reviews, and local and/or expert respondents, and outcomes of SHARE investigations

B: Based on findings from literature reviews, and local and/or expert respondents, (*SHARE investigations incomplete due to local barriers or reduced timelines*)

C: Based on findings from literature reviews alone [8, 30], (*not investigated in SHARE projects*)

D: Based on findings of SHARE investigations alone, (*not found in other literature*)

Some of the contributions have been utilised at the source. We are pleased to report that many changes have been implemented at Monash Health following the SHARE Program. These are anecdotal findings, no additional evaluation has been conducted.

### New approaches

There are several differences in the way SHARE was conducted compared to other frequently reported approaches to disinvestment in the literature.

It is common for local healthcare facilities to make decisions within organisation-wide frameworks such as development and authorisation of policies and procedures, capital expenditure and clinical purchasing, introduction of new TCPs and models of care, and delivery of programs and services. However many published examples of disinvestment initiatives report individual standalone projects where the target has been identified in an isolated process independent of existing decision-making and project infrastructure. While this approach can potentially be successful, it can also contribute to lack of coordination, duplication, inconsistent messages and change fatigue within an organisation [1] and may result in unsuitable or unsustainable outcomes [26]. Monash Health chose to take an integrated, organisation-wide approach; using existing systems and processes to identify disinvestment opportunities or, when required, incorporating new methods into the existing infrastructure. The aims were to facilitate systematic identification of disinvestment opportunities, encourage consideration of disinvestment in routine decision-making and ensure the processes were transparent and accountable. This approach has been reiterated in more recent publications which propose that disinvestment activities are more likely to be successful if decisions are made at the local level, integrated into everyday decision-making and central to local planning [17, 20, 65, 66].

The concept of investment is rarely discussed in the disinvestment literature, yet in practice investment and disinvestment exist together [15, 16, 26]. Introduction of a new TCP provides a trigger to explore opportunities for disinvestment [13]. Investment without appropriate disinvestment can be wasteful and decisions about disinvestment made in isolation can be artificial and potentially counterproductive [23, 26]. The SHARE Program considered investment and disinvestment together as ‘resource allocation’ [24, 67]. This is an inclusive term that encompasses financial and other resources. It also draws the focus away from the negative perception that decisions to remove or reduce things are always about money and redirects it towards the more constructive approach that limited resources should be employed to achieve the best outcomes [26]. Many national and regional policies are now based on resource allocation and address both investment and disinvestment [68, 69].

Discussions about disinvestment and reinvestment are frequently focused on decisions about spending money, but many decisions in healthcare at the local level are about allocation of non-monetary resources such as staff time, capacity in clinics and operating suites, and use of tests and procedures; and they are often driven by considerations other than financial constraint [23]. Decisions about use of non-monetary resources are made by different people in different settings to financial decisions and opportunities for disinvestment will be overlooked if these are not addressed [24, 27, 28]. The SHARE Program investigated opportunities to identify TCPs suitable for disinvestment in settings allocating both monetary and non-monetary resources.

Due to the negative perceptions associated with the term ‘disinvestment’ Monash Health stakeholders and others propose that it is avoided [1, 15, 21, 26, 45, 70–72]. Systematic errors, organisational waste and inappropriate use of TCPs that are safe, effective and cost-effective when used correctly are also important at the local level, and in these cases many authors propose that consideration of ‘optimising health care’ is preferable to ‘disinvesting’ [15, 16, 71, 73–76]. The name and underpinning principles of the SHARE Program (Fig. 2) were designed to avoid the term ‘disinvestment’ and focus on the positive aspects of effective allocation of resources to optimise health outcomes.

We were not successful in avoiding the term ‘disinvestment’ in all aspects of the program, which contributed to one of the major learnings. In order to pilot disinvestment projects within the SHARE timelines we could not wait for the new systems and processes to be established to identify opportunities, hence we actively sought targets ‘to disinvest’. This process did not work in SHARE, or for others [13, 20, 27, 66, 77, 78]. Monash Health participants reported that previous projects to remove, reduce or restrict use of TCPs were established to reduce patient harm, medication error and unnecessary tests; standardise care; and save money and time; usually with more than one of these aims [24]. The SHARE literature review identified that, although there are few published examples of successful ‘disinvestment’ at the local level, there are many examples in the EBP and quality and safety literature where unsafe or ineffective TCPs have been discontinued [30]. While aiming ‘to disinvest’ does not appear to be effective at local level, cessation or limitation of current practices for more constructive reasons has been achieved successfully. Yet some of the current literature continues to encourage national health programs and local health services ‘to disinvest’ and promotes ‘disinvestment’ as a health improvement paradigm and research domain [30].

### New knowledge

The SHARE papers provide practical information from actual experiences in a local health service to guide others in similar situations and the case study format provides a level of detail not generally reported. The two literature reviews contribute to the body of knowledge regarding disinvestment and resource allocation from the perspective of the local healthcare setting.

Many of the findings of the SHARE Program were unexpected. The activities in Phase One were not originally planned but became necessary due to the lack of knowledge about local processes both within Monash Health and in the literature. It was anticipated that new systems and processes would be established to identify opportunities for disinvestment and successful disinvestment projects would be carried out in Phase Two. With a few exceptions, this did not happen. Yet SHARE was successful in meeting its aims (Fig. 2). The aims were to explore the nature of the innovations and methods to deliver them, evaluate the outcomes and understand what happened. Those thought to be feasible would be piloted and those found to be sustainable, effective and appropriate would be established as ongoing processes. Although some of the objectives were not achieved within the program timeframe, SHARE was successful in assessing acceptability and feasibility of the components and identifying methodological issues for implementation and evaluation. The findings of all these investigations provide a rich source of new information about decision-making in a local health service; methods to avoid in attempting disinvestment in this context; and settings, frameworks, models, methods and tools that have potential to enhance health care and warrant further exploration.

To the best of our knowledge, the SHARE papers are the first to report the following new findings.

#### Organisational decision-making

Little has been written about the systems and processes for organisational decision-making regarding resource allocation at the local level. The SHARE Program identified potential settings and mechanisms to integrate disinvestment into existing organisational infrastructure [23]; the type and scope of decisions and decision-makers authorised to act on behalf of the organisation and a taxonomy to classify them [24]; eight components of the resource allocation process, the structure and practice elements underpinning each component and the relationships between them [24]; strengths and weaknesses, barriers and enablers; and examples of decision-making criteria and evaluation data used in a healthcare setting [24].

In many studies of decision-making, participants were selected from the most senior positions in an organisation who are asked about resource allocation as if it was a homogenous process within their institution. SHARE identified that these decisions were made throughout the organisational hierarchy, different processes and criteria were used, and senior staff were often unaware of processes at other levels within the organisation [24].

Many types of decisions that are not generally discussed in the literature were also identified, all of which offer potential to explore and initiate disinvestment. Use of non-monetary resources is noted above. While much of the literature considers decision-making related to purchases of multi-million dollar equipment, little attention has been paid to decisions that spend millions of dollars on low-cost but frequently-used items such as cannulae, catheters, dressings and similar consumables which also offer disinvestment opportunities with potential for improved outcomes and significant cost saving.

#### Consumer participation

In contrast, much has been written about consumer participation, including resource allocation and disinvestment decisions. However the SHARE investigations identified two aspects of consumer participation in this context that were not found elsewhere [25]. Firstly, the literature focuses on consumer and community responses to health service initiatives, but the Monash Health consumer and community participants noted the additional need for mechanisms within health services to receive and act upon consumer-initiated contributions. Secondly, the concept of consumer evidence that could be searched in the same way as health research evidence was introduced. These are sources of consumer views and perspectives found in publications and data sources that can be used systematically and proactively to inform health service decisions [25]. These new findings were drawn together with findings from the literature into a model for consumer participation in resource allocation decision-making in the local setting.

#### Disinvestment process

Theoretical issues to consider in development of a disinvestment program in a local facility were collated in the SHARE planning phase [23] and then detailed implications for a program at Monash Health were ascertained from document analyses and interviews, surveys, workshops, and consultations with local stakeholders and external experts [26].

It has been proposed that in-depth research taking a longitudinal approach from project inception to completion of the disinvestment process at the health service level is needed [1, 20, 21, 74, 79]. The SHARE experience of disinvestment from identification, through prioritisation and decision-making, to implementation, evaluation and explication in one local health service is described in detail [27]. Unfortunately for the SHARE Program, the main messages arising from the process of identifying and deciding to proceed with a disinvestment project were about ‘what not to do’. Fortunately for others, this will enable them to avoid the mistakes, barriers and unanticipated events reported. On a more positive note, evaluation of the single project implemented found that it was underpinned by a rich list of enabling factors.

The literature review focusing on operationalising disinvestment reports definitions, concepts, principles, decision-making settings, potential prompts and triggers to consider disinvestment, and steps in the disinvestment process found in the literature and brings them together into a framework for organisation-wide application [30].

#### Addressing and understanding barriers and enablers

The barriers to EBDM and successful project management, implementation and evaluation of the resultant decisions are well documented and relate to all contexts, not just disinvestment and resource allocation. The SHARE Program piloted four in-house support services to address the lack of knowledge and skills in decision-makers and project staff and insufficient resources for project delivery [28, 29]. The education and training in EBP delivered by the Capacity Building Service is a well-researched area and there are other services disseminating evidence to subscribers. However we are unaware of other models similar to the Project Support Service or Evidence Dissemination Services being delivered in-house in a governance framework to facilitate disinvestment and ensure local practice is up-to-date. The local factors influencing decisions to develop these services and those influencing the processes and outcomes are provided in detail [28, 29].

The barriers and enablers to initiatives in the SHARE Program were investigated and reported using a framework and taxonomy for evaluation and explication adapted for use in decision-making processes, disinvestment projects and an in-house EDS, contributing to new knowledge in these areas.

### New resources

There are many resources arising from SHARE activities that may be useful for decision-makers, change agents, knowledge brokers and researchers to inform decisions, planning, implementation and evaluation in disinvestment and resource allocation programs (Table 3).

**Table 3:** Outputs of the SHARE Program (Reproduced with permission from SHARE Paper 1 [44])

Research questions	Outputs
SHARE 2: Identifying opportunities for disinvestment in a local healthcare setting	
▪ What concepts, definitions and perspectives underpin disinvestment? ▪ What models or methods of disinvestment have been implemented in hospitals or health services? ▪ Where are the opportunities for systematic decisions about disinvestment in a local health service network?	▪ Framework and detailed discussion of potential settings and methods for disinvestment in the local healthcare context ▪ Summary of issues to consider in development of an organisational program for disinvestment ▪ Interview protocol for ascertaining local implications for disinvestment
SHARE 3: Examining how resource allocation decisions are made, implemented and evaluated in a local healthcare setting	
▪ Where, how and by whom are decisions about resource allocation made, implemented and evaluated at Monash Health? ▪ What factors influence these processes? ▪ What knowledge or experience of disinvestment exists within Monash Health?	▪ Framework of eight components in the research allocation process, the elements of structure and practice for each component, and the relationships between them ▪ Classification of decision-makers, decision-making settings, type and scope of decisions, strengths and weaknesses, barriers and enablers ▪ Examples of decision-making criteria and types and sources of evaluation data used ▪ Interview and workshop protocols for ascertaining local decision-making systems and processes
SHARE 4: Exploring opportunities and methods for consumer engagement in resource allocation in a local healthcare setting	
▪ How can consumer and community values and preferences be systematically integrated into organisation-wide decision-making for resource allocation?	▪ Model for integrating consumer values and preferences into decision-making for resource allocation ▪ Definitions for consumer engagement terminology ▪ Examples of sources of consumer information and data ▪ Examples of consumer-related activities generating proactive decisions to drive change
SHARE 5: Developing a model for evidence-driven resource allocation in a local healthcare setting	
▪ What are the implications for disinvestment at Monash Health? ▪ What is the most appropriate and effective approach to organisation-wide, systematic, integrated, evidence-driven disinvestment at Monash Health? ▪ Can a model for evidence-driven resource allocation in the local healthcare setting be derived from the Monash Health program to enable replication and testing?	▪ Model for exploring Sustainability in Health care by Allocating Resources Effectively in the local healthcare setting ▪ Definition of four program components, aims and objectives, relationships between components, principles that underpin the program, implementation and evaluation plans, and preconditions for success and sustainability. ▪ Summary of implications for disinvestment in the local setting and resulting decisions for program development ▪ Summary of factors for program sustainability ▪ Evaluation framework and plan
SHARE 6: Investigating methods to identify, prioritise, implement and evaluate disinvestment projects in a local healthcare setting	
▪ What methods are available to identify potential disinvestment opportunities in a local health service? ▪ What methods are available for prioritisation and decision-making to initiate disinvestment projects in a local health service? ▪ What methods are available to develop, implement and evaluate disinvestment projects in a local health service? ▪ What were the processes and outcomes of application of these methods at Monash Health? ▪ What factors influenced the decisions, processes and outcomes?	▪ Framework for evaluation and explication of a disinvestment project ▪ Examples of criteria for selection of disinvestment projects ▪ Methods for developing an evidence-based catalogue of potential disinvestment opportunities ▪ Algorithm for selecting a disinvestment project from an evidence-based catalogue of potential disinvestment opportunities ▪ Summary of barriers and enablers to implementation and evaluation ▪ Summary of factors related to determinants of effectiveness arising in SHARE process and disinvestment projects
SHARE 7: Supporting staff in evidence-based decision-making, implementation and evaluation in a local healthcare setting	
▪ What is current practice in accessing and using evidence for making, implementing and evaluating decisions at Monash Health? ▪ What decisions were made and outcomes achieved in the piloting of support services? ▪ What factors influenced the decisions, processes and outcomes?	▪ Matrix of barriers, enablers, additional needs and evidence-based interventions mapped to their corresponding components in four support services to enable evidence-based decision-making, implementation and evaluation ▪ Summary of factors influencing decision-making for development of support services ▪ Summary of factors influencing the outcomes of the SHARE support services piloting process ▪ Summaries of current practice, knowledge, skills, confidence and needs in finding, accessing and using evidence for making, implementing and evaluating decisions; and preferred formats for education and training ▪ Summaries of nature, type and availability of local health service data; data sources; uses and expertise available ▪ Evaluation framework and plan
SHARE 8: Developing, implementing and evaluating an Evidence Dissemination Service in a local healthcare setting	
▪ What are the potential features of an Evidence Dissemination Service in a local healthcare setting? ▪ How can high quality synthesised evidence be identified, captured, classified, stored, repackaged and disseminated? ▪ How can disseminated evidence be used to enhance current practice and how can use of evidence be reported? ▪ What are the processes and outcomes of disseminating evidence to self-selected and targeted participants in a voluntary framework? ▪ What are the processes and outcomes of disseminating evidence to designated decision-makers in a mandatory governance framework? ▪ What factors influenced the decisions, processes and outcomes?	▪ Two models for an Evidence Dissemination Service (EDS) in a local healthcare service ▪ Methods for identification, capture, classification, storage, repackaging and dissemination of evidence ▪ Methods to facilitate use of disseminated evidence and reporting of outcomes ▪ Taxonomy for categorising publications ▪ Framework for evaluation and explication of implementation of health information products and services ▪ Summaries of factors influencing decisions, processes and outcomes in development and delivery of the EDS
SHARE 9: Conceptualising disinvestment in a local healthcare setting	
▪ Aims: To discuss the current literature on disinvestment from a conceptual perspective, consider the implications for local healthcare settings and propose a new definition and two potential approaches to disinvestment in this context to stimulate further research and discussion.	▪ Discussion of the disinvestment literature in relation to terminology and concepts, motivation and purpose, relationships with other health improvement paradigms, challenges, and implications for policy, practice and research in local healthcare settings
SHARE 10: Operationalising disinvestment in a conceptual framework for resource allocation	
▪ Aims: To discuss the current literature on disinvestment from an operational perspective, combine it with the experiences of the SHARE Program, and propose a framework for disinvestment in the context of resource allocation in the local healthcare setting.	▪ Discussion of the disinvestment literature from an operational perspective in local healthcare settings ▪ Summary of theories, frameworks and models used in disinvestment-related activities ▪ Framework for evidence-based disinvestment in the context of resource allocation - Standardised definitions and concepts to underpin framework - Principles for resource allocation decision-making - Potential activities and settings for disinvestment - Potential prompts and triggers to initiate disinvestment decisions - Methods and tools for disinvestment - Barriers to disinvestment
SHARE 11: Reporting outcomes of an evidence-driven approach to disinvestment in a local healthcare setting	
▪ Aims: To consolidate the findings, discuss the contribution of the SHARE Program to the knowledge and understanding of disinvestment in the local healthcare setting, and consider the implications for policy, practice and research.	▪ Summary of outcomes of the SHARE Program ▪ Key messages ▪ Implications for research, policy and practice
SHARE National Workshop	
▪ Aim: To share knowledge of disinvestment and develop links for future collaborative work opportunities	▪ Summary of disinvestment activities from health policy, health economics and health service perspectives ▪ Tools for group activities discussing disinvestment concepts and decision-making ▪ Tools for individual activities to capture information about current practice and research in disinvestment ▪ Workshop presentations ▪ Workshop evaluation tool and findings ▪ Summary of key messages

The new knowledge arising from the SHARE findings was used to create four frameworks, three models and an algorithm, and develop several adaptations of an existing framework.

Inconsistent use of terminology was common in several of the areas investigated, and in other areas new terminology was needed to fill a gap. Definitions were provided for terms used in SHARE projects, frameworks and models.

The protocols and instruments used in SHARE surveys, interviews, workshops and literature reviews may be useful to others wishing to ascertain similar information.

Summaries, lists and tables capture the findings across a range of areas including current practice; staff knowledge, skills, confidence and needs; factors influencing decision-making; and barriers and enablers.

## Implications for policy, practice and research

Some of the implications for policy, practice and research can be summarised as key messages or recommendations (Table 2).

### Recognising the relevance of the local healthcare perspective

Resource allocation and disinvestment decisions can be made centrally, but implementation is likely to require change locally [65, 80, 81]. In addition, national recommendations cannot take into account local factors such as population needs, organisational priorities, budgets, capacity or capability; hence many decisions about the use of TCPs, programs and services have to be made at the local level [9]. The challenges inherent in disinvestment processes [8], particularly those related to implementation, may have the greatest impact in the local setting.

The importance of exploring disinvestment at the local level is noted in the disinvestment literature [17, 20, 79, 82–84]. Specific examples include: identifying determinants for disinvestment [18, 20, 85]; implementing change management [15, 84]; drafting and refining frameworks, methods and tools [12, 13, 15, 16, 18, 19, 70, 85]; and measuring impact, potential unintended consequences and factors contributing to success or failure of disinvestment initiatives [13, 74, 83].

The SHARE Program provides some early work to build on by reporting disinvestment projects from inception to implementation [27]; identifying determinants for disinvestment, potential unintended consequences and factors contributing to success or failure [27]; and developing frameworks, models and algorithms [23–27, 29] and evaluation frameworks and plans [28, 29, 55]. These outputs of the SHARE Program are discussed in Paper 1 [44] and summarised in Table 3.

### Aligning definitions

The SHARE literature reviews highlight the lack of agreement of not only the definitions, but the concepts underpinning the definitions of ‘health technologies’ and ‘disinvestment’. A common understanding is required for successful decision-making and communication in policy and practice settings. A consistent definition is also important for implementation and evaluation of change in the practice setting and activities in the research domain to enable replication and comparison with others.

Definitions that reflect use of these terms at the local level are quite different from current research definitions. This disparity may lead to confusion or misunderstanding and hamper knowledge translation in this area. Definitions developed from the local perspective are included in Table 2 and the Additional file.

### Enhancing organisational decision-making, implementation and evaluation

Although quality improvement processes for clinical practice and service delivery are well-established and routinely conducted in healthcare facilities, ongoing evaluation and enhancement of organisational decision-making processes is not common practice [9]. Similarly, much of the research in evidence-based health care has been conducted in the clinical domain resulting in a substantial body of knowledge translation strategies for health professionals, but the main focus of disinvestment has been in policy and management activities where the evidence for knowledge translation is much weaker [86–88]. The frameworks, models, methods and tools; classifications of decision-makers, decision-making settings, type and scope of decisions; and lists of strengths, weaknesses, barriers, enablers and needs that emerged from the SHARE research could assist policy makers, managers, clinicians and researchers to improve these processes.

The SHARE findings confirmed the importance of appropriate skills and adequate time and resources for development, implementation and evaluation of innovations; yet this remains a constant tension in health services [77, 89–94]. Responses to emerging problems are frequently urgent and reactive, delivered by staff with limited experience in project management or change strategies, with inadequate resources and inappropriate timelines, resulting in projects that are not implemented or evaluated effectively [21, 24, 27–29]. The SHARE findings reinforce the need for expertise and practical support; access to relevant methods and tools; and education, training and capacity-building within a local health service [17, 19, 82, 95, 96].

The lack of explicit criteria and limited use of evidence in decision-making; lack of skills and resources to make, implement and evaluate evidence-based decisions; and minimal consumer involvement that were identified in the SHARE investigations are not unique to Monash Health and have been reported in health services around the world [1, 11, 21, 76, 97–100]. The prevalence of these issues highlights the extent of the problem and the considerable potential for improvement in these areas.

### Developing proactive processes to initiate evidence-based disinvestment

Although a lack of frameworks, models, methods and tools for disinvestment is reported in the literature [12, 13, 16, 18, 19, 70, 74, 101–103], the SHARE reviews identified some frameworks and models specifically for disinvestment, and many methods and tools from other research disciplines which are relevant for disinvestment projects [30]. However there is a lack of proactive mechanisms, prompts and triggers to drive disinvestment initiatives [11, 13, 21, 27, 29, 82, 104]. High quality synthesised evidence is available in systematic reviews, HTAs and evidence-based guidelines and there are rigorous methods for analysis of routinely-collected health service data [23]; but no systematic proactive methods to access existing information, initiate the processes or draw the results to the attention of health service decision-makers were identified [30]. It is also not clear who is, or should be, responsible for instigating and making decisions and taking action [23].

The SHARE model for exploring resource allocation in the local setting [26], algorithm for identifying suitable projects from a database of disinvestment opportunities [27], and methods for proactively delivering research evidence and local data to decision-makers [28, 29] could be used to inform future work and address the recognised gaps in these areas.

### Adapting, testing and refining SHARE innovations

Many of the SHARE findings are the first of their kind and therefore require confirmation or refutation in subsequent studies. The new framework for resource allocation provides a basis on which to build a systematic approach to further investigation of disinvestment processes [30].

Although some of the original aims of the SHARE Program were not achieved, the barriers were largely due to unique local circumstances at the time of implementation. Since the planned interventions were all based on evidence from rigorous reviews of published literature and extensive local research, and most of the barriers were local and project-specific, these initiatives still hold promise as systematic ways to reduce practices that are harmful, of little benefit or where there are more effective or cost-effective alternatives in the local setting. In other situations, or with other methods of investigation and implementation, they may prove to be effective tools. In contrast, some of the unplanned activities undertaken in the SHARE Program highlight approaches that should probably be avoided in development of future interventions. The evaluation and explication processes have identified the positive and negative influencing factors for each of the SHARE innovations. These details could inform future replication, adaptation, testing and refinement in a range of policy, practice and research contexts.

The frameworks and models can be tested in clinical, management or policy contexts at the local level; for disinvestment, resource allocation or other decision-making processes. They are each based on multiple components and the relationships between them. A range of hypotheses could be developed for the components and their relationships which could be tested in a number of ways using various methodologies.

## Conclusion

The SHARE papers provide practical information from actual experiences in a local health service to inform others in similar situations and the case study format provides a level of detail not generally reported. Although some of the objectives were not achieved, SHARE was successful in assessing acceptability and feasibility of multiple innovations related to disinvestment in the local health service setting and identifying factors influencing implementation and evaluation. The findings of these investigations provide a rich source of new information about decision-making in a local health service; methods to avoid in attempting disinvestment in this context; and settings, frameworks, models, methods and tools that have potential to enhance health care and warrant further exploration.

## Additional file

Additional file 1: Summary of findings. (PDF 3336 kb)

## Abbreviations

CCE: Centre for Clinical Effectiveness
EBDM: Evidence-based decision-making
EBP: Evidence-based practice
EDS: Evidence Dissemination Service
EOI: Expression of Interest
SHARE: Sustainability in Health care by Allocating Resources Effectively
TCPs: Technologies and clinical practices
