# Supplementary material for: Sustainability in Health care by Allocating Resources Effectively (SHARE) 11: reporting outcomes of an evidence-driven approach to disinvestment in a local healthcare setting
Source: BMC Health Serv Res. 2018 May 30;18:386. doi: 10.1186/s12913-018-3172-0 (PMC5975394; doi:10.1186/s12913-018-3172-0)
Supplement: Supplementary file 1 — Summary of findings. (PDF 3336 kb) [file 12913_2018_3172_MOESM1_ESM.pdf]

The SHARE Program (Sustainability in Health care by Allocating Resources Effectively) 11: Reporting outcomes of an evidence-driven framework for resource allocation in a local healthcare setting

Additional File: Summary of findings

CONTENTS

FIGURES 1

TABLES 2

PHASE ONE (SHARE Papers 2-5) 3

- Specifying the context 3
- Understanding the problem 4
- Defining the components 21

PHASE TWO (SHARE Papers 6-8) 25

- Aim 1. Systems and processes 25
- Aim 2. Disinvestment projects 28
- Aim 3. Support services 36
- Aim 4. Program evaluation and research 48

PHASE THREE (SHARE Papers 9 & 10) 51

- Terminology and concepts 51
- Motivation and purpose 53
- Relationship with other healthcare improvement paradigms 55
- Challenges 55
- Redefining disinvestment 55
- Theories, frameworks and models 55
- New framework for an organisation-wide approach to disinvestment in the local healthcare setting 59
- Key findings and recommendations 59

REFERENCES 71

FIGURES

Figure 1. Framework for potential mechanisms to integrate disinvestment into health service systems and processes . 4

Figure 2. Draft frameworks for SHARE Program 6

Figure 3. Framework for the resource allocation process in a local health service 7

Figure 4. Model for integrating consumer values and preferences into the resource allocation process 18

Figure 5. Model for exploring sustainability in health care by allocating resources effectively in the local setting 24

Figure 6. Overview of activities for SHARE Aims 1 and 2 26

Figure 7. Algorithm for identifying disinvestment projects from an evidence-based catalogue of potential TCPs 29

Figure 8. Overview of investigation of the SHARE Support Services 36

Figure 9. Development, implementation and evaluation of an in-house Evidence Dissemination Service 40

Figure 10. Comparison of stakeholder roles in two models for an in-house Evidence Dissemination Service 41

Figure 11. Example of an Evidence Bulletin 42

Figure 12. Four adaptations of a framework for evaluation and explication 48

Figure 13. Relationships between reasons for disinvestment 54

Figure 14. Framework for an organisation-wide approach to disinvestment in the local healthcare setting 60

TABLES

Table 1. Issues to consider in development of an organisational program for disinvestment 3
Table 2. Decision-makers and scope and types of decisions for resource allocation 5
Table 3. Structure and practice elements of components of the resource allocation process 7
Table 4. Strengths, weaknesses, barriers and enablers in decision-making for resource allocation 8
Table 5. Examples of criteria for resource allocation decisions 16
Table 6. Examples of types and sources of evaluation data used by committees 17
Table 7. Differences in decision-making between health professional groups 17
Table 8. Examples of consumer-related activities generating proactive decisions to drive change 19
Table 9. Examples of routinely-collected consumer data 19
Table 10. Examples of publications with consumer information 20
Table 11. Definitions of consumer-related terms 21
Table 12. Factors that influenced decisions for SHARE program development 22
Table 13. Additional systematic methods to identify disinvestment opportunities in a local health service 29
Table 14. Disinvestment projects proposed in the SHARE Program 30
Table 15. Examples of criteria for selection of disinvestment projects considered in the SHARE Program 31
Table 16. Factors for success, sustainability and suitability for disinvestment in the SHARE pilot project 31
Table 17. Factors influencing the SHARE process of selecting disinvestment projects 32
Table 18. Factors influencing the SHARE pilot disinvestment project 34
Table 19. Factors that influenced decisions in development of the SHARE support services 37
Table 20. Factors that influenced processes and outcomes of the SHARE support services 39
Table 21. Unique characteristics of the SHARE EDS 44
Table 22. Activities of the Capacity Building Service 46
Table 23. Activities of the Project Support Service 47
Table 24. Contents of the literature overviews 51
Table 25. Examples of definitions for disinvestment 52
Table 26. Examples of use of the term ‘health technologies’ 52
Table 27. Examples of alternatives for the term ‘disinvestment’ 53
Table 28. Examples of reasons for disinvestment from the literature 53
Table 29. Potential reasons for disinvestment in the local healthcare setting 54
Table 30. Examples of frameworks and models related to disinvestment 56
Table 31. Definitions underpinning the framework for an organisation-wide approach to disinvestment 61
Table 32. Concepts underpinning the framework for an organisation-wide approach to disinvestment 62
Table 33. Principles for a program of decision-making for resource allocation 63
Table 34. Examples of activities and settings for disinvestment within decision-making infrastructure 66
Table 35. Examples of systematic prompts and triggers to initiate disinvestment decisions 67
Table 36. Examples of potential barriers to disinvestment 68
Table 37. Key messages and recommendations 69

PHASE ONE (SHARE Papers 2-5)

Based on the UK Medical Research Council framework for complex interventions [1], Phase One involved specifying the context, understanding the problem and defining the components of an optimal intervention. The findings are presented and discussed in the context of the current literature.

Specifying the context

Several factors influenced early decisions regarding the scope and direction of the program.

The search for models, methods, theoretical guidance or practical advice for an organisation-wide approach to disinvestment was fruitless; however a range of issues to consider in development of a local program was identified (Table 1) [2].

Table 1. Issues to consider in development of an organisational program for disinvestment

Reproduced with permission from SHARE Paper 2 [2]

Topic	Issues
Organisational and management	▪ How can a systematic evidence-based approach to disinvestment be implemented in a healthcare organisation?<br>▪ How can disinvestment decisions be integrated into established Strategic and Business Plans<br>▪ Which is the better approach – 'top down', 'bottom up' or both?<br>▪ How to engage and get 'buy-in' from clinicians, consumers and other stakeholders?<br>▪ What are the relevant organisational change mechanisms?<br>▪ What does leadership for disinvestment involve?
Decision-makers	▪ Who has the authority, and the will, to make and act upon decisions about disinvestment?<br>▪ Who are the appropriate decision-makers?<br>· Existing decision-making bodies or specially convened groups<br>· Composition: policy-makers, managers, clinicians, consumers, technical experts, others<br>· In-house or external<br>▪ How does the relevant information get to them?<br>▪ What other agendas do they bring to the decision-making table?<br>▪ Who has the time, relevant skills and adequate resources to identify, implement and evaluate the required practice changes?
Decision-making	▪ Are all viewpoints equal?<br>▪ What criteria should be applied to disinvestment decisions and prioritisation?<br>▪ What is the nature and source of information required?<br>▪ How do decision-makers become aware of the need to disinvest certain practices?<br>▪ How are policies and guidance documents used by local decision-makers to allocate resources?
Assumptions	▪ Are generally held assumptions true? For example<br>· 'Clinicians are reluctant to disinvest'<br>· 'Disinvestment is not optimal unless an active intervention is in place'
Skills and resources	▪ What expertise and training is required to make, communicate, implement and evaluate decisions?<br>▪ What resources are required to source expertise, source information, 'backfill' health service staff when participating, and support decision-making, implementation and evaluation processes?
Professional and cultural	▪ What impact will professional boundaries and 'turf' issues have on disinvestment activities?<br>▪ What are the rights and responsibilities of stakeholders?<br>▪ Different stakeholder views of what is meant by 'little or no health benefit'<br>▪ What is the effect of culture on disinvestment? (authoritative versus consultative, transparent versus hidden)<br>▪ What are the motives and incentives for disinvestment?
Financial and commercial	▪ What funding is required for disinvestment initiatives and where can it be found?<br>▪ How can the difficulties inherent in the complex funding arrangements within health services be overcome?<br>▪ How can savings be measured?<br>▪ How can savings be reinvested?
Values and ethics	▪ How can transparency of process be ensured?<br>▪ What is a 'fair and reasonable' process?<br>▪ What are the access, equity and legal considerations?<br>▪ What is the best way to deal with conflict of interest with commercial entities?
Research and evaluation	▪ What effect will the limited evidence base for some practices have on the process?<br>▪ How can the lack of tested methods for implementation and evaluation be addressed?

There was a lack of common terminology regarding definitions and concepts related to disinvestment. The only consistent message from the literature, confirmed by local stakeholders at the time and reiterated in more recent publications, was that the term 'disinvestment' had strong negative connotations and was likely to be a barrier to successful disinvestment-related change [3-8]. It was agreed that the term would be avoided.

It is common for local healthcare facilities to make decisions within organisation-wide frameworks such as development and authorisation of policies and procedures, capital expenditure and clinical purchasing, introduction of new technologies and clinical practices (TCPs) and models of care, and delivery of programs and services. However many

published examples of disinvestment initiatives report individual standalone projects where the target has been identified in an isolated process; all conducted independently of existing decision-making and project infrastructure. While this approach can potentially be successful, it can also contribute to lack of coordination, duplication, inconsistent messages and change fatigue within an organisation [9]. Making decisions to disinvest in separate processes to those for investment decisions was also thought to be artificial and possibly counterproductive [2]. Monash Health chose to take an integrated, organisation-wide approach; using existing systems and processes to identify disinvestment opportunities or, when required, incorporating new methods into the existing infrastructure. Disinvestment would be considered alongside investment in the context of all resource allocation decisions. The aim was to facilitate systematic identification of disinvestment opportunities, encourage consideration of disinvestment in routine decision-making and ensure the processes were transparent and accountable. This approach has been reiterated in more recent publications which propose that disinvestment activities are more likely to be successful if decisions are made at the local level, integrated into everyday decision-making and central to local planning [10-13].

Much of the previous research on disinvestment had been driven by health economic principles. Monash Health did not have in-house capability in health economics, but did have high level expertise in accessing and utilising evidence from published research and local data. It was decided that the program would be driven from an evidence-based, rather than economics-based, perspective.

Hence the 'Disinvestment Project' became the 'Sustainability in Health care by Allocating Resources Effectively' (SHARE) Program, an organisation-wide, systematic, integrated, transparent, evidence-based approach to disinvestment in the context of resource allocation.

Preliminary research found that Monash Health did not have any decision-making settings where 'disinvestment' was explicitly considered; therefore new systems and processes were needed. Since no guidance was available, a conceptual framework of potential mechanisms to introduce disinvestment decisions into health service infrastructure was developed based on the knowledge and experience of local stakeholders [2]. Three potential mechanisms for a systematic approach to disinvestment decisions were identified: 1) Explicit consideration of potential disinvestment in routine decision-making such as purchasing and procurement and guideline and protocol development, 2) Proactive decision-making about disinvestment driven by available evidence from published research and local data, and 3) Specific exercises in priority setting and system redesign (Figure 1).

Figure 1. Framework for potential mechanisms to integrate disinvestment into health service systems and processes

Reproduced with permission from SHARE Paper 2 [2]

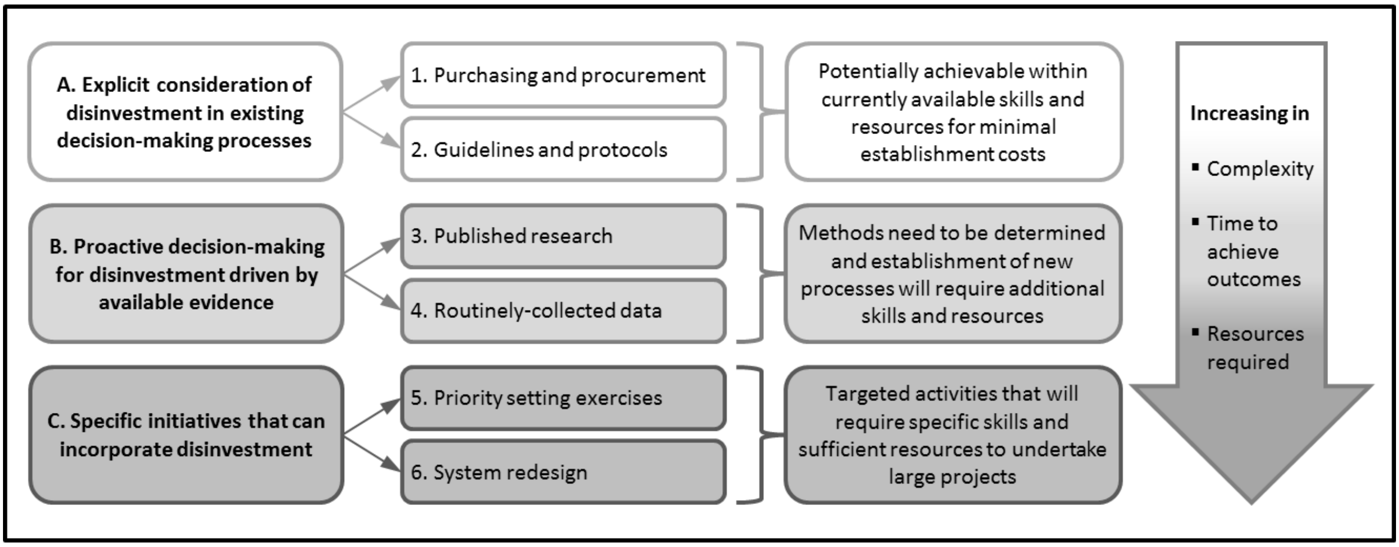

Understanding the problem

In order to introduce the proposed organisation-wide program, knowledge of existing decision-making systems and processes for investment and disinvestment was required.

While there was a broad understanding of where resource allocation decisions were made at Monash Health, detailed knowledge of who made them and how they were made, implemented and evaluated was lacking, and this information was also unavailable in the literature [14]. Detailed responses to surveys, interviews and workshops from a wide range of participants enabled identification of, and development of classifications for, decision-makers, decision-making settings, and type and scope of decisions (Table 2).

Table 2. Decision-makers and scope and types of decisions for resource allocation

Reproduced with permission from SHARE Paper 3 [14]

DECISION-MAKERS

Clinicians

Health practitioners delivering patient care.

Authorised individuals

Authorised individuals include Board Members, Executive Directors, Directors and Managers at all levels within the organisation. They are designated by their role in the organisation, for example 'Director of Pharmacy', rather than as a named individual 'John Smith'.

Authorised groups

Authorised groups can be classified into those with

- ongoing roles and responsibilities for decisions such as the Board, Executive Management Team, Standing Committees, Approved Purchasing Units and Profession-specific groups such as the Nursing Executive.
- a specific, often time-limited, purpose such as a project Steering Committee, a Procurement Evaluation Committee to purchase a large piece of equipment and special initiatives like the High Cost Drugs Working Party of the Therapeutics Equivalence Program.

SCOPE OF DECISIONS

Clinicians make decisions for individual patients within the limits of parameters outlined in their position description, relevant professional standards and any local credentialing requirements.

Authorised individuals and groups make decisions on behalf of the organisation which impact on all patients, all staff or identified subgroups.

Individuals are authorised to make decisions on behalf of the organisation within a range of specified parameters outlined in their position description or the Authority Delegation Schedule.

Committees and other groups are authorised to make decisions on behalf of the organisation as stipulated in their Terms of Reference.

Examples of the parameters decision-makers are authorised to work within include, but are not limited to, location (eg South East sites), professional group (eg occupational therapists), specialty area (eg stomal therapy), patient group (eg children), nature of purchase or resource use (eg surgical equipment and consumables) and cost limit (eg up to $10,000).

TYPES OF DECISIONS

Clinical

- Clinical decisions arise in the encounter between a health practitioner and an individual patient or client. Their purpose is to assess, treat and/or plan ongoing management of a health issue.

Strategic, operational or professional

- Strategic decisions point the organisation in the direction it wants to go; they are captured in strategic goals and policies which reflect a particular position, priority or plan the organisation wishes to communicate to staff, patients and other stakeholders. Strategic planning is usually undertaken at organisation-level driven by the Board, Executive and Senior Managers but can also be undertaken at any level.
- Operational decisions make the strategic goals happen; they enable day-to-day operations and are undertaken by managers at all levels.
- Professional decisions address standards and methods of practice and are made by senior staff in the discipline to which they are relevant.

Routine, reactive or proactive

- Routine decisions are made on a regular basis; examples include annual budget setting processes, monthly committee meetings and reviews of guidelines or protocols at specified intervals after their introduction.
- Reactive decisions are made in response to situations as they arise; for example new legislation, product alerts and recalls, critical incidents and applications for new drugs to be included in the formulary.
- Proactive decisions are driven by information that was actively sought for this purpose such as accessing newly published research evidence to compare against current practice or interrogating local data to ascertain practices with high costs or high rates of adverse events.

Conditional or unconditional

- Conditional decisions specify requirements to be met before or after their implementation; for example availability of funding, clinical indications (eg disease/condition, severity, patient group), authorised practitioners (eg specific training, named individuals), monitoring of outcomes (eg patient outcomes, adverse events, costs), location (eg ICU, Hospital in the Home), time limitation (eg until 2 year review).
- Unconditional decisions have no requirements.

Allocating funds or non-monetary resources

- Allocating funds involves spending money or putting it aside to purchase specified items later.
- Allocating non-monetary resources can include rostering staff time; specifying health professional groups; providing clinic or operating room time; and developing protocols that direct use of clinical interventions, equipment, drugs, diagnostic tests and referral mechanisms.

Whether to buy or what, where and how to buy

- 'Whether to buy' is a decision about what is required, for example a new drug to improve patient outcomes, a new scanner to reduce waiting time, consumables for a piece of equipment in current use. These decisions are undertaken by authorised individuals and some of the authorised groups such as the Technology/Clinical Practice Committee, Therapeutics Committee, Falls Prevention Committee, etc.
- 'What, where and how to buy' is a decision about how the requirement is met and considers product and manufacturer reliability, availability of parts and tools, service and maintenance contracts, IT requirements for hardware and software, price negotiations, etc. These decisions are undertaken by the Approved Purchasing Units and groups established for specific purchases.

Purchase of budgeted or unbudgeted items

- Decisions to purchase budgeted items are made by the relevant authorised individual, usually the budget holder or their line manager depending on the purchase price and the designated cost limits of their respective approval levels (eg <$10,000, <$50,000).
- Decisions to purchase unbudgeted items can only be approved by specified committees and Executive Directors

This investigation found that most decisions to introduce or remove TCPs were usually made ‘reactively’ in response to internal applications or external notifications, and highlighted the opportunity to drive decisions ‘proactively’ using information that was specifically ascertained to identify potential for improvement.

Several of these early findings were combined in a draft framework, which was refined to include subsequent decisions, and used to seek endorsement from the Monash Health Board and apply for funding from the state health department (Figure 2).

Figure 2. Draft frameworks for SHARE Program

Reproduced with permission from SHARE Paper 5 [3]

Initial draft

Introduction of safe, effective, cost-effective TCPs	Removal of harmful, ineffective, inefficient TCPs
Reactive (current)<br>• Application process<br>Proactive (potential)<br>• Identification of evidence regarding new TCPs that are safer, more effective or more cost-effective	Reactive (current)<br>• Drug alerts, product withdrawals<br>Proactive (potential)<br>• Identification of evidence regarding TCPs in current practice that are less safe, less effective or less cost-effective

Revised draft

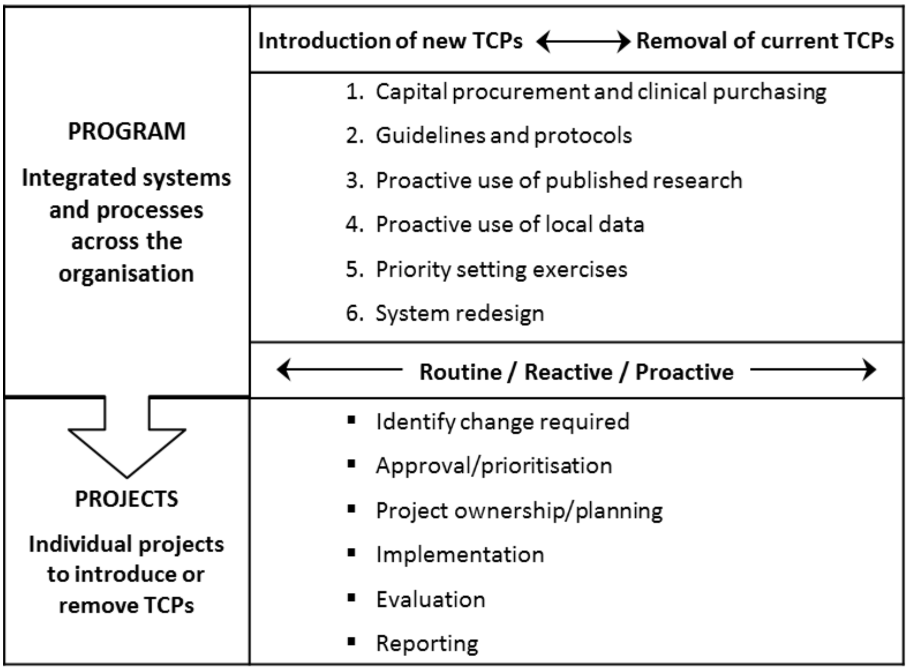

In addition to the specific activities of making, implementing and evaluating decisions, health service staff noted several other components in the process of resource allocation. Eight components were identified: Governance, Administration, Stakeholder engagement, Resources, Decision-making, Implementation, Evaluation and, where appropriate, Reinvestment of savings [14]. The detailed responses enabled elements of structure and practice for each component to be defined and a framework capturing the relationships between them to be produced (Figure 3 and Table 3). Strengths, weaknesses, barriers and enablers to the resource allocation process were identified, as well as examples of criteria used by different decision-making groups, the types and sources of data used in evaluation, and differences in the decision-making processes and information needs of medical, nursing, allied health and management/support groups (Tables 4-7).

The term ‘disinvestment’ was generally unfamiliar to local decision-makers; but the concept was readily understood and projects involving removal, reduction or restriction of current practices or reallocation of resources were easily identified [14]. At Monash Health, these activities were initiated by quality and safety issues, evidence-based practice (EBP), or a need to find resource savings, and not by a primary aim ‘to disinvest’ [14].

Figure 3. Framework for the resource allocation process in a local health service
Reproduced with permission from SHARE Paper 3 [14]

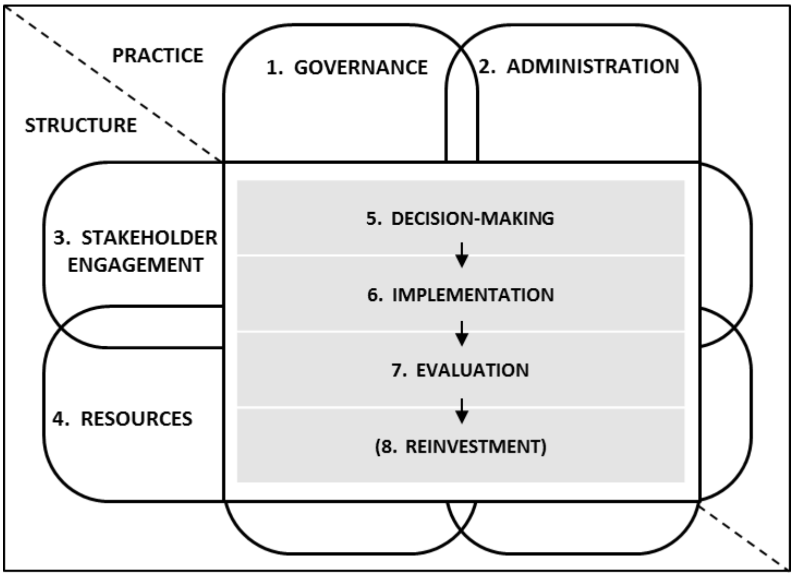

Table 3. Structure and practice elements of components of the resource allocation process
Reproduced with permission from SHARE Paper 3 [14]

COMPONENTS	STRUCTURE (Who, What)	PRACTICE (How)
**1. Governance**	▪ Overseers<br>▪ Policies for decision-making<br>▪ Transparency and accountability in all structures<br>▪ Requirements for addressing conflict of interest*<br>▪ Requirements for monitoring, evaluation and improvement of systems and processes<br>▪ Requirements for reporting	▪ Oversight<br>▪ Procedures, guidelines, protocols for decision-making<br>▪ Transparency and accountability in all practices<br>▪ Methods of addressing conflict of interest<br>▪ Methods of monitoring, evaluation and improvement of systems and processes<br>▪ Methods of reporting
**2. Administration**	▪ Administrators<br>▪ Requirements for administration<br>▪ Relationships and coordination<br>▪ Communication	▪ Methods of administration, coordination, communication and collaboration
**3. Stakeholder engagement**	▪ Clinicians, Managers, Consumers, Technical experts, Funders, other relevant parties<br>▪ Requirements for stakeholder engagement	▪ Methods of identification, recruitment and engagement
**4. Resources**	▪ Funding sources<br>▪ Allocation of staff<br>▪ Access to experts or ways to gain expertise<br>▪ Information sources<br>▪ Requirements for resources	▪ Provision of appropriate and adequate funding, time, skills/training, information<br>▪ Utilisation of resources
**5. Decision-making**	▪ Decision-makers<br>– Clinicians<br>– Authorised individuals<br>– Authorised groups<br>▪ Scope of decisions<br>▪ Type of decisions<br>▪ Requirements for decision-making	▪ Methods of decision-making<br>– Identification of need/application<br>– Decision criteria<br>– Ascertainment and use of evidence<br>– Reminders and prompts to consider disinvestment<br>– Deliberative process<br>– Documentation and dissemination
**6. Implementation**	▪ Purchasers<br>▪ Requirements for purchasing	▪ Methods of purchasing
	▪ Policy and guidance developers<br>▪ Requirements for policies and guidance documents	▪ Methods of policy and guidance development
	▪ Implementers<br>▪ Requirements for implementation	▪ Methods of project management<br>▪ Methods of change management
**7. Evaluation**	▪ Evaluators<br>▪ Requirements for evaluation<br>▪ Type and source of data collected	▪ Methods of evaluation
**8. (Reinvestment)**	▪ Requirements for reinvestment/reallocation	▪ Methods of reinvestment/reallocation

*Requirement is used in the sense of performance stipulated in accordance with policies, regulations, standards or similar rules or obligations

Table 4. Strengths, weaknesses, barriers and enablers in decision-making for resource allocation

Reproduced with permission from SHARE Paper 3 [14]

Factors identified in response to a specific question about barriers and enablers are noted in italics

STRENGTHS/ENABLERS	WEAKNESSES/BARRIERS
**External environment**	
**General**<br>▪ *Good relationships with external agencies such as Australian Council of Healthcare Standards, Victorian Department of Human Services (DHS)*<br>▪ *Projects initiated by external organisations such as Australian Quality Council, NSW Therapeutics Advisory Group and Clinical Excellence Commission*	
▪ Legislation, regulations, national and international standards, and professional standards must be followed. This provides clarity and certainty for some decisions.	▪ Some decision-makers are unaware of mandatory requirements.
**International**<br>▪ International bodies and national agencies of other countries provide evidence-based recommendations for use of health technologies, clinical practices, models of care, etc.<br>▪ Systematic reviews and Health Technology Assessments are also available.	▪ Decision-makers are frequently unaware of these resources.<br>▪ Due to lack of time, knowledge and skills decision-makers do not actively seek these resources when making decisions and do not differentiate between high and low quality resources.<br>▪ Cost-effectiveness data is often based on modelling which is perceived not to reflect reality
**National**<br>▪ The Medical Services Advisory Committee and Pharmaceutical Benefits Advisory Committee provide evidence-based recommendations for use of medical and surgical procedures and drugs.	▪ Not all medical and surgical procedures and drugs are covered by these processes.<br>▪ Nursing and allied health practices, models of care and clinical consumables are not covered.
**State**	▪ *DHS requirements and processes are cumbersome*
▪ Guidance for introduction of new health technologies and clinical practices (TCPs) is provided by DHS. This includes reporting requirements.<br>▪ Monash Health has developed tools to implement these processes. DHS has recommended these tools to other health services.<br>▪ Monash Health Decision Summaries are published on the health service website.	▪ There is no sharing of information or decisions. Individual health services duplicate the process of finding and appraising relevant evidence, developing business cases, etc.<br>▪ DHS declined to coordinate sharing of information through a central database or website.
▪ The Victorian Policy Advisory Committee on Technology (VPACT) has an annual funding round for introduction of new high cost TCPs	▪ There is no long term state-wide strategic planning for equipment purchases<br>▪ Lack of coordination of equipment use and procurement at state level and no communication between health networks.
▪ Some guidance for purchasing is provided through the Victorian Government Purchasing Guidelines, Medical Equipment Asset Management Framework (MEAMF), Targeted Equipment Replacement Program (TERP) and Health Purchasing Victoria (HPV).<br>▪ HPV is responsible for bulk purchasing of pharmaceuticals, clinical equipment and consumables to streamline ordering and reduce costs. If the item required is in the HPV catalogue the specified brand must be purchased from the designated suppliers at the cost and conditions noted.<br>▪ The processes are transparent and accountability is clear.	▪ HPV catalogue only covers 30% of Monash Health consumables<br>▪ Inclusion of items in the HPV catalogue is not always based on a rigorous evidence-based process<br>▪ Safer, more effective or more cost-effective alternatives may not be included in the catalogue<br>▪ HPV does not cover large items so MEAMF and TERP have no benefits from bulk purchasing and hospitals have to negotiate their own arrangements with suppliers<br>▪ Decision-makers do not know which of these multiple systems are relevant to a particular situation<br>▪ Terminology differs between systems and they are difficult to navigate
▪ The Victorian Aids and Equipment Program is administered by Monash Health on behalf of the DHS. The application process is standardised based on tight explicit criteria for transparency and accountability.	▪ This is a 'last resort' process after other sources of funding have been exhausted. Clinicians waste valuable time writing funding applications for multiple programs which could be integrated and allocated centrally.
▪ The Department of Treasury is interested in supporting disinvestment initiatives but requires details of savings. If savings or reinvestments can be quantified the department may provide more funding.	▪ It is hard to measure the savings<br>▪ The savings are rarely realised because they are absorbed and used to treat more patients

**Monash Health environment: General**	
▪ *Enthusiastic and dedicated staff*<br>▪ *Staff commitment to quality improvement*<br>▪ *Organisational support*<br>▪ *Support from the Executive Management Team*<br>▪ *Support from Directors of Nursing*<br>▪ *Involvement of people who are outside of, or uninterested in, the politics of the organisation*	▪ *High staff turnover in the organisation, particularly agency nurses and junior staff, increases difficulty in communication and implementation*<br>▪ *High staff turnover in projects diminishes organisational knowledge and expertise and increases training requirements*<br>▪ *Organisational culture is difficult to change*<br>▪ *Organisational politics*<br>▪ *Incident reporting software (Riskman) is flawed, does not cover all requirements and does not enable valid aggregation of data related to consumer information*
▪ Strategic planning provides an opportunity for integrating disinvestment decisions into organisational practices. Monash Health had transparent strategic and business planning processes	▪ Lack of strategic planning for large equipment purchases
▪ The Board, Executive Management Team (EMT) and Senior Managers have expressed 'patient-centred care' as a priority.	▪ Considerable pressures on the health service to reduce costs.<br>▪ Perceived distinction between 'what the hospital is concerned about (finances, organisational capacity and risk management) and what the clinician is concerned about (patients)'.
**Monash Health environment: Governance**	
**Oversight**<br>▪ Overall accountability sat with the Monash Health Board. The Board and EMT determined the decision-making structures within the organisation.<br>▪ The Quality Unit maintained an organisational chart of committees related to quality and safety.<br>▪ The Board Secretary also had a list of some committees	▪ No central resource for oversight, coordination or provision of information about committee processes<br>▪ No complete list of committees operating at an organisation-wide level<br>▪ No lists of committees operating within programs or sites
**Policies and procedures**<br>▪ *Robust policies and guidelines for purchasing*<br>▪ *Relevant Terms of Reference for committees*	
▪ Nature and scope of decisions was stipulated in the Purchasing Policy, Purchasing Policy Guidelines and Authority Delegation Schedule to prevent gaps, overlap and ambiguity.	▪ Confusion about 'who does what'<br>▪ Duplication of some committee and project activities
▪ In addition to policies and guidelines there were supporting documents such as application forms, business case templates, requisition forms and checklists governing activities related to resource allocation such as purchasing and procurement and development of clinical guidance documents.	▪ *Too much paperwork and existing paperwork is confusing and ambiguous*<br>▪ Some documents were not well organised, not easily accessible, multiple versions were available and some required considerable skills and resources to complete<br>▪ Emphasis on 'business' aspects and less consideration of evidence of safety, effectiveness and cost-effectiveness in many of these documents
**Transparency and accountability**<br>▪ Transparency and accountability in decision-making was highly valued by respondents<br>▪ Improved transparency and accountability at Monash Health was desired by most respondents	▪ *Lack of transparency in all aspects*<br>▪ *Lack of transparency and accountability in decision-making reduces confidence*<br>▪ Inadequate transparency and accountability was one of the strongest messages from respondents
▪ Clear documented lines of accountability and reporting requirements in some areas<br>▪ Individuals and members of committees at the top of their respective decision-making hierarchies reported that they had clear understanding of how the processes should work, who is accountable, who makes the decision, etc and knew the difference between recommendations, decisions and authorisation.<br>▪ Many of these respondents also reported that all decision-makers have the same understanding as they do.	▪ Many individual and group decision-makers lower down the respective hierarchies admitted they were unsure of the processes. Others who said they were sure gave answers that were inconsistent with each other. Some reported ambiguities and inconsistencies in the systems and processes.<br>▪ Confusion between the concepts of 'decision' and 'recommendation' which may lead to uncertainty in accountability. Some committees saw their role as 'recommending' a course of action with the 'decision' being made by a higher level committee. In contrast, the higher level committees saw their role as one of guidance and support in response to robust investigation of decision options which they expected to occur at the lower level 'decision-making' committees.<br>▪ Individual decision-makers did not always know who to report a decision to and whether formal authorisation was required.

**Conflict of interest**<br>▪ Conflict of Interest required as a standing item on the agendas of relevant committees. Ten of 13 committees interviewed had a process for conflict of interest for committee members, and two of the four committees with an application process had a similar procedure for applicants.	▪ Only one committee, the Technology/Clinical Practice Committee (TCPC), considered the effect of conflict of interest in the provision of evidence used in decision-making
**Monitoring, evaluation and improvement of systems and processes**<br>▪ Quality improvement of systems and processes was supported by respondents<br>▪ Only one committee (TCPC) had an ongoing process of monitoring, evaluation and improvement of its systems and processes, however some committees had undergone a single evaluation/review and some were developing or planning to develop quality improvement processes.	▪ No formal requirements for quality improvement of decision-making at Monash Health<br>▪ At the program level it was noted that 'since there was no formal decision-making process there was no process of review'.
▪ Committees that authorise or support decisions made by other committees expected that a rigorous process of decision-making and prioritisation had occurred	▪ No system to check or regulate this
**Reporting**<br>▪ Quality Unit chart of committees related to quality and safety included lines of reporting<br>▪ Most committees had reporting requirements included in their Terms of Reference	▪ The structure and process of reporting varied with site, department/unit and health professional group making the decisions across and between sites, programs, units, etc difficult<br>▪ No systematic or documented process for reporting of projects
**Monash Health environment: Administration**	
**Relationships, coordination, collaboration and communication**<br>▪ *Knowing who to go to for information*<br>▪ *Knowing who to go to for support*<br>▪ *Networks within the organisation, particularly nursing*<br>▪ *Quality and Risk Managers are good at sharing information across the organisation*<br>▪ *Good communication at site level (nursing)*<br>▪ *Robust and regular communication*	▪ *Lack of knowledge and awareness about*<br>▪ *decision-making systems and processes and where to go to find out about them*<br>▪ *information sources and tools and where to go to find them*<br>▪ *Lack of information regarding how the system works and what processes need to be followed*<br>▪ *Lack of central resource/identified role to provide information about committees*<br>▪ *Lack of organisational processes for knowledge transfer*<br>▪ *Lack of coordination and collaboration between decision-making individuals and groups*<br>▪ *Lack of communication about decisions between programs, departments and other stakeholders*<br>▪ *Lack of communication about impending decisions and projects to enable stakeholder input*
▪ Quality Unit chart of committees included relationships (but only for reporting purposes).<br>▪ Some committees recognised the overlap in their work and the potential to work together. These were in two groups, those considering introduction of new TCPs and those involved in purchasing.<br>▪ People who were members of more than one committee often provided the links between them.<br>▪ There were many examples of cross-unit/department consultation and collaboration for policy and protocol development and implementation.<br>▪ Four projects were linked to others with similar aims	▪ Lack of awareness of other committees within Monash Health<br>▪ Other than reporting, there were no documented relationships between committees<br>▪ Other than the committees considering new TCPs, there were no formal processes of referral for issues that might affect, or should be addressed by, other committees<br>▪ Decision-making 'in isolation' was noted to be a problem in multiple settings. 'Fragmentation' and a 'silo mentality' were used in relation to decisions made without consideration of the areas they will impact upon or consultation with relevant stakeholders.<br>▪ No systematic processes to link projects across the organisation
**Monash Health environment: Stakeholder engagement**	
▪ *Involvement of broad range of stakeholders from multiple sites and a range of health professional disciplines*<br>▪ Reported benefits of broad stakeholder involvement in decision-making included improved decision-making, more effective dissemination of decisions and informing and encouraging others about the need to consult with the groups represented	▪ *Lack of consultation with clinicians in decisions made by managers*<br>▪ *Lack of consideration of impact of change on others when making decisions or planning projects*<br>▪ *Lack of consideration of downstream or lateral impacts eg 'cost saving measures in one area can result in increased costs in another area'*<br>▪ *Limited input from the Quality and the Education Units*
▪ Many respondents supported increased consumer participation and were planning to act upon this	▪ Only one committee (TCPC) included consumer representation in decision-making.<br>▪ Several respondents thought that consumer representation on their committees would be inappropriate or that consumers had insufficient technical understanding to participate.

Monash Health environment: Resources	
**Funding and staff time**<br>▪ *Provision of extra staff*<br>▪ *Availability of extra funds enhanced implementation and evaluation, eg introduction of the National Inpatients Medication Chart had external funding specifically for implementation and evaluation*<br>▪ *Some clinical pathways involve no additional costs*	▪ *Lack of/inadequate funding resulted in*<br>· *lack of/inadequate administration*<br>· *lack of/inadequate evaluation and research*<br>· *compromised building cost estimates, hindering capacity to house/operate equipment properly*<br>▪ *Funding for new equipment frequently did not include funding for training staff to use it or the consumables required.*<br>▪ *Lack of information about available funding*<br>▪ *Staff dissatisfaction with the expectation of their superiors that they will do more work within existing resources*<br>▪ *Insufficient allocation of staff time impairs*<br>· *research and preparation for decisions*<br>· *implementation and evaluation of decisions*<br>· *project delivery*<br>· *training*<br>▪ *Lack of/inadequate coordination of current resources*
▪ Some committees had a Secretariat comprised of 1-2 officers from named roles within the organisation. These positions were allocated sufficient time to complete the required tasks.<br>▪ Some projects were provided with adequate resources for implementation and evaluation<br>▪ Some wards had additional staffing for education support and clinical nurse support. These were invaluable resources for practice change, protocol development and implementation.<br>▪ Some projects had external funding from DHS, universities, etc for staff or infrastructure costs	▪ Some committees used the Personal Assistant of the committee Chair in an administrative role. If a new Chair did not have a personal assistant there would be no resources to support the committee.<br>▪ Some respondents found it difficult to separate the role of the committee from the role of their department. Committee work significantly increased their overall workload, particularly administrative matters, and it was not always clear if these duties were part of, or additional to, their normal duties and what they could cut back in order to accommodate committee obligations.<br>▪ Many projects were to be carried out 'within existing resources'. Respondents noted that they either did unpaid overtime or aspects of the project were not undertaken.
**Expertise and Training**	▪ *Lack of/inadequate skills in*<br>· *use of information technology*<br>· *finding and appraising evidence from research and data*<br>· *project management*<br>· *change management*
▪ Staff in the Centre for Clinical Effectiveness (CCE) and Clinical Information Management (CIM) were available to decision-makers to provide expertise in research evidence and local data respectively.<br>▪ CCE ran training programs in finding and using evidence, implementation and evaluation<br>▪ Six of 10 projects had training for project staff in change management, leadership or IT skills.	▪ CCE's funding for training was redirected due to budget cuts so it was unable to provide free in-house programs (however many staff attended the fee-paying courses CCE provided)<br>▪ Lack of understanding of information systems and project management in senior decision-makers was reported and training for committee members was suggested<br>▪ Most projects used a staff member from the department involved to deliver the project; most of these did not have project skills or expertise.<br>▪ Education and training is not well provided for part-time and night staff
**Information**<br>▪ *Provision of extra computers*	▪ *Lack of computers and/or access to computers, particularly for nurses*<br>▪ *Difficulties using intranet to find organisational data*
▪ CCE and CIM were available to provide information to decision-makers<br>▪ Monash Health libraries provided access to health databases and electronic journals, as well as advice in searching the health literature	▪ *Lack of research evidence and local data to inform decisions*<br>▪ Many decision-makers chose not to use these sources of information<br>▪ Priority was given to senior decision-makers and high level decisions; sometimes decisions at lower levels could not be provided with information due to limited resources

**Decision-makers**	
▪ *Broad committee membership*<br>▪ *Dedication of committee members*<br>▪ *Depth and range of experience of committee members*<br>▪ *Proactive clinicians who think about improving and moving forward*<br>▪ *High level of skill within medical staff acting as leaders in their specialties*	▪ *Clinical autonomy*<br>▪ *High workload in running a committee with lack of administrative staff*<br>▪ *Difficulty taking off 'clinician hat' and replacing it with 'manager or decision-maker hat'*
▪ Committee membership included a range of relevant stakeholders (except consumers) invited to participate because of their role in the organisation or their knowledge and skills in relevant areas.	▪ *Some clinicians feel that if they are experts in a particular area they should not have to justify operational decisions*
**Potential adopters**	
▪ *Having the appropriate profession engaging others in change process, for example nurses should be implementing projects with nurses, not pharmacists.*	▪ *Resistance to change*<br>▪ *Staff cynicism about the importance of changes and relevance to them*<br>▪ *Some clinicians insist on autonomy in their areas of expertise*
**Decision-making process**	
**Identification of need/application**<br>▪ Decisions were instigated by 'top down' direction and 'bottom up' invitation.	▪ General perceptions that<br>▪ financial drivers were stronger than clinical drivers<br>▪ impetus for change was ad hoc, there was no systematic or proactive approach<br>▪ internal bureaucracy and red tape stifled ideas
▪ Some committees had a well-documented application process.	▪ *Complex and time consuming nature of application processes*<br>▪ *People by-pass the system, usually not deliberate but due to lack of awareness of the process*<br>▪ Some applications are driven by pharmaceutical or equipment manufacturers
**Decision criteria**<br>▪ Documenting explicit criteria was generally viewed positively.<br>▪ The committees with application forms had some documentation of criteria.<br>▪ Other decision-making groups and individuals had 'mental checklists' of criteria they considered.	▪ Only one committee (TCPC) and one individual used explicit, documented decision-making criteria.<br>▪ Some committees had no decision-making criteria.<br>▪ Some individual decision-makers strongly rejected documentation of explicit criteria as 'another form of paperwork that will waste clinician's time'.
▪ Most committees considered the Monash Health Strategic Plan, quality, safety, access and equity.<br>▪ All committees considered financial factors.	▪ *Organisational priorities dominated eg*<br>▪ *'Sound practice is not always affordable practice'*<br>▪ *'The operational aspects of nursing (Key performance indicators that are reported to DHS) come first and professional aspects comes second'*<br>▪ There was a perception that there was 'too much emphasis on financial return for investment'
**Ascertainment and use of evidence**<br>▪ *Strong knowledge of the literature*<br>▪ *Attendance at conferences*<br>▪ Using research evidence and local data in decision making was considered to be important.<br>▪ All respondents reported using research evidence and data in decision-making to some extent.<br>▪ Most committees sought a broad membership in order to utilise expertise in the consideration of research evidence and for decision-making with limited evidence.<br>▪ Four out of ten projects sought research evidence from the literature to inform the project.	▪ *Amount of time needed to search the literature or collect data*<br>▪ *Access to evidence is not easy or coordinated*<br>▪ *Lag time between what universities teach and latest research evidence so new staff are not always aware of best practice*<br>▪ *Drug company marketing*<br>▪ Only one committee (TCPC) required explicit inclusion of research and local data and considered the quality and applicability of this evidence. Only one of the projects appraised the evidence used.<br>▪ The other committees had no process to seek evidence from research. When evidence from research and data was used it was not usually appraised for quality or applicability.<br>▪ Due to difficulty finding uninterrupted blocks of time, slow computers and lack of skills in finding and analysing evidence, decision-makers relied on clinical expertise and advice from colleagues.<br>▪ Appropriate local data was frequently reported to be lacking, unavailable and 'manipulated'.

**Reminders and prompts to consider disinvestment**<br>▪ One application form (TCPC) had an explicit question about what the new technology will replace and what can be disinvested.	▪ 'It's all very well to ask the question but it's very hard to get a clinician to say they will stop doing something'.
**Deliberative process**<br>▪ *Robust and honest conversations*<br>▪ *Autonomous decision-making*<br>▪ Decision-makers expressed a desire for a documented standard process.<br>▪ Many respondents noted that the main goal of discussion was to reach decisions by consensus.	▪ *Process not seen as priority for some*<br>▪ *Some committee members do not attend*<br>▪ *Meetings too short for proper deliberation*<br>▪ *Some decisions made reactively, 'on the run', due to lack of consultation or not following process*<br>▪ *Long lag time between application and decision*<br>▪ Lack of standardised process<br>▪ Many of the current processes were perceived to be unclear, 'ad hoc' and lacking objectivity<br>▪ Lobbying, both covert 'behind the scenes' and overt 'squeaky wheels', was perceived to result in favourable decisions.
▪ Most committees required not only the presence of a quorum to make decisions but also attendance of members with relevant knowledge or expertise to the decision at hand	▪ Not all committees had a defined quorum. Of those that did, some made decisions in the absence of a quorum and some made decisions even if a meeting was cancelled due to lack of a quorum<br>▪ Some decisions were made outside committee meetings or by the Chair only
**Documentation and dissemination**<br>▪ One committee (TCPC) published Decision Summaries which were formally distributed to the Therapeutics Committee, EMT, DHS, the Applicant, Department Head and Program Head and made publicly available on the internet.<br>▪ Most committees recorded minutes; these were considered to be confidential and were not published, but were available to appropriate requestors by contacting the committee secretariat<br>▪ All of the individual decision-makers interviewed reported disseminating decisions to people they considered appropriate and, when deemed necessary, disseminating decisions organisation-wide.<br>▪ Many respondents reported others disseminating decisions to them.	▪ *Large size, nature and diversity of the organisation increases*<br>▪ *difficulty in dissemination of information*<br>▪ *frequency and range of communication methods required*<br>▪ *Not everyone uses email*<br>▪ *Using email too often dilutes the effect*<br>▪ The majority of committees did not publish minutes or anything similar.<br>▪ One committee did not keep any records.<br>▪ Although some related committees exchanged minutes there was a lack of formal communication across committees.<br>▪ Documentation and dissemination of decisions made by individuals was informal and ad hoc.<br>▪ Not all projects communicated decisions to other staff members or the wider organisation. Unless people were directly involved, some projects appeared not to make project work or associated decisions public knowledge.<br>▪ Lack of processes for knowledge transfer, especially across sites.
**Implementation**	
**Purchasing**<br>▪ Robust organisational processes that met annual audit requirements<br>▪ Electronic ordering was controlled through an approval hierarchy with delegation thresholds.<br>▪ It was assumed that the decision to purchase was made with due process before reaching the purchasing unit.	▪ Use of evidence in purchasing decisions was not outlined in the Purchasing Policy Guidelines.<br>▪ Those making the decision of 'whether to buy' were responsible for ascertaining evidence of safety, effectiveness and cost-effectiveness in the first stage; however there was no system to check that this has been done before the second stage.
▪ Health Technology Services, the Product Evaluation Committee and working parties set up to evaluate large individual capital purchases considered appropriateness of equipment to Monash Health, availability of spare parts, life expectancy, servicing requirements, related consumables, availability of technical expertise and fit with the DHS Asset Management Framework. They also had expertise in contract negotiation.	▪ *Difficulty managing expectations eg 'once something is approved people want it immediately'*<br>▪ Some were unaware of this process and went directly to the manufacturer. If this was overseas it may be difficult or expensive to get parts, there may not be relevant skills for local maintenance and it excludes benefits that may already exist with a local manufacturer that could supply the same product under better terms and conditions. Re-negotiating contracts, or establishing new ones, creates bad feeling and wastes lots of time.
▪ Purchasing of clinical consumables within budget allocation is done electronically. Electronic authorisation is required for items above individual limits (eg Nurse Unit Manager approval up to $10,000, items above this require authorisation)	▪ There is little assessment of safety, effectiveness or cost-effectiveness of clinical consumable items

**Policy and guidance**<br>▪ Monash Health was developing a new Policy and Procedure Framework<br>▪ Broad support for increased standardisation of practice through policies and procedures<br>▪ Development process seen as a communication tool between professional groups and across sites	▪ Lack of structure and standardisation of processes, especially between sites
**Implementers**<br>▪ *Finding others who have done the same work for support, advice and information*<br>▪ *Establishing Working Parties and Steering Committees for support, endorsement, troubleshooting*<br>▪ *Project leader whose primary role is 'at the coal face'*<br>▪ Decisions made at program level that involve multiple wards, departments or sites are usually implemented by multidisciplinary teams	▪ Some project staff felt isolated and would have liked support from others who had done the same or similar work<br>▪ It was not always clear who was responsible for project management<br>▪ *Lack of/inadequate project management and communication resulted in multiple people*<br>· *making inconsistent changes*<br>· *contacting equipment vendors with requests and ideas for change*
**Practice change**<br>▪ *At site level there is good 'buy-in' for change and people are keen to make things work (nursing)*<br>▪ *Allowing wards to nominate themselves for participation in projects*<br>▪ *'Bottom up' approach to develop individual implementation plan in each ward*<br>▪ *'Bottom up' training to gain staff 'buy in' combined with 'top down' supportive strategy*<br>▪ *Flexible and adaptable staff*<br>▪ *Lots of preparation including training and communication with all stakeholders*<br>▪ *Use of pre-existing (and pre-tested) tools from other organisations*	▪ *Unrealistic project timelines*<br>▪ *Variability in current practice and lack of standardisation increases number of practices to change*<br>▪ *Large size, nature and diversity of the organisation increases complexity of implementation across departments with different needs*<br>▪ *Lack of effective implementation pathways*<br>▪ *Things take a long time to implement, to the point that they 'fall off the agenda'*<br>▪ *Staffing issues, including leave, mean that a lot of projects are on hold*<br>▪ *Project-specific barriers such as logistical challenges with product being implemented*
▪ Some committees provide an approval process only and the applicant is responsible for implementing the decision. In most cases the applicant has control over the process (eg head of department implementing a new procedure) and is motivated to implement the change	▪ Sometimes practice change is required beyond the applicant and their department. Committees do not require applicants to have or acquire knowledge and skills in implementation.
▪ Training and education activities and 'champions' were reported as the two key strategies used to effect change and encourage sustainability of the intervention.<br>▪ Most projects had a champion and/or Executive sponsor. Project champions were generally the head of the relevant department; others included the Chief Executive Officer, Executive Directors who were Steering Committee Chairs and 'Ward Champions' selected to encourage and promote change.<br>▪ Those with champions unanimously considered champions important to the success of the project.<br>▪ Training or education included passive methods using posters and memos, interactive learning on new equipment and participatory approaches involving staff in design and implementation.<br>▪ Seven projects involved training for the target group, most of which was done by external providers of new equipment.	▪ Lack of knowledge and skills in project management, change management and use of information technology were exacerbated when interventions were complex and required high levels of training<br>▪ Lack of known, standardised processes for implementation at Monash Health
▪ Most considered their project sustainable and believed the change was embedded in the system. This was reportedly achieved by involving a variety of staff and 'bottom-up' approaches to change.	▪ Only two considered sustainability in the design of the project.
▪ Half of the projects tailored the implementation plan to anticipated barriers and enablers sourced from other health services, literature searches and personal experiences of project staff.<br>▪ Half reported that implementation was conducted as planned. Some noted that it mostly went to plan but 'amendments were made continually to improve the process'.	▪ One project had no implementation plan<br>▪ Half of the projects did not consider barriers and enablers
▪ *The benefit of the proposed practice change is clear and observable*	▪ Lack of baseline data meant that potential adopters were unable to see the benefit or relevance to their situation resulting in less 'buy in' and poor uptake.

Evaluation of outcomes of decisions	
**General**<br>▪ *Use of pre-existing (and pre-tested) tools from other organisations eg audit tools*<br>▪ Evaluation and monitoring were considered important and had broad support<br>▪ Monitoring of projects after implementation was thought to increase sustainability	▪ *Quality and Risk Managers are not included at the beginning to help with collection of baseline data and evaluation design*<br>▪ Lack of baseline data<br>▪ A lack of data was seen to contribute to the current state of 'little or no process of evaluation'.<br>▪ Limited funds, knowledge and/or skills inhibited both the planning and conduct of evaluation.
**Evaluators**<br>▪ CCE was establishing an in-house Evaluation Service at the time of these interviews.	▪ No specified evaluators with appropriate training or expertise had been utilised by the respondents
**Requirements for evaluation**<br>▪ Monitoring, evaluation and reporting of outcomes was required by DHS sponsored projects and TCPC. The Therapeutics Committee requested reports for some decisions.<br>▪ Routine clinical audits and monitoring of adverse events undertaken for hospital accreditation purposes provided indirect evaluation of decisions in some situations.<br>▪ Half of the completed projects had been evaluated; all but one project reported achieving its planned objectives.	▪ Monash Health had no requirements for evaluation of outcomes of decisions or projects.<br>▪ Most committees had no planned evaluation of outcomes of decisions or implementation projects.<br>▪ The purpose of reports for TCPC and Therapeutics was questioned by some respondents who noted that it may be inconsistent with the knowledge needed for program staff.<br>▪ Only 2 projects planned evaluation as a project component. Some were evaluated post hoc.
**Reinvestment**	
▪ *Reinvestment or reallocation of resources would be an incentive to disinvestment*<br>▪ SHARE Steering Committee keen to establish and support methods for reinvestment/reallocation<br>▪ Flexibility and thinking laterally to include novel methods/indicators such as reducing waiting lists, getting patients out of Emergency Department faster, freeing up time in procedural/operating suites, freeing up bed days that are used to treat another patient group faster (eg X procedure saved y$/bed days which was used by z patients).	▪ *Lack of planning for resource reallocation*<br>▪ *Lack of transparency and consultation in reallocation of savings creates disillusionment*<br>▪ *Staff dissatisfaction that savings generated are not reallocated*<br>▪ A health economist is required to do this properly, Monash Health had no resources for this<br>▪ 'We don't look far enough for downstream effects; we're too simplistic in assessment of savings'.<br>▪ It was noted that savings made in a project in one area sometimes increased costs in other areas; hence reallocation of the savings to the project department would be unfair.<br>▪ Savings of bed days or time in procedural/operating suites were used immediately to treat another patient group so were never realised<br>▪ Accounting practices did not enable measurement and/or reallocation of savings in some areas, for example changes to one TCP may affect multiple cost centres eg department, ward, ICU, pharmacy

Table 5. Examples of criteria for resource allocation decisions
Reproduced with permission from SHARE Paper 3 [14]

WHETHER TO BUY				WHAT, WHERE AND HOW TO BUY		
**Organisation-wide Committee**	**Program Committee**	**Department**	**Individual decision-makers**	**Approved Purchasing Units**	**Organisation-wide Committee**	**Department**
Introduction of new health technologies and clinical practices	Purchase of capital equipment	Purchase of capital equipment	Determination of clinical practices and purchase of clinical equipment	General purchasing	Purchase of clinical consumables	Purchase of pharmaceuticals
Explicit criteria required for decision-making	Criteria 'usually' considered<br>A weighted ranking is used for prioritisation	Theoretical 'ideal' criteria developed in workshop (different to criteria used in current practice)	Criteria 'usually' considered	Criteria 'usually' considered	Criteria 'usually' considered	Criteria 'usually' considered
▪ Conflict of interest (Applicant and Committee members)<br>▪ Evidence of safety, effectiveness and cost-effectiveness (quality of evidence, size of effect and applicability addressed)<br>▪ Cost<br>▪ Clinical feasibility (resource implications, training, credentialing and competency assurance addressed)<br>▪ Access and equity<br>▪ Legal and ethical implications<br>▪ Suitable patient information brochure	▪ Equipment serviceability and impact<br>▪ Clinical risk<br>▪ Occupational Health and Safety risk<br>▪ Accreditation and regulatory requirements<br>▪ Strategic importance to Monash Health<br>▪ Savings in operational cost and/or ability to generate funds<br>▪ Improved access	▪ Workload management<br>▪ Clinical evidence<br>▪ Patient benefit<br>▪ Need<br>▪ Prioritisation of patient groups<br>▪ Waiting list<br>▪ Benchmarking<br>▪ Replacement for obsolescence<br>▪ Staff capacity<br>▪ Allocated budget<br>▪ Ongoing costs<br>▪ Funding opportunities<br>▪ Financial benefit to health service<br>▪ Multi-use of expensive capital<br>▪ State-wide planning and coordination<br>▪ Impact on other areas	▪ Quality and safety/clinical risk<br>▪ Reducing complications<br>▪ Ease of use<br>▪ Staff capacity<br>▪ Cost/cost effectiveness<br>▪ Consumer demand<br>▪ Delivery time of machines<br>▪ Brand changes (implications for spare parts, training, etc)<br>▪ Training needs of staff and consumers<br>▪ Quality of care	All APU purchase decisions are made with commercial/financial consideration including<br>▪ Price<br>▪ Cost-effectiveness<br>▪ Improved supply chain efficiencies<br>Other factors considered<br>▪ Clinical need<br>▪ Legal issues including Health Purchasing Victoria contract requirements	▪ Price<br>▪ Australian standards and regulations for quality and safety<br>▪ Infection control/ Occupational Health and Safety standards<br>▪ Serviceability<br>▪ Business administration such as supply chain and logistics<br>▪ Meets organisation's clinical emphasis and infrastructure requirements<br>▪ Clinical acceptability and effectiveness	▪ Labelling<br>▪ Quality<br>▪ Price<br>▪ Pharmaceutical Benefit Scheme status<br>▪ Acceptance

Table 6. Examples of types and sources of evaluation data used by committees
Reproduced with permission from SHARE Paper 3 [14]

**Process (implementation) and Impact (practice change)**
▪ Progress Reports for new TCPs including number of patients treated, number waiting, new referrals (6 monthly)
▪ Medication safety audits (twice yearly)
▪ Continual Review Evaluation through Australian Council of Healthcare Standards Guide (dates in Nursing Strategic Plan)
▪ Established surveillance mechanisms of transfusion practices (ongoing)
▪ Audits of transfusion practice (random, on behalf of Department of Human Services)
▪ Incident reports (as they arise, documented in Riskman software)
**Practitioner outcomes**
▪ Survey/interview data including user satisfaction and comments (after project implementation)
▪ Clinical practice audits (quarterly)
▪ Incident reports (as they arise, documented in Riskman software)
**Patient outcomes**
▪ Progress Reports for new TCPs including patient outcomes and adverse events (6 monthly)
▪ Reports of adverse events related to new TCPs (at the time of occurrence)
▪ Infection Control surveillance mechanisms (ongoing)
▪ Incident reports (as they arise, documented in Riskman software)
**Economic outcomes**
▪ Clinical Information Management databases of routinely-collected data used to assess
– Cost of falls and falls-related injuries (as required)
– Cost of increased length of stay (as required)
– Costs of products (as required)
– Costs of procedures (as required)
**System outcomes**
▪ Applications for new TCPs including anticipated implications of new TCP on other areas such as intensive care or pharmacy
▪ Reports of 2 year review after introduction of new TCP including actual implications of new TCP on other areas

Table 7. Differences in decision-making between health professional groups

**Decision-making processes** (Reproduced with permission from SHARE Paper 3 [14])
There were notable differences in the decision-making practices of the doctors and nurses interviewed.<br><br>There were more levels of accountability and pathways for operational and clinical support and oversight of nursing decisions compared to medical decisions. Nursing staff reported a hierarchy of decision-making and reporting within the program, the site and the organisation. In the clinical program selected, the Medical Program Director gave the medical department heads sole accountability for their decisions as he considered they were the most senior experts in their specialty areas.<br><br>Nurses reported making more decisions about changing policies and procedures and fewer decisions regarding large equipment purchases; doctors reported the reverse.<br><br>For the individual decision-makers, there was a general feeling among medical interviewees that decisions were made in the best possible way without the use of consistent, explicit, documented criteria and that efforts within the organisation to introduce this encountered resistance. Conversely, some nursing staff welcomed the use of documented criteria for the potential benefits of increasing transparency, standardising practice, decreasing the unintended consequences of some decisions and reducing adverse events.<br><br>While research evidence and local data were valued in decision-making for both groups, nursing staff reported the use of local data more often than medical staff. Medical staff noted the use of research evidence in guiding decisions more often than nurses, and also commented on the shortage of research evidence in many of their specialty areas.
**Information needs** (Reproduced with permission from SHARE Paper 7 [15])
The surveys found that medical staff used systematic reviews and original research, and accessed health databases and the Cochrane Library, more often than nurses. They also had higher levels of confidence for all aspects of finding, appraising and using evidence in decisions. Allied health staff came somewhere between the two for most findings. These findings are consistent with others [16, 17], but also in contrast to the review by Younger (2010) who found no significant differences [18].<br><br>When selecting a preferred format for education in searching for, accessing and appraising evidence medical staff preferred self-paced online tutorials, nurses interactive workshops (eg ½ to 1 day), allied health staff short courses (eg 2-3 days) and the management/support staff had equal preference for lecture series (eg 1 hour per week for 10 weeks) and interactive workshops. The preferred formats for education in implementation of change were slightly different with medical staff still choosing self-paced online tutorials but nursing, allied health and other staff all preferring interactive workshops.

Consumer engagement was acknowledged as integral to the proposed program; however there was a lack of guidance about systematic approaches to identify, capture and incorporate consumer perspectives into resource allocation decision-making, implementation and evaluation [19]. Several consistent messages for consumer engagement relevant to this context emerged from the literature and local research. Two concepts were identified by the consumer participants but were not found in the literature: sources of information regarding consumer values and perspectives in publications and locally-collected data and methods to use them in health service decision-making and the need for mechanisms within health services to receive and act upon consumer-initiated contributions. A model bringing these elements together was developed to integrate consumer values and preferences into organisation-wide decision-making for resource allocation. Definitions of the terms used in the model were included. (Figure 4 and Tables 8-11) [19].

Figure 4. Model for integrating consumer values and preferences into the resource allocation process

Reproduced with permission from SHARE Paper 4 [19]

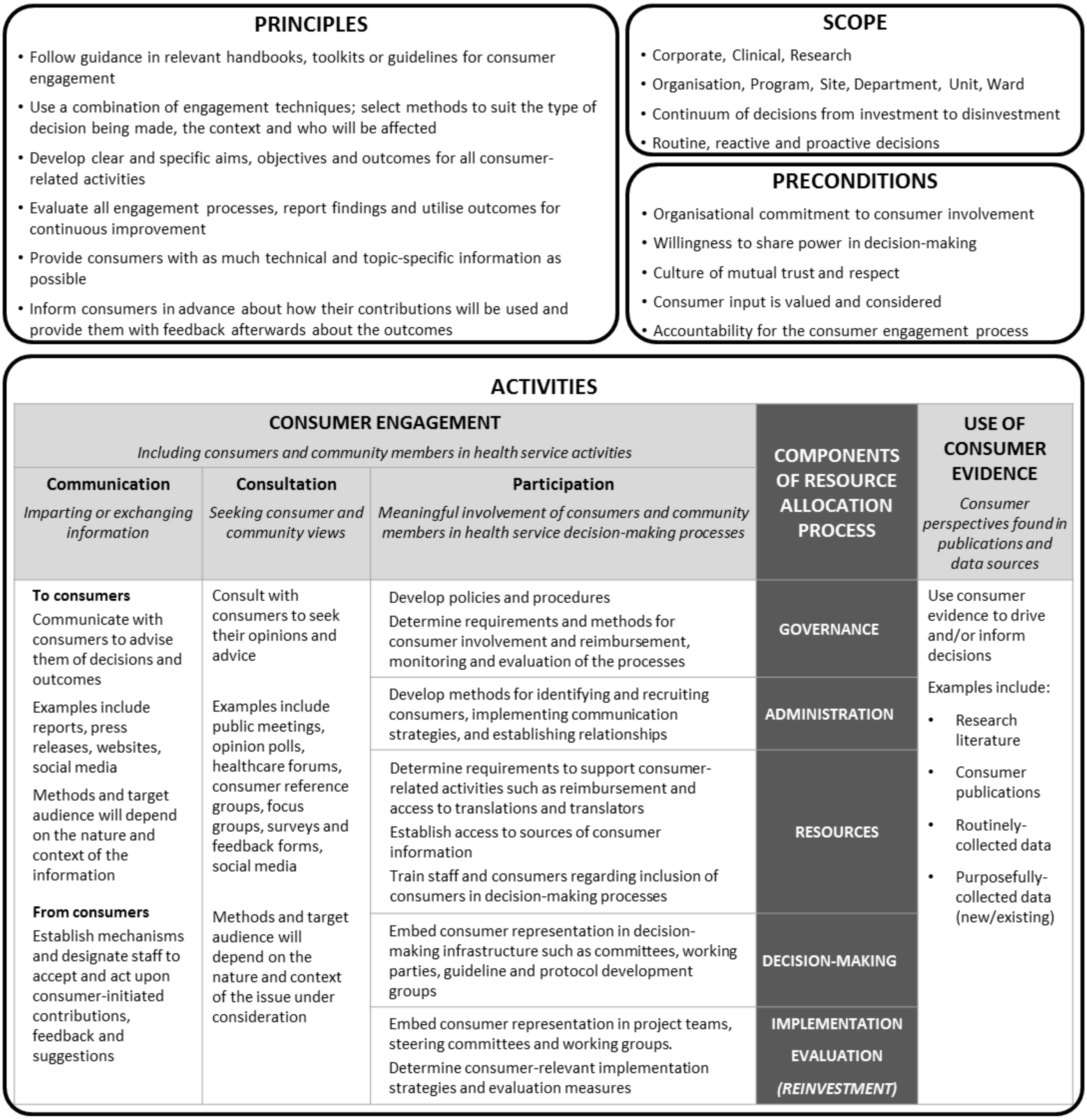

Table 8. Examples of consumer-related activities generating proactive decisions to drive change

Reproduced with permission from SHARE Paper 4 [19]

Research	Regularly scanning published research evidence such as reviews by the Cochrane Consumer and Communication Group or publications from relevant consumer agencies for applicability to the local context and comparing the findings with current practice to determine whether there is a need for change
Data	Actively exploring local sources of routinely-collected data such as complaints registers or patient satisfaction surveys for trends or emerging themes that identify opportunities for improvement
Engagement	(Communication) Establishing mechanisms to encourage, accept and act upon consumer-initiated feedback
	(Consultation) Seeking regular consumer feedback to initiate change in targeted areas, for example:<br>▪ Topics that are important to patients such as pain management and early discharge<br>▪ Topics that are important to the health service such as cost containment of high volume or high cost procedures where consumer priorities may inform selection of suitable alternatives<br>▪ Big problems for patients and health services such as falls and medical mishaps<br>▪ Patients with high usage of health services such as those on renal dialysis<br>▪ Patients interacting with areas of the health service undergoing frequent or significant change<br>▪ Patients with cultural, ethnic or language differences that require additional resources

Table 9. Examples of routinely-collected consumer data

Reproduced with permission from SHARE Paper 4 [19]

**SATISFACTION SURVEYS**
**Victorian Patient Satisfaction Monitor (VPSM)** is a state-wide survey that selects respondents at random; users are sent a unique ID to complete the survey by invitation only.
**The Victorian Healthcare Experience Survey (VHES)** is a state-wide survey that allows a wide range of people to provide feedback on their experiences and features specialised questionnaires for adult and child inpatients and emergency department attendees, including parents/ guardians, and maternity clients. Surveys are distributed in the month following the admission or attendance. People may respond either online or on paper with a freepost return. Surveys are available in English and a range of community languages.
**COMPLAINTS, COMPLIMENTS, COMMENTS**
**Monash Health**<br>Complaints, compliments and comments can be made by completing an online form, mailing a printable version of the form, or in person by phone. Complaints are dealt with by the Consumer Liaison Officer on each campus. Details are kept by the Quality Unit.
**The Office of the Health Services Commissioner (OHSC)**<br>Complainants can also correspond directly with the OHSC.<br>The OHSC's role is to receive, investigate and resolve complaints from users of health services, to support healthcare services in providing quality healthcare and to assist them in resolving complaints. The legislation also requires that information gained from complaints be used to improve the standards of healthcare and prevent breaches of these standards.<br>This information was the subject of the first study of its kind in Australia in 2014 leading to recommendations for change. The report is available at http://docs.health.vic.gov.au/docs/doc/Study-of-people-lodging-a-complaint-with-the-Victorian-Health-Services-Commissioner
**OTHER**
Individual health services and state health departments conduct service reviews, audits and other studies that include patient and consumer information

Table 10. Examples of publications with consumer information

Reproduced with permission from SHARE Paper 4 [19]

**CONSUMER HEALTH JOURNALS**
**Health Voices – Journal of the Consumers Health Forum of Australia** is published two times a year to promote debate on health care issues affecting all Australians and of interest to health consumers, government and industry. https://www.chf.org.au/health-voices.php
**The Australian Health Consumer** was the official journal of the Consumers Health Forum of Australia from 2001 to 2007. It provided a consumer-focused appraisal of the current and ongoing major health issues of the day in the state, national and international health sector. https://www.chf.org.au/australian-health-consumer.php
**The Patient: Patient-Centered Outcomes Research** is the only journal that aims exclusively to examine the needs, values and role of the patient in an increasingly complex healthcare landscape in which funding and decision-making require ever-greater awareness of the patient's perspective. The journal deals with the full range of patient-centered studies, reviews and commentary ranging through techniques such as conjoint analysis, patient reported outcomes, studies on compliance and satisfaction through to patient-directed health plans and patient literacy. http://www.springer.com/adis/journal/40271
**Patient Intelligence** is an international, peer reviewed, open access journal that characterizes and measures the central role of patient behavior and intention in optimizing healthcare management in all areas of disease and complaint types. An improved understanding of patient intelligence coupled with predictive analysis helps an organization contribute more effectively to achieving better outcomes. The journal is characterized by the rapid reporting of reviews, original research, methodologies, analytics, modeling, clinical studies and patient surveys across all disease areas. Specific topics covered in the journal include: Patient and healthcare literacy, Patient information and healthcare professional communication/interaction, Patient behavior, attitude and trends, Behavior management programs, Quantitative and qualitative research, Data collection systems Business performance management, Benchmarking, assessment and reporting systems, Patient preference, satisfaction, convenience, acceptability and adherence, Patient involvement in the design and development of new treatments and management protocols to optimize outcomes, Decision support systems incorporating patient intelligence.<br>http://www.dovepress.com/aims-and-scope-patient-intelligence-d203-j90
**Patient Preference and Adherence** is an international, peer reviewed, open access journal that focuses on the growing importance of patient preference and adherence throughout the therapeutic continuum. The journal is characterized by the rapid reporting of reviews, original research, modeling and clinical studies across all therapeutic areas. Patient satisfaction, acceptability, quality of life, compliance, persistence and their role in developing new therapeutic modalities and compounds to optimize clinical outcomes for existing disease states are major areas of interest for the journal. http://www.dovepress.com/aims-and-scope-patient-preference-and-adherence-d16-j20
**Patient Related Outcome Measures** is an international, peer-reviewed, open access journal focusing on treatment outcomes specifically relevant to patients. All aspects of patient care are addressed within the journal and practitioners from all disciplines are invited to submit their work as well as healthcare researchers and patient support groups. Areas covered will include: Quality of life scores, Patient satisfaction audits, Treatment outcomes that focus on the patient, Research into improving patient outcomes, Hypotheses of interventions to improve outcomes, Short communications that illustrate improved outcomes, Case reports or series that show an improved patient experience, Patient journey descriptions or research.<br>http://www.dovepress.com/aims-and-scope-patient-related-outcome-measures-d188-j84
**CONSUMER HEALTH ORGANISATION NEWSLETTERS**
**Consumers Shaping Health** is a bi-monthly newsletter published by the Consumers Forum of Australia (CHF) for members, consumer representatives and stakeholders in health. It promotes current advocacy work of CHF in its three priority areas: safety and quality in health care; health care for people with chronic conditions; and safe and appropriate use of medicines.<br>https://www.chf.org.au/consumers-shaping-health.php
**COCHRANE CONSUMERS AND COMMUNICATION REVIEW GROUP**
The Cochrane Consumers and Communication Review Group is part of the international Cochrane Collaboration. The Group coordinates the preparation and publication of systematic reviews of interventions which affect the way people interact with healthcare professionals, services and researchers. These reviews are published in The Cochrane Library. http://cccrg.cochrane.org/welcome
**QUALITY OF CARE REPORTS**
All Victorian health services are required to publish an annual Quality of Care Report each financial year. The primary audience includes consumers, carers and the health service community. Health services should consult with consumers, carers and community members and/or their Community Advisory Committee about the specific content. Minimum requirements include:<br>▪ Consumer, carer and community participation<br>▪ Quality and safety reporting at least four key measures annually (from preventing and controlling healthcare associated infections, medication safety, preventing falls and harm from falls, preventing and managing pressure injuries, clinical indicators for dental services, safe use of blood and blood products)<br>▪ A review of their local clinical governance policy against the Victorian clinical governance policy framework<br>▪ A report of the health service's response to needs of consumers, families or carers and the community across the continuum of care.<br>▪ Examples or stories that show how these initiatives work in practice.
**OTHER**
**Consumer driven healthcare** is designed to help health care organizations respond effectively to the shift in market power, become consumer-centric, and position themselves to become market leaders in the new consumer-driven market.<br>http://go.galegroup.com.ezproxy.lib.monash.edu.au/ps/i.do?action=interpret&v=2.1&u=monash&it=JIourl&issn=1542-0914&p=AONE&sw=w&authCount=1

Table 11. Definitions of consumer-related terms

Reproduced with permission from SHARE Paper 4 [19]

Health consumers	Patients, potential patients, current and previous users of health services; parents, guardians or carers of patients; organisations representing consumers' interests; members of the public who are targets of health promotion programs (adapted from ACSQHC [20], CHF [21])
Consumer representatives	Members of a committee, steering group, working party, panel or similar decision-making group who voices the consumer perspective and takes part in the process on behalf of consumers (adapted from CHF [21])
Community	Group of people sharing a common interest including cultural, social, political, health and economic interests and/or a geographic association (adapted from CHF [21])
Consumer engagement	Inclusion of consumers and/or community members in a continuum of activities from passive behaviours such as receiving information, through more active participation, to shared decision-making with equal power. These activities include, but are not limited to, provision of information, consultation, development, participation, collaboration and empowerment (adapted from Sarrami-Foroushani et al [22], O'Mara-Eves et al [23])
Communication	Consumers and/or community members are engaged through imparting or exchanging information. Information can be verbal, written or provided by other methods. Communication can go both ways between consumers and/or community members and health service staff
Consultation	Consumers and/or community members are engaged through requests to provide their views, preferences, comments and suggestions to inform the decision-making process, but the consumers and/or community members may not be engaged in subsequent decision-making or action (adapted from PICS [24], CHF [21])
Participation	Consumers and/or community members are engaged through meaningful involvement in decision-making processes for health policy and planning, healthcare management and service delivery, care and treatment, and the wellbeing of themselves and the community (adapted from Victorian Department of Human Services[25], CHF [21])
Consumer evidence	Consumer opinions, perspectives and preferences found in publications and data sources
Routine decisions	Decisions made on a recurring basis or scheduled via a timetable eg annual budget setting processes, six-monthly practice audits, monthly Therapeutics Committee meetings, reviews of protocols at specified intervals after their introduction, etc [14].
Reactive decisions	Decisions made in response to situations as they arise eg new legislation, product alerts and recalls, applications for new drugs to be included in the formulary, critical incidents, emerging problems, etc [14].
Proactive decisions	Decisions driven by information that was actively sought for this purpose eg accessing newly published synthesised research evidence such as Cochrane reviews to compare against current practice, interrogating routinely-collected datasets to ascertain practices with high costs or high rates of adverse events, etc [14].
ACSQHC Australian Council on Safety and Quality in Health Care, CHF Consumer Health Forum, PICS Paediatric Integrated Cancer Service	

Defining the components

Information from the published literature and local research was synthesised to identify the most sustainable, effective and appropriate approach to disinvestment at Monash Health [3]. Multiple factors for consideration in establishment of the new program were extracted (Table 12). These findings led to definition of the program elements: four components, their aims and objectives, relationships between the components, principles that underpin the program and preconditions for success and sustainability. The principles were agreed upon, the preconditions were established, and implementation and evaluation plans were developed. These findings were incorporated a model for sustainable healthcare through allocation of sustainability in health care by allocating resources effectively (SHARE) in the local healthcare setting (Figure 5) [3].

The initial SHARE proposal had two aims, to develop systems and processes for decision-making and to undertake disinvestment projects. The systems and processes would lead to identification of target TCPs to be disinvested in individual projects. This sequential process is represented by an arrow from Aim 1 to Aim 2.

Based on information from the literature and stakeholder feedback it was clear that these two aims would not be successful without provision of expertise and support to facilitate decision-making (systems and processes) and implementation of change (projects). These needs are represented by arrows from Aim 3 to Aims 1 and 2.

Detailed program evaluation and research to measure and understand the change process were considered to be a vital fourth component and would be applied to the other three components. The double headed arrows between Aim 4 and Aims 1, 2 and 3 indicate that evaluation and research inform further development of the components which in turn would be evaluated and researched. The Principles and Preconditions sit above and below the four aims indicating that they apply to the whole program.

Table 12. Factors that influenced decisions for SHARE program development

Reproduced with permission from SHARE Paper 5 [3]

Finding	Source	Decision	Program element
Potential benefits of disinvestment identified	Literature	Establish a program exploring disinvestment at Monash Health.	SHARE program
External environment supportive of disinvestment program	Literature & DHS docs		
Internal environment supportive of disinvestment program	Monash Health Staff		
Capacity for leadership in this area demonstrated	New TCP program		
The word 'disinvestment' is associated with negative connotations, high risk of engendering suspicion and distrust and getting stakeholders offside.	Literature<br>Monash Health Staff	Proceed carefully, avoid the term 'disinvestment' and use positive language.	Principles
'Top down' approach seen as negative. Needs to be balanced with 'bottom up' strategies and involvement of stakeholders.	Literature<br>Monash Health Staff	Implement 'top down' and 'bottom up' strategies, make stakeholder engagement a priority, integrate methods for staff to drive change into the new systems and processes.	Principles<br>Preconditions
A systematic integrated approach would be better than ad hoc decisions, individuals 'championing' causes or projects undertaken in isolation.	SHARE leaders<br>International experts	Focus on organisation-wide approach to decision-making that integrates new and current systems and processes.	Principles
Perceived lack of transparency and accountability and suboptimal use of evidence in current decision-making processes. Power struggles and hidden agendas perceived to influence outcomes.	Monash Health Staff<br>Project team	Ensure the new systems and processes are transparent, accountable and evidence-based.<br>Introduce explicit criteria for disinvestment decisions.	Principles
Lack of transparency and accountability in reallocation of funding released through disinvestment would be significant barrier to effective program.			
Lack of consistent terminology, absence of decision-making criteria and no guidance to inform an organisational approach.	Literature<br>International experts	Develop our own frameworks and methods.	Principles
Disinvestment should not be considered in isolation but alongside other decisions. Investment and disinvestment decisions are often linked, disinvestment occurs when something new is introduced.	Monash Health Staff<br>SHARE leaders<br>Project team	Do not focus on 'disinvestment' or 'investment' alone. Consider 'resource allocation'. Establish processes along decision-making continuum from introduction to removal.	Principles
Health service staff perceive management priorities to be focused on saving money. The concepts around 'disinvestment' accentuate this.	Literature<br>Monash Health Staff	Focus on 'effective application of health resources' to facilitate a positive approach.	Principles
The program needs a strong positive image that reflects the new focus on 'effective application of health resources'. Being compatible with 'iCARE', the familiar acronym for Monash Health values would be beneficial.	Monash Health Staff<br>SHARE leaders<br>Project team	Change name from 'Disinvestment Project' to 'SHARE' (Sustainability in Health care by Allocating Resources Effectively)	Name
Six potential opportunities to integrate disinvestment decisions into organisational infrastructure, systems and processes were identified.	Literature<br>SHARE leaders	Investigate methods to implement disinvestment decisions in the six settings identified.	Systems and Processes
Undertaking disinvestment projects was a key element of the original proposal. Waiting for investigation of the six settings is too long to delay pilot projects. Some 'quick wins' would be valuable.	SHARE leaders<br>Monash Health Staff	Develop methods to identify and prioritise potential target TCPs in parallel with the investigation of the six settings. Undertake pilot projects to disinvest them.	Disinvestment projects
Current decisions are made 'routinely' or 'reactively'. Introduction of TCPs is based on applications from clinicians or managers and removal of TCPs is based on emerging problems or product alerts and recalls. Research literature and local data could be used 'proactively' to drive health service practice.	Monash Health Staff<br>SHARE leaders<br>Project team	Build on current 'routine/reactive' processes that are done well.<br>Develop new processes to use evidence 'proactively' to drive decisions and/or priority setting.<br>Make these explicit elements of the new program.	Principles
Using evidence 'proactively' requires time and attention from decision-makers. The information	Monash Health Staff	Develop methods to identify appropriate high-quality information,	Systems and

Finding	Source	Decision	Program element
provided must be trustworthy, applicable and sufficiently important to warrant adding to their workload.	SHARE leaders	process and package it for ease of use and deliver it to the relevant decision-makers.	Processes
Decisions for resource allocation are delegated to committees and individuals. There are opportunities for improvement in the governance of these processes and to introduce routine consideration of 'disinvestment'.	Monash Health Staff<br>SHARE leaders<br>Project team	Review processes and governance of decision-making by committees and the authority delegation schedule	Systems and Processes
There is no guidance on consumer participation in disinvestment activities.	Literature	Develop methods to capture and utilise consumer perspectives and integrate them into the new program.	Systems and Processes
With a few exceptions, committees and project teams do not routinely involve consumers in making or implementing decisions and the organisation does not have a framework for engaging consumers.	Monash Health Staff<br>Project team		
The systems and processes for evidence-based decision-making cannot be delivered without appropriate and adequate skills and support	Literature<br>Monash Health Staff	Develop support services that enable capacity-building and provide expertise and practical assistance	Support Services
With a few exceptions, staff do not routinely seek evidence for decisions, are unaware of best practice in implementation and do not evaluate outcomes.	Monash Health Staff<br>Project team	Provide expertise, training and support in accessing and utilising evidence in decisions.<br>Provide expertise, training and support in implementing and evaluating evidence-based change.	Support Services
The main barriers to use of evidence and effective implementation are lack of time, knowledge, skills and resources.	Literature<br>Monash Health Staff		
Health service projects are not usually well supported. It is common for funding to be insufficient, timelines inadequate and staff lacking in knowledge and skills in project management, data collection and analysis.	Monash Health Staff<br>Project team	Influence planning of disinvestment projects to ensure adequate resources and appropriate timelines.<br>Provide expertise, training and support in project methods and administration	Support Services
Disinvestment projects are generally based on health economic principles	Literature	Utilise in-house expertise and take an 'evidence-driven', rather than 'economics-driven', approach to investigation of disinvestment in the health service context.	Principles
Monash Health does not have expertise in health economics and does not intend to fund this in the foreseeable future	Monash Health Leaders		
Safety, effectiveness, local health service utilisation and benchmarking parameters are possible alternative considerations for disinvestment.	SHARE leaders<br>Monash Health Staff<br>Project team		
Monash Health has high-level expertise in accessing and using research evidence and health service data to inform decisions.			
Monash Health does not have the level of expertise in health program evaluation required for SHARE and has no expertise in health economics.	Project team	Engage consultants in health program evaluation and health economics to assist in development and evaluation	Preconditions
There is no guidance to inform a systematic organisational approach.	Literature	Undertake action research to investigate the process of change in addition to program and economic evaluations.<br>Run a national workshop to learn and share information.<br>Disseminate all findings.	Evaluation and Research
In addition to detailed program and economic evaluation, understanding what happened in the process of investigation, what worked, what didn't work and why is required.	SHARE leaders<br>Project team		
This large program will need funds. It is consistent with the disinvestment agenda of the Victorian DHS who are sympathetic to a funding application.	DHS documents<br>DHS staff	Seek funding from the state health department.	Preconditions
To be successful this ambitious proposal will need endorsement, support and strategic direction from the highest level and links to those with power and influence in the organisation.	Literature<br>SHARE leaders<br>Project team	Increase membership of the Steering Committee to reflect those best able to provide the appropriate influence, direction and support.	Preconditions
All projects should be aligned to the Monash Health Strategic Goals. Program activities will be facilitated if integrated into the organisation Business Plan.	SHARE leaders<br>Project team	Align SHARE with the Monash Health Strategic Goals and include program activities in the annual Business Plans	Principles

Abbreviations DHS: Victorian Department of Human Services, TCP: Technology or clinical practice, iCARE: Integrity, Compassion, Accountability, Respect, Excellence

Figure 5. Model for exploring sustainability in health care by allocating resources effectively in the local setting

Reproduced with permission from SHARE Paper 5 [3]

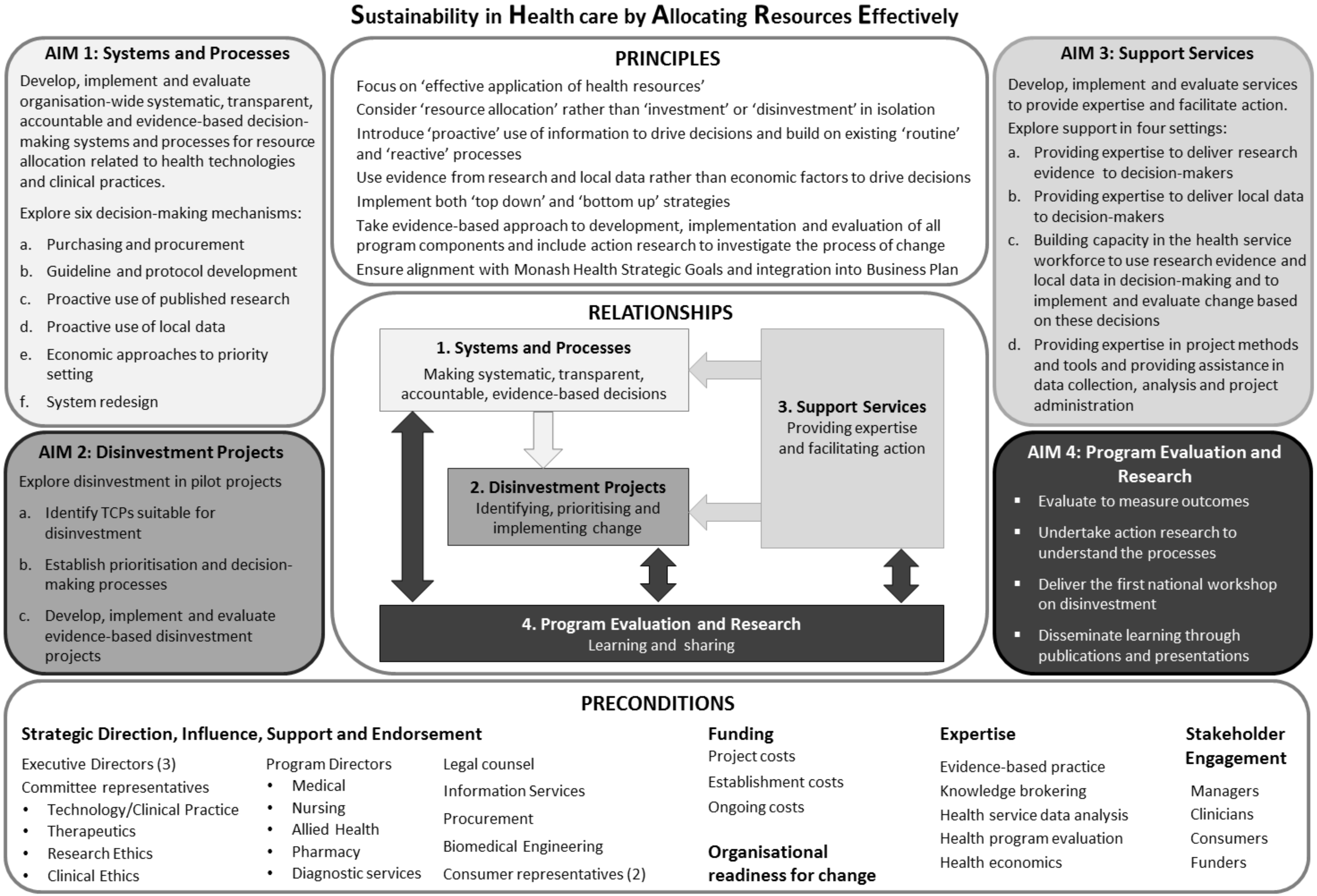

PHASE TWO (SHARE Papers 6-8)

Phase Two of the framework for complex interventions [1] involves a series of exploratory trials assessing acceptability and feasibility of the components and identifying methodological issues for implementation and evaluation. The aims identified in Figure 5 were investigated in Phase Two. The nature of the innovations and methods to deliver them would be explored, those thought to be feasible would be piloted and those found to be sustainable, effective and appropriate would be established as ongoing processes.

Funding was reduced in the final year of the program resulting in limitation of some implementation and evaluation activities due to the shortened timelines.

A summary of the activities in Aims 1 and 2 is provided in Figure 6.

Aim 1. Systems and processes

The focus of Aim 1 was to explore the six decision-making mechanisms with potential to systematically identify opportunities for disinvestment proposed in Figure 1 [2].

Aim 1.1 Purchasing and procurement

Health services have well-established infrastructure for spending money such as purchasing of drugs and clinical consumables and capital procurement for building and equipment. Incorporating prompts, triggers and mandatory requirements to consider disinvestment into these existing systems and processes might be achieved quickly and, once established, delivered with no additional costs [2].

Only one prompt to consider disinvestment was identified in the wide range of decision-making contexts investigated at Monash Health [26]. The SHARE activities resulted in some positive outcomes related to introduction of new TCPs, but no changes regarding identification of opportunities for disinvestment were implemented [27]. This was due to local barriers; in particular that the relevant processes were outside the control of the SHARE team.

The current literature includes discussion about smart, innovative and evidence-based purchasing [28, 29], and the need to consider economic evaluations in purchasing decisions [30], but we were unable to find mention of purchasing or procurement processes being used to identify local disinvestment opportunities.

Aim 1.2 Guideline and protocol development

In addition to processes that allocate funding, health services also have systematic mechanisms for allocating non-monetary resources such as local guidelines and protocols that determine use of drugs and equipment, diagnostic tests, surgical procedures, clinic capacity, etc [2]. There are potential opportunities for disinvestment in all of these activities which could be initiated through prompts, triggers and mandatory requirements in document development, authorisation processes, implementation strategies and evaluation activities.

CCE staff members were simultaneously developing a new Policy and Procedure Framework for Monash Health and included a prompt in the instructions to document developers to consider whether any current practices could be discontinued [31]. CCE handed the new framework over to the department with oversight of organisational documents for implementation and ongoing governance. The disinvestment prompts, along with other instructions, were removed by the implementers with the intention of making the process less onerous for document developers [27].

While many authors refer to the potential to use guidelines for implementation of disinvestment recommendations [13, 32-35] none propose local guideline and protocol development as a method to identify disinvestment opportunities.

Aim 1.3 Proactive use of published research

There is a growing body of evidence about practices that are harmful, of little or no benefit, or where a more effective or cost-effective alternative is available. Searches for evidence-based disinvestment opportunities could be undertaken and the findings delivered directly to decision-makers [2]. Once potential disinvestment opportunities are identified from research, local data could be used to assess the burden of disease, volume of use, likely outcomes and potential cost of change. If an issue only affects a few patients or practitioners, or the burden of disease and hence potential impact are small, particularly in comparison with other issues, resources for change may be better employed elsewhere.

The SHARE team developed a catalogue of 184 potential disinvestment targets from known sources of high quality synthesised evidence [36-40] and evidence-based publications focused on disinvestment [41, 42]. Use of the catalogue to identify disinvestment projects is discussion in Aim 2 below [27]. A broader approach to proactive use of research evidence was piloted as an Evidence Dissemination Service which is discussed in Aim 3 [43].

Figure 6. Overview of activities for SHARE Aims 1 and 2

Reproduced with permission from SHARE Paper 6 [27]

OBJECTIVES	APPROACHES TO BE INVESTIGATED	ACTIVITIES	APPLICATION AT MONASH HEALTH	CONCLUSIONS
To identify potential disinvestment opportunities	Purchasing and procurement	Interviews, Document analysis, Consultation	Not applied, local barriers	Insertion of prompts to consider disinvestment into purchasing processes and documentation has potential
	Guideline and protocol development	Development of new policy and procedure framework	Not applied, local barriers	Insertion of prompts to consider disinvestment into processes and documentation for guideline development has potential
	Proactive use of published research	Database searches, development of catalogue and taxonomy	Not applied: no criteria for use were developed	Catalogue of evidence-based disinvestment opportunities potentially useful if criteria for searching and systematic prompts to trigger use are developed
	Proactive use of local data	Consultation with data experts and quality program staff	Not applied: local barriers	Interrogation of local data potentially useful if criteria for searching and systematic prompts to trigger use are developed
	Economic approaches to priority setting	Literature review<br>Workshop	Not applied: no health economist	PBMA useful for identifying disinvestment opportunities but need a health economist to implement
	System Redesign	Literature review<br>Interviews, Workshop	Not applied: not useful for identification	System redesign not useful for identifying disinvestment opportunities but potentially useful for implementing and/or quantifying disinvestment
	Expressions of interest	Criteria development<br>Application process	2 EOIs received and investigated	EOIs potentially useful if proposals are fully considered, evidence-based, and intervention and objectives are clear
	Additional systematic methods identified	Not investigated	Not applied	Potentially useful, requires investigation
	Non-systematic proposals		17 proposals received and investigated	No evidence of benefit, did not work in SHARE Program
To establish prioritisation and decision-making processes	Prioritisation frameworks	Literature review<br>Framework development	Partial implementation, no evaluation: local barriers	Currently available methods and tools are potentially useful.<br>SHARE problems were unrelated to the frameworks
	Decision-making frameworks	Consultation with key informants	No systematic, process developed: local barriers	Currently available methods and tools are potentially useful.<br>SHARE problems were unrelated to the frameworks
	Non-systematic decisions		4 proposals accepted	No evidence of benefit, did not work in SHARE Program
To develop, implement and evaluate projects	Methods for project development, implementation and evaluation	Literature review<br>Interviews<br>Surveys	1 project commenced<br>SHARE Program ended prior to project completion	Currently available generic methods and tools for planning, project management, implementation and evaluation are potentially useful for disinvestment projects
To measure outcomes and understand processes	Methods and measures to assess determinants of effectiveness, process of change and outcome measures	Adaptation of a theoretical framework and taxonomy for use in disinvestment<br><br>Application of framework to SHARE findings	Assessment of factors influencing SHARE processes	Generally negative: difficulty identifying suitable projects, lack of systematic approach, nominations made by proposers not directly involved, lack of clarity and rationale in proposals, lack of authority to change practice
			Assessment of factors influencing the disinvestment project	Generally positive: good evidence, clear objectives, pathway to be changed was well-documented, external funding provided, detailed data collection, strong local ownership, champions sought training and advice, 'win-win' for staff involved, resources reallocated to same department

The concept of a catalogue of disinvestment opportunities has been discussed widely in the literature under the more recently coined term ‘low value’ lists [44]. Unfortunately, not all the lists are as trustworthy as the high-quality sources noted above. Some are based on expert opinion only, some from a combination of evidence and expert opinion, and some do not specify methods or provide an explicit definition of ‘low value’ [45].

Aim 1.4 Proactive use of local data

Hospitals and other health facilities routinely collect large amounts of data. Monash Health decision-makers often used local data reactively to understand problems or develop solutions, but they did not use it proactively to review current practice, seek opportunities for improvement or drive priority setting [14]. Three approaches to targeted analysis of routinely-collected data to discover opportunities for disinvestment were proposed [2]: to identify areas where disinvestment might have the greatest impact, to investigate variations in practice, and to explore less commonly used data sources such as complaints registers or patient satisfaction surveys. In the same way that local data could be used to substantiate a decision to disinvest arising from research evidence, research evidence would inform a decision arising from local data by identifying best practice in the relevant area and confirming whether change is needed and what the appropriate alternatives are [2].

The first two approaches were to be explored within the Data Service discussed in Aim 3 below [15]. The third approach was to be considered in a consumer engagement framework, however limitations of incident reporting software and consumer information available from other sources prevented exploration at the time [19].

There is a large body of literature on examination of practice variation [46]. Two recent studies have used practice variation in national and regional settings specifically to identify ineffective practices and consider the potential to do so at local health service level [47, 48]. They also note that procedures with high variability are often not on the ‘low value’ lists, indicating additional possibilities to identify disinvestment opportunities from this approach [48]. While local data is potentially valuable in identifying and substantiating need for change, problems with validity, reliability, comprehensiveness and degree of sensitivity to disinvestment requirements remain significant barriers [8, 13, 48-51].

Aim 1.5 Economic approaches to priority setting

Priority setting exercises use economic principles to weigh up options for investment and disinvestment and select preferred alternatives using pre-determined criteria [2].

Four methods met the criteria of economic analysis applicable at the local health service level; however all had limitations in their ability to identify disinvestment opportunities in this context [27]. Program Budgeting and Marginal Analysis (PBMA) is the most widely used method, the process is well-tested and guidance is available [52, 53]. The lack of in-house health economics capability was the key factor in the decision that priority setting exercises were not feasible at Monash Health [27].

Although decision-makers acknowledge the usefulness of PBMA, it remains quite difficult to achieve in practice [49, 50, 54]. The major limitations for all priority setting approaches are lack of standardisation in cost-accounting, lack of sufficient high quality data to inform decision-making, and lack of time and skills to undertake the process and implement the decisions [10, 49-51, 53-55].

Aim 1.6 System redesign

System redesign describes a range of methods and tools to review whole systems of care. It is a familiar process in health services, it offers a well-accepted context to introduce practice change, and it could be integrated into a systematic organisation-wide approach to disinvestment [2].

No examples of system redesign that specifically related to resource allocation decisions for TCPs were identified from the literature or by Monash Health respondents with expertise in this area [27]. However, some of the objectives of system redesign are consistent with principles of disinvestment such as better use of existing resources, maximising value and eliminating waste, increasing efficiency and reducing duplication of services [56-58]. The SHARE Steering Committee decided that system redesign methods would not be used to identify opportunities for disinvestment, but may be useful in implementing decisions to disinvest.

The potential for system redesign in implementing disinvestment has been confirmed in more recent literature [5, 10, 12] and also suggested as a method to quantify disinvestment [5].

Aim 2. Disinvestment projects

Investigation of pilot disinvestment projects was proposed to understand the processes involved, assess the resources required, provide practical guidance for future projects and, if successful, be used as positive examples to promote subsequent disinvestment activities. Findings of these SHARE activities are in Paper 6 [27] and summarised below. Detailed discussion of methods and tools for identification; prioritisation and decision-making; project development, implementation and evaluation of disinvestment projects is available in Paper 10 [45].

Aim 2.1 Identification of disinvestment opportunities

Given that it would take some time to identify disinvestment opportunities from the six potential mechanisms to be investigated in Aim 1, a supplementary method was required to find suitable TCPs for immediate implementation in pilot projects in Aim 2. An 'Expression of Interest' (EOI) process where health service staff nominated their own projects was introduced to achieve this [27]. In addition to these seven methods, a range of other potential systematic methods to identify disinvestment opportunities were proposed informally during the SHARE Program but not investigated (Table 13) [27].

Although an evidence-based catalogue of disinvestment opportunities had been developed for this purpose, an *ad hoc* process whereby SHARE Steering Committee members submitted disinvestment proposals at meetings dominated the decision-making process and the catalogue was not used [27]. An algorithm for identifying disinvestment projects from the catalogue was developed (Figure 7), however the planned development of transparent criteria to be used in its application was not undertaken [27]. Two EOIs and 17 *ad hoc* proposals were investigated as potential pilot disinvestment projects [27]. The nature of the proposed change and reason for nomination are summarised in Table 14.

Three published frameworks for disinvestment also propose using applications from stakeholders in the identification process [59-61]; however the effectiveness of this approach has not been established [45, 48]. Identifying disinvestment opportunities through local proposals has been referred to as "*soft intelligence"* [48] and found by others to be unsustainable [48, 62].

Aim 2.2 Prioritisation and decision-making

The literature review found guidelines and systematic reviews for prioritisation of new and existing TCPs [63-68] and consultation identified state health department requirements. Since there were no decision-making settings where disinvestment was explicitly considered at Monash Health, the SHARE team adapted the available guidance into a tool that could apply to both investment and disinvestment, with a plan to pilot it in the annual capital expenditure funding round. The tool was not tested; the capital expenditure process was cancelled in the following two years as Monash Health had no spare capital [27].

Prioritisation tools primarily focus on characteristics intrinsic to the TCP. However the SHARE experience identified that additional criteria may influence whether a TCP is selected to be the focus of a practice change initiative; for example likelihood of success or sustainability, availability of external funds, or value of the evaluation to other processes (Tables 15 and 16). The EOI stipulated that the project must be based on high-quality evidence, be endorsed by Program and Department Heads, have appropriate resources allocated to undertake the project, have a documented clinical pathway and clear measurable outcomes, and each TCP proposed through the *ad hoc* process had one or more promising attributes [27]. However no explicit criteria were established for the decision-making committee to prioritise or make final decisions regarding pilot projects.

Decisions were pragmatic, based on likelihood of 'quick wins' and unspecified factors related to the proposed TCP. Prioritisation did occur, but the reasoning was not transparent. Of the 19 proposed TCPs, four were not investigated as the Steering Committee directed the SHARE team to disregard them in favour of subsequent proposals which were thought to have greater potential; two had incomplete investigations for the same reason; nine were rejected for a range of issues; and four were accepted as pilot projects (Table 14). Two of the four successful applications were from the EOI process and the other two had external funding from the Victorian Policy Advisory Committee on Technology (VPACT). The funding was to implement a new technology; however each had an element of disinvestment as both new TCPs were replacing a clearly identified TCP in current use.

Subsequently, lists of criteria for consideration in prioritisation and decision-making have been published for disinvestment [69, 70], resource allocation [71, 72] and general decision-making [73], and software applications are now available to facilitate prioritisation processes [53, 74].

Table 13. Additional systematic methods to identify disinvestment opportunities in a local health service

Reproduced with permission from SHARE Paper 6 Harris et al [27]

- Consider disinvestment explicitly in long term planning exercises
- Discuss principles of disinvestment and examples of successful projects at department/unit meetings, educational events, etc
- Assign member of decision-making committees to look for disinvestment opportunities in their decisions
- Add a disinvestment question to the 'Leadership Walkround' protocol
- Identify clinical champions interested in disinvestment in each program/department/unit who would look out for opportunities
- Support staff who have undertaken a disinvestment project to look for more opportunities
- Have disinvestment as a high priority in medication safety reviews
- Encourage or require projects that are introducing something new to have a component of disinvestment
- Review projects that are being conducted for other reasons and identify and focus on any disinvestment elements
- Introduce thinking about disinvestment into quality improvement training programs

Figure 7. Algorithm for identifying disinvestment projects from an evidence-based catalogue of potential TCPs

Reproduced with permission from SHARE Paper 6 [27]

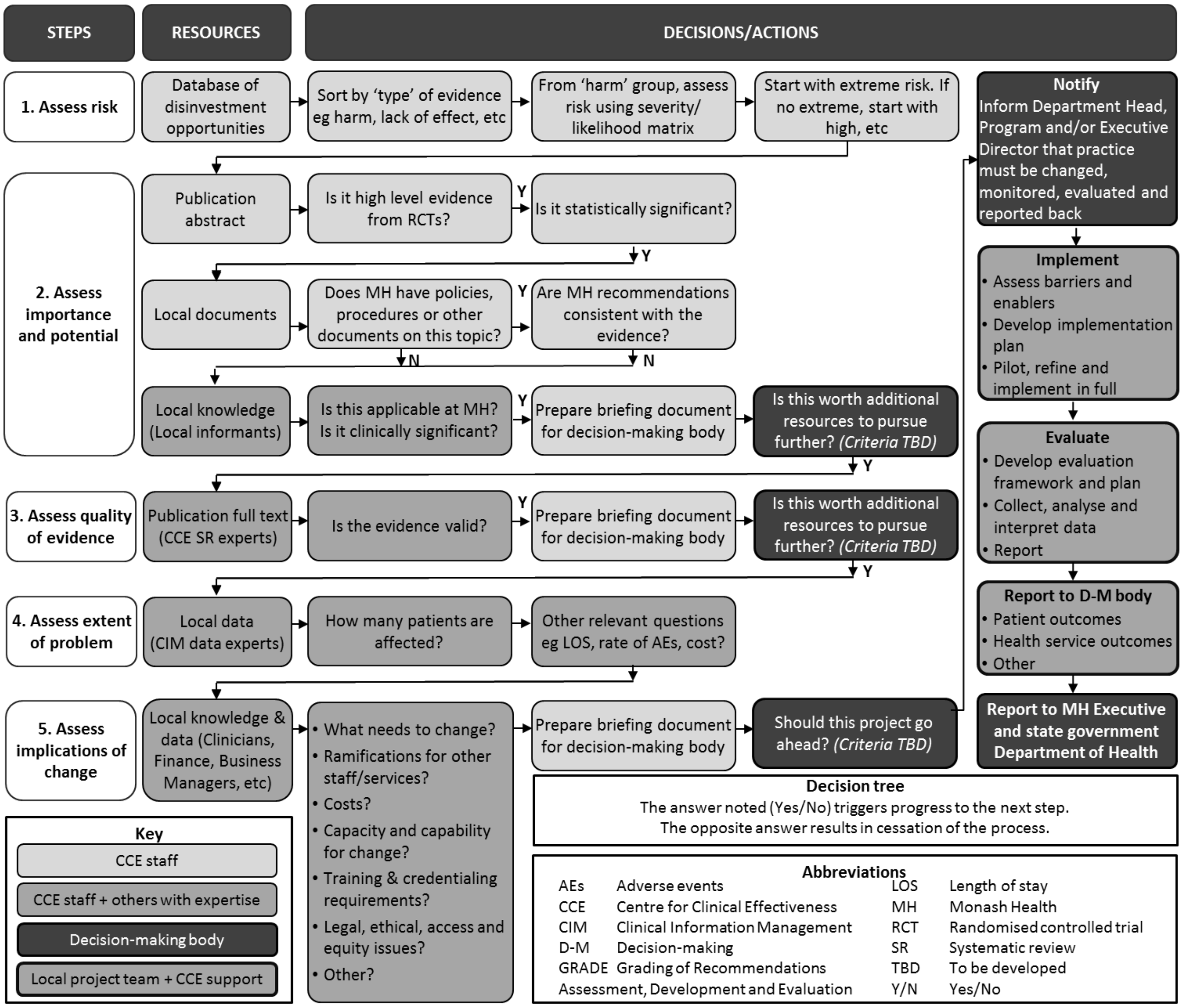

Table 14. Disinvestment projects proposed in the SHARE Program

Reproduced with permission from SHARE Paper 6 [27]

Potential projects and reason for nomination	Source	Result of investigation
1. Reduce use of therapeutic intervention due to concerns about safety and effectiveness	Committee member	**Rejected**: Lack of clarity regarding explicit problem, patient groups, etc
2. Reduce use of therapeutic intervention as thought to have no benefit over less expensive alternative	Committee member	**Rejected**: Preference to wait until large RCT underway at the time provided conclusive evidence
3. Reduce ordering of 'routine' diagnostic tests in specific setting as thought to be unnecessary and result in increased costs to hospital and/or patient, and increase risk of adverse events	Committee member	**Rejected**: Specific setting already planned to be investigated by others in organisational review but timing was unspecified
4. Reduce ordering of 'routine' diagnostic tests in specific setting as thought to be unnecessary and result in increased costs to hospital and/or patient, and increase risk of adverse events	Committee member	**Not investigated**: Further clarification of problem postponed in favour of subsequent proposals
5. Reduce ordering of diagnostic tests in specific setting due to lack of evidence of benefit and concern about validity, reliability and performance of equipment	Committee member	**Not investigated**: Further clarification of problem postponed in favour of subsequent proposals
6. Reduce ordering of diagnostic tests in specific setting as thought to be of little diagnostic value	Committee member	**Not investigated**: Further clarification of problem postponed in favour of subsequent proposals
7. Replace therapeutic intervention in specific patient group with one considered to be safer, more effective and more cost-effective and funded by state health department	VPACT project	**Accepted then became Unavailable:** Clinicians not convinced by evidence and elected to undertake RCT
8. Replace therapeutic intervention in specific patient group with one considered to be safer, more effective and more cost-effective and funded by state health department	VPACT project	**Accepted**: Project undertaken with SHARE support but evaluation incomplete due to loss of funding
9. Reduce use of therapeutic intervention in specific patient group due to concerns about patient safety, not recommended in clinical guidelines used elsewhere	Committee member	**Decision postponed**: While proposer confirmed evidence **Rejected**: When discovered that project had commenced
10. Restrict use of therapeutic intervention in specific patient group as local practice thought to be inconsistent with recently published national guidelines	Expression of interest	**Accepted then Withdrawn**: Clinicians not convinced by evidence, local practice found not to be inconsistent
11. Reduce ordering of diagnostic tests considered to be inappropriate in certain unspecified situations	Expression of interest	**Accepted then Rejected**: Inopportune timing due to external accreditation process and introduction of new computer database and electronic ordering system
12. Cease use of therapeutic intervention in specific patient group due to published debate questioning effectiveness	Committee member	**Rejected**: Evidence not relevant to patient population
13. Replace diagnostic test in specific patient group for one thought to be more appropriate	Committee member	**Investigation not completed**: Directed by Steering Committee to pursue Therapeutic Equivalence projects
14. Reduce admission of specific patient group as thought to be unnecessary in many cases	Committee member	**Investigation not completed**: Directed by steering committee to pursue Therapeutic Equivalence projects
15. Replace drug with lower cost but equally effective alternative in appropriate cases as project being undertaken anyway and it would be good way to learn about the change process	Therapeutic Equivalence project	**Rejected**: Project was already underway
16. Replace drug with lower cost but equally effective alternative in appropriate cases as project being undertaken anyway and it would be good way to learn about the change process	Therapeutic Equivalence project	**Rejected**: Project was already underway
17. Replace equipment with alternative thought to be cost-saving due to reduction in adverse events and would improve patient outcomes in specific patient group	Project champion	**Not investigated**: Project identified too late to be completed within SHARE timelines
18. Reduce ordering of 'routine' diagnostic tests in specific patient group as thought to have no evidence of benefit	Committee member	**Rejected**: Department could not provide backfill to replace project champion who would undertake project
19. Reduce use of therapeutic intervention as thought to have no evidence of benefit	Committee member	**Rejected**: Evidence for change unclear

Table 15. Examples of criteria for selection of disinvestment projects considered in the SHARE Program

Reproduced with permission from SHARE Paper 6 [27]

**Criteria in the SHARE Expression of Interest application**
▪ The project must aim to remove, restrict or replace a technology or clinical practice<br>▪ There must be high-quality evidence for the proposed change (as indicated by existing systematic review or body of evidence from peer reviewed articles)<br>▪ Department and Program heads endorse the proposed change<br>▪ Department or Program agrees to provide EFT/project leader to implement the proposed change<br>▪ The current clinical pathway is documented or a commitment is given to document this pathway before the project begins<br>▪ There are clear, measurable outcomes and ability to collect baseline and comparison data
**Criteria that may increase the likelihood of project success or sustainability**
▪ Project leaders who have the power to make change happen in their area of responsibility such as Unit Managers or Department Heads<br>▪ Project champions who are respected and trusted by the potential adopters<br>▪ Interested, engaged clinicians working in the topic area<br>▪ Available funding<br>▪ Projects that propose reallocation of resource savings
**Criteria that may be useful for selection of pilot or demonstration projects in disinvestment**
▪ Projects that are already planned for another reason that also contain an element of disinvestment<br>▪ Projects to introduce a new TCP where disinvestment of an existing practice can be made a focus of the project<br>▪ Opportunity for a 'quick win'
**Criteria that may increase the usefulness of a pilot or demonstration projects in disinvestment**
▪ Projects that are required to collect detailed data, for example reporting requirements of external funders<br>▪ Projects with robust data at baseline

Table 16. Factors for success, sustainability and suitability for disinvestment in the SHARE pilot project

Reproduced with permission from SHARE Paper 6 [27]

**SUCCESS**<br>A proposal is more likely to be successful if it meets the following criteria	**SUSTAINABILITY**<br>A proposal is more likely to be sustainable if it has appropriate and adequate provision in each category	**SUITABILITY FOR DISINVESTMENT**<br>Factors in the pilot project considered likely to be favourable for a disinvestment project at Monash Health
**Based on sound evidence or expert consensus**<br>✓ Systematic review of multiple RCTs; surgeons, nurses and allied health staff in agreement with findings<br>**Presented by credible organisation**<br>✓ Review undertaken by the Australian Safety and Efficiency Register of New Interventional Procedures – Surgical (Royal Australasian College of Surgeons)<br>**Able to be tested and adapted**<br>× *There was limited opportunity to test and adapt as the VPACT funding required complete roll out*<br>**Relative advantage is evident**<br>✓ Clear evidence of multiple improved patient and health service outcomes; increased safety and effectiveness, reduced costs<br>**Low complexity**<br>✓ The new technology is easy to use<br>**Compatible with status quo**<br>✓ Referrers use the same referral process but divide patients into those eligible for the new procedure and those who should still undergo the old procedure<br>× *The new service was provided at a different campus and patients and staff had to adapt*<br>× *There is some impact on other departments that also have to adapt*<br>**Attractive and accessible format**<br>✓ The new procedure is attractive to patients as it replaces surgery with an outpatient/bedside procedure	**Structure**<br>✓ The new procedure is carried out within existing nursing and allied health structures with appropriate governance and supports<br>**Skills**<br>✓ Nursing and allied health staff were upskilled in the new procedure; changes in scope of practice were documented and approved<br>✓ Clinical project team leaders attended training and welcomed support and direction in project management, implementation and evaluation<br>**Resources**<br>✓ Funding was provided for staffing, equipment and consumables<br>× *Final funding was less than the amount approved in the application process leaving the project short of one machine and associated consumables*<br>✓ Assistance from the Capacity Building and Project Support Services was provided<br>**Commitment**<br>✓ The project had organisational commitment from the Technology/Clinical Practice Committee, and program and departmental commitment from clinical leaders and managers<br>**Leadership**<br>✓ The clinical project team demonstrated effective leadership	✓ The current practice to be replaced and the new practice to be implemented were clear and patient eligibility was determined<br>✓ The proposal for change was clear with clear objectives<br>✓ Department and Program heads endorsed the change<br>✓ External funding was available<br>✓ The clinical pathway and referral process were documented<br>✓ Detailed data collection and reporting was a requirement of the external funding<br>✓ Baseline data had been collected and supporting data on patient group, burden of disease and impact of the new technology was available<br>✓ There was strong local ownership and clinical champions<br>✓ 'Win-win' scenario for adopters where nursing and allied health staff were keen to take on new procedural skills and surgeons were happy to relinquish these cases to make operating theatre time available for other patients<br>✓ Surgeons were allowed to keep the theatre time released by the changes and reduce their own waiting lists (rather than reallocation to other surgical specialties or closing theatres to realise savings)<br>✓ Potential 'quick win' scenario for a disinvestment demonstration project as the proposal was already fully developed, funding had been approved, and deadlines were in place.<br><br>KEY: ✓ Positive factors × Negative factors

Table 17. Factors influencing the SHARE process of selecting disinvestment projects

Based on the framework for evaluation and explication Figure 12.3 below.

Reproduced with permission from SHARE Paper 6 [27]

POSITIVE	NEGATIVE
**External environment**<br>▪ The SHARE program was adequately funded (until the final phase of the program)<br>▪ Two proposals that received state health department funding and endorsement were considered favourably.<br>▪ Two proposals were triggered by new national guidelines, one by an editorial in the Medical Journal of Australia, and others by journal articles, email bulletins, attendance at conferences and proposers awareness of practice elsewhere.	▪ The state health department withdrew funding for the final phase of the SHARE program resulting in reduction of the proposed evaluation activities.<br>▪ One project was rejected due to difficulties implementing change during the national accreditation process for this department's services.
**Organisational environment (Monash Health)**<br>▪ Monash Health encourages and supports innovation<br>▪ High level expertise was available from CCE and Clinical Information Management	▪ Waiting for responses to email correspondence and requests for appointments to meet with key personnel; time lags due to annual and long service leave and decisions by committees that only meet monthly delayed the processes of identification, prioritisation, decision-making and project development. Delays in deciding that unsuitable projects would not go ahead prevented other potentially suitable projects from being investigated.<br>▪ The proposer of one project was unaware of an existing organisational review into the problem.<br>▪ Delays related to introduction of a new computer database and electronic ordering system contributed to one project being rejected.
**Identification process**<br>▪ The 'bottom up' Expression of Interest process was the only systematic approach used, resulting in two projects being received and accepted (but both later rejected).	▪ The 'top down' evidence-based catalogue of disinvestment opportunities was not utilised in identifying potential projects.<br>▪ The 'ad hoc' process of nominations and decision-making dominated<br>▪ Most proposals were made by 'outsiders' who not involved in the nominated clinical pathway. Only two proposals were made by the potential adopters, although one subsequently withdrew their application.
**Prioritisation and decision-making process**<br>▪ All discussions were held within meetings and documented in the minutes; there were no attempts to be covert or follow hidden agendas.<br>▪ Conflict of interest was addressed as a routine agenda item.<br>▪ All clinical programs, health professional disciplines, consumers and technical experts in evidence, data, legal, ethics, finance, purchasing, biomedical engineering and information technology were represented in decision-making.	▪ There were no explicit processes for risk assessment, deliberation or appeal. It was not always clear how decisions had been made.<br>▪ The SHARE Steering Committee did not have authority to direct change. Proposals were put to department heads who declined to follow them up (based on reasoned arguments that they should not to go ahead).
**Rationale and motivation**<br>▪ Safety and effectiveness were the primary reasons for nominating TCPs for disinvestment, cost-savings were a secondary benefit	
**Proposal for change**<br>▪ Six proposals were submitted based on guidelines, systematic reviews or health technology assessments; the four accepted projects were in this group.<br>▪ Four proposals had supporting data, two regarding unnecessary diagnostic imaging tests and the two VPACT projects.<br>▪ The two VPACT projects presented defined objectives.<br>▪ One project had a clear reinvestment plan which allowed operating theatre time previously used by patients now undergoing the new non-surgical procedure to be used by other patients	▪ In 13 proposals, the nominator did not provide supporting evidence.<br>▪ Many of the proposals did not clearly define the TCP, patient population group, circumstances of restriction, etc. This is difficult to quantify as clarification may have been forthcoming but the proposals were not investigated further

on the waiting lists, this was the implemented pilot project.	
**Potential adopters**<br>▪ Three nominations were made by the potential adopters; one was the pilot project accepted and implemented, one was accepted as a pilot project but was subsequently withdrawn by the applicants and the other was nominated too late to be included in the SHARE timeframe	▪ Decisions regarding eight proposals were declined by heads of the departments responsible for the proposed TCP. Reasons included lack of clarity of the problem, lack of supporting evidence, or the evidence was not relevant to local patient groups.<br>▪ In two of the accepted projects, the key adopters reversed their decisions about the supporting evidence and withdrew.
**Potential patients**	▪ Two proposals were rejected when it became clear that the evidence did not apply to the Monash Health population.
**Implementation and evaluation plans and resources**<br>▪ The CCE/SHARE support staff had appropriate expertise and knowledge of methods and tools for implementation and evaluation.<br>▪ The CCE team provided access to research literature and liaised on behalf of the clinical project teams with the Clinical Information Management (CIM) unit who were happy to provide access to data and assistance with analysis.<br>▪ All implementation activities within the control of the SHARE project team were completed<br>▪ Detailed evaluation plans were developed in consultation with an external health program evaluator and health economist<br>▪ One proposal had assistance of a research fellow to undertake the project work (but this did not go ahead for other reasons).<br>▪ The clinical project leads of two accepted projects attended workshops in evidence-based change, implementation and evaluation	▪ Lack of evaluation funding precluded understanding of the barriers that prevented implementation of the planned systematic evidence-based processes<br>▪ Lack of evaluation funding limited evaluation activities in the last year of the program<br>▪ One project was rejected by the department head because they could not provide backfill for the clinical duties of the project leader.

Table 18. Factors influencing the SHARE pilot disinvestment project

Based on the framework for evaluation and explication Figure 12.3 below.

Reproduced with permission from SHARE Paper 6 [27]

POSITIVE	NEGATIVE
**External environment**<br>▪ The project funders had significant impact on the project<br>– Political support for new technology<br>▪ The other health services in the consortium also had significant impact<br>– Collaboration with some of the other health services in writing pathway and documents and developing database and implementation strategies was helpful<br>▪ Manufacturer's information was useful<br>▪ Manufacturer's technical representative was helpful	▪ The project funders had significant impact on the project<br>– Monash Health informed that they had to lead a consortium of health services in implementing the new technology, adding complexity to the original application<br>– Lack of consultation in choice of partner health services<br>– Requirements for data collection and reporting changed during the project<br>▪ The other health services in the consortium also had significant impact<br>– Slow and difficult to coordinate when working with other health services<br>– Lack of accountability in some of the other health services<br>– Lack of 'buy-in' from other health services through the entire process
**Organisational environment (Monash Health)**<br>▪ Monash Health's reputation as a leader will facilitate new technology support<br>▪ Monash Health encourages innovation<br>▪ Support from Centre for Clinical Effectiveness (CCE)<br>▪ Support from Clinical Program Directors<br>▪ Support from Finance Department and having someone who can translate the finance jargon<br>▪ Clinical Resource Nurse monthly meetings<br>▪ Nursing/Allied Health collaboration<br>▪ Although staff leave and secondments are difficult there can also be an advantage of working with replacement staff who become familiar with the project	▪ Organisational processes appear to be changing regularly<br>▪ Lack of clarity around organisational structures and processes eg who to go to for what, when etc<br>▪ Lack of communication eg machine delivered to a corridor on a Friday afternoon and left unsecured over the weekend. A component was lost and a new component had to be purchased.<br>▪ Relevant patient group and clinical expertise in this area located at site A and new machine is at site B. Patients usually scheduled for surgery at A will have to transfer to B.<br>▪ Sites have different cultures and processes and patients and staff will have to adapt<br>▪ Impact on other departments eg Sterilisation department has to learn new procedure<br>▪ Staff secondments and/or leave
**Identification process (VPACT application process for introduction of new TCP)**<br>▪ Proposed by potential adopters (nursing/allied health and surgeons)<br>▪ Support from CCE to provide supporting evidence<br>▪ Support from Clinical Information Management to provide supporting data	▪ Application form is really long and a lot of work<br>▪ Lack of awareness of the workload prior to commencing work on application
**Prioritisation and decision-making process (SHARE process to determine disinvestment project)**<br>▪ VPACT funding and endorsement<br>▪ Clinical project team keen to access CCE expertise and support for project delivery	
**Rationale and motivation**<br>▪ To reduce harm, improve patient outcomes, improve service efficiency, save money	▪ Emphasis on financial/economic outcomes
**Proposal for change**<br>▪ There is good evidence to support the new technology<br>▪ Data on patient group, burden of disease, impact of new technology provided in detail<br>▪ New technology does not cause long lasting/irreversible damage<br>▪ Easy to use<br>▪ Proposal for change is clear<br>▪ Relative advantage is clear: improved outcomes for both patients and health service<br>▪ Endorsed by clinical leaders, good local engagement, clinical champions<br>▪ Surgeons allowed to keep the theatre time and reduce their own waiting lists (rather than reallocating to other surgical specialties or closing theatres to realise savings)	▪ Longer time to set up than other treatment options<br>▪ Lots of protective clothing which can be uncomfortable<br>▪ Mentally and physically tiring<br>▪ The whole process of change including administration, training, support, etc is a lot of work

**Potential adopters (Nursing and Allied Health staff to undertake new procedure, surgeons to reduce old procedure, junior medical staff to refer patients appropriately**	
▪ Most surgeons happy to relinquish old procedure to allow them to undertake other procedures<br>▪ Surgeons involved in VPACT application have become an authority on the new technology<br>▪ Senior clinical staff read up on new technology as they don't want to lose face<br>▪ Registrars (referrers) are supportive of/have an interest in new technologies<br>▪ General interest among staff<br>▪ Nursing/Allied Health team look professional, able to build credibility and trust with patients	▪ One group of surgeons less likely to refer patients for new procedure, do not appreciate role of podiatrist in patient care, lack of understanding of treatment options<br>▪ Some surgeons/medical staff have issues with territorialism and ego
**Potential patients**<br>▪ Patients with chronic conditions are more open to trying new treatments	▪ This group of patients are less likely to be comfortable travelling to different hospitals<br>▪ Lack of English language can be a problem
**Implementation plan**<br>▪ Small training workshops with medical teams<br>▪ Support from CCE<br>▪ Support from Clinical Program Directors<br>▪ Maintenance of a booking system<br>▪ Quarterly meetings with all participating health services	▪ Should have performed barriers and enablers analysis earlier in process<br>▪ Involvement of other hospitals with staff who are not dedicated/committed (eg disputes among doctors from another site)<br>▪ Having to repeat training every 3-6 months due to staff rotations<br>▪ Attrition of podiatrists and Clinical Nurse Consultants as they are often young women who leave or work part-time to have or care for children<br>▪ Keeping the team motivated is hard<br>▪ VPACT did not meet costs stipulated in application; fewer machines, limited consumables, etc<br>▪ Lack of dedicated treatment room increases time for preparation and cleaning. Clinical time is small in comparison to set up/clean up time. Inadequate ventilation (aerosols are created with treatments)
**Evaluation plan**<br>▪ Support from CCE in development of evaluation plan<br>▪ Having a person in charge of data entry	▪ 'Shifting the goal posts' by VPACT regarding data collection and reporting
**Implementation and evaluation resources**<br>▪ Other clinical staff voluntarily take up extra workload (both barrier and enabler)<br>▪ Support from CCE in design of a database, assistance with data entry and reporting<br>▪ Support from SHARE health economist in development of cost-comparison plan<br>▪ Monash Health 'Scope of practice' processes and documents were helpful	▪ Inadequate funding for clinical staff to implement and evaluate change process<br>▪ Other clinical staff voluntarily take up extra workload (both barrier and enabler)<br>▪ Time needed to write up new scope of practice documents

Aim 2.3 Development, implementation and evaluation of disinvestment projects

No published guidance regarding development, implementation or evaluation of disinvestment projects in the local health service context was identified [2, 4]; however Monash Health staff provided details of strengths, weaknesses, barriers and enablers in these processes (Table 4) [14] and needs for assistance to undertake projects [15]. Implementation and evaluation methods were planned for the SHARE disinvestment pilot projects, however only one reached the implementation stage and evaluation was limited due to the reduction of funding in the final year [27].

The overview of the literature includes a discussion of available methods and tools for disinvestment projects [45].

Influencing factors

Factors influencing the SHARE process for identification, prioritisation and decision-making, implementation and evaluation of potential projects and those influencing the pilot project selected are outlined in Tables 17 and 18.

Aim 3. Support services

Although Monash Health staff identified evidence from research and local data as key elements of decision-making, local research confirmed the findings of other studies that evidence from research and local data is not used systematically or proactively to drive decisions; that health service personnel usually lack the time, knowledge, skills and resources to access and identify the information they require and appraise it for quality and relevance; that clinicians charged with undertaking projects commonly do not know how to implement and evaluate change or manage projects effectively; and that projects are generally under-resourced [3, 14, 16, 26, 75-80]. Respondents were aware of their limitations and those of their colleagues in undertaking projects and they welcomed advice and support [15]. Four support services were proposed to address these barriers in Aim 3 (Figure 5). Details of these investigations are reported in Papers 7 and 8 [15, 43] and an overview and summaries of factors that influenced development, processes and outcomes of the support services are found in Figure 8 and Tables 19 and 20.

The effectiveness of evidence products and capacity building strategies to address the need for education, training, support and assistance from experts to enable EBP is well documented [2, 14, 16, 26, 75-78] and in-house 'resource centres' have been proposed as a solution [10, 59, 81-83] but, other than capacity building for research [84], we were unable to find any examples that had been evaluated.

Figure 8. Overview of investigation of the SHARE Support Services

Reproduced with permission from SHARE Paper 7 [15]

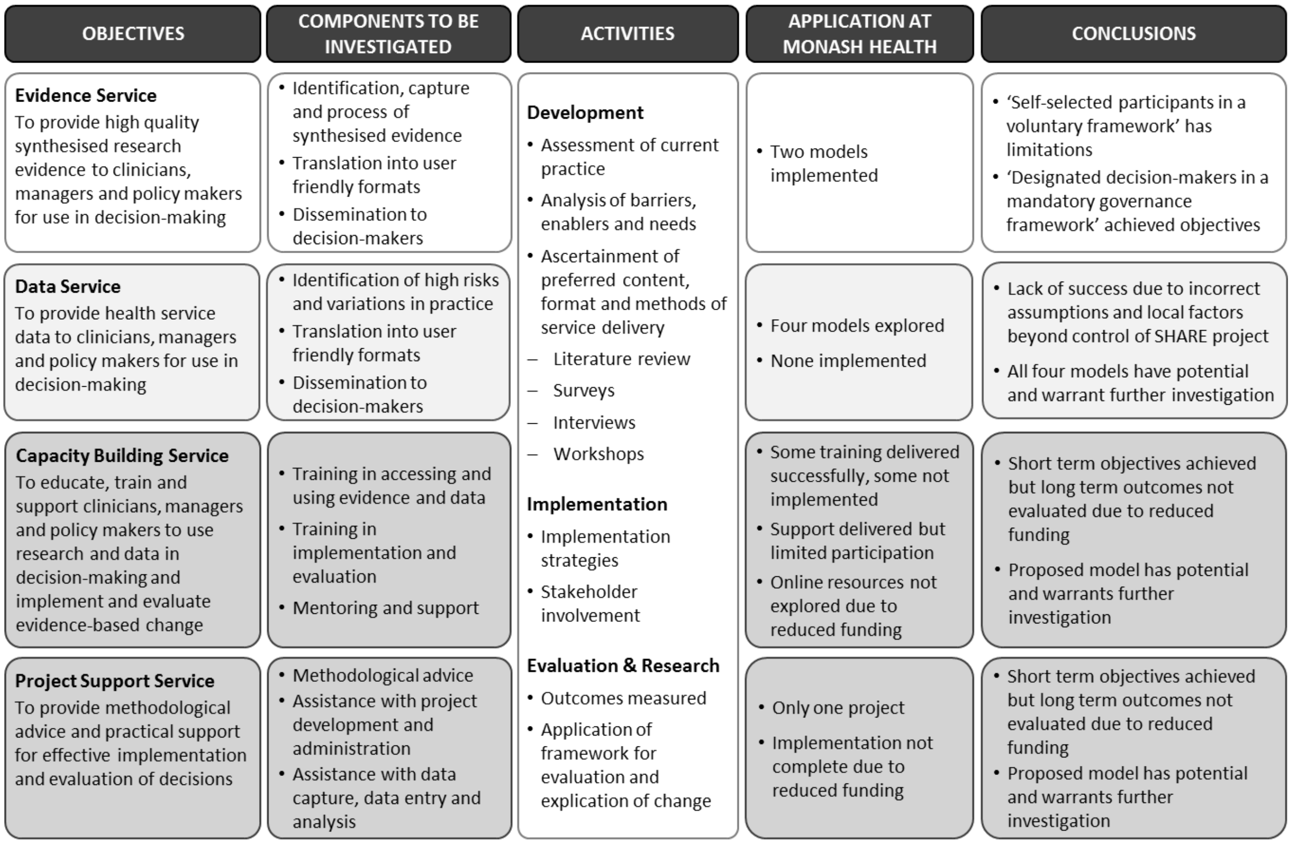

Table 19. Factors that influenced decisions in development of the SHARE support services

Reproduced with permission from SHARE Paper 7 [15]

	EVIDENCE SERVICE			DATA SERVICE			CAPACITY BUILDING SERVICE			PROJECT SUPPORT SERVICE		
	Identify, capture and process synthesised evidence	Translate into user friendly formats	Disseminate to decision-makers	Identify high risks and variations in practice	Translate into user friendly formats	Disseminate to decision-makers	Provide training in accessing and using evidence and data	Provide training in implementation and evaluation	Mentor and support	Provide advice regarding methodologies and methods	Assist with project development & administration	Assist with data capture, data entry and analysis
**BARRIERS**												
Lack of time and opportunity [16, 18, 76, 77, 80, 85-93]			✓			✓					✓	✓
Lack of skills [10, 16, 18, 49, 54, 77, 78, 80, 86-88, 90, 92-95]	✓	✓	✓	✓	✓	✓	✓	✓	✓	✓	✓	✓
Lack of confidence [16, 96]	✓	✓	✓	✓	✓	✓	✓	✓	✓	✓	✓	✓
Lack of interest or competing priorities [75, 87, 93, 96, 97]			✓			✓	✓		✓			
Lack of awareness of research and data [10, 16, 75, 78, 80, 90]	✓		✓	✓		✓	✓					
Lack of use of available research and data [10, 50, 75, 78, 95]		✓	✓		✓	✓	✓	✓	✓	✓	✓	
Lack of relevant research and data [77, 78, 85, 87-89, 91, 92, 94, 98] particularly for disinvestment [30, 49, 62, 92, 93]	✓			✓			✓					
Poor quality of health data [78, 85, 92, 95, 98-100]				✓	✓	✓						
Unfamiliar or difficult to use formats of research and data [16, 78, 90, 92, 93, 95, 98]		✓			✓		✓					
Lack of policies and interventions for data-informed decision-making [78, 85, 101]				✓	✓	✓						
Difficulty accessing or using online resources [16, 18, 75, 77, 78, 86-88, 90-92, 94, 98]	✓			✓			✓		✓			
Lack of infrastructure and technical support [10, 16, 76, 85, 91, 93, 95, 99, 102]	✓	✓	✓	✓	✓	✓	✓	✓	✓	✓	✓	✓
Inadequate resources [76, 77, 85, 87, 91, 101, 102]	✓		✓	✓		✓					✓	✓
Negative attitudes or resistance to change [16, 76, 80, 93]		✓			✓		✓	✓				
Professional groups with different perspectives of evidence, knowledge base and skill set [62]								✓	✓			
Lack of triggers to initiate disinvestment discussions [5, 50, 82, 103]			✓			✓						
Lack of standardised processes for project delivery, responsibilities and accountability [49, 53, 104]								✓	✓	✓	✓	✓
Unrealistic project timelines [104]								✓	✓	✓	✓	✓
**ENABLERS**												
Training in use of evidence and data [16, 86, 90, 95, 99, 101]							✓	✓	✓	✓	✓	✓
Dissemination of research and data [77, 90, 101, 105]			✓			✓						

	EVIDENCE SERVICE			DATA SERVICE			CAPACITY BUILDING SERVICE			PROJECT SUPPORT SERVICE		
	Identify, capture and process synthesised evidence	Translate into user friendly formats	Disseminate to decision-makers	Identify high risks and variations in practice	Translate into user friendly formats	Disseminate to decision-makers	Provide training in accessing and using evidence and data	Provide training in implementation and evaluation	Mentor and support	Provide advice regarding methodologies and methods	Assist with project development & administration	Assist with data capture, data entry and analysis
Clarity, relevance, credibility and reliability of research findings [77, 90, 92, 106, 107]	✓	✓					✓					
Quality and timely data from health information systems [78, 92, 95]				✓	✓		✓					
Organisational willingness to invest in a knowledge translation culture [76, 101, 108]	✓	✓	✓	✓	✓	✓	✓	✓	✓	✓	✓	✓
Infrastructure or policy for accountability in knowledge use [76, 101]			✓			✓						
Links to researchers or knowledge brokers [76, 77, 92, 108, 109]			✓			✓	✓	✓	✓	✓	✓	✓
Initiatives to integrate data into routine decision-making processes [105]				✓	✓	✓						
**ADDITIONAL NEEDS**												
Capacity-building and provision of expertise and practical assistance [8, 10, 59, 78, 83, 90, 100]	✓	✓	✓	✓	✓	✓	✓	✓	✓	✓	✓	✓
New processes to use research and data 'proactively' to drive decisions [59, 78, 99, 100]	✓	✓	✓	✓	✓	✓						
Analysis, synthesis, interpretation and review of data in decision-making [78, 95, 99]				✓	✓	✓	✓	✓	✓	✓		✓
Incentives to change [50, 53, 101]										✓	✓	✓
Support to be tailored to units and professional needs [78, 107, 108]		✓			✓		✓	✓	✓	✓	✓	✓
Provision of a range of expertise in evaluation methods [84, 99]										✓	✓	✓
Support from others who had done the same or similar work to address feelings of isolation							✓	✓	✓	✓	✓	✓
**EVIDENCE-BASED INTERVENTIONS**												
Dissemination of summaries of systematic review evidence [75, 110, 111]		✓	✓									
Tailored targeted messages [75, 112-114]		✓	✓		✓	✓						
Training in critical appraisal [111, 113, 115]							✓		✓			
Interactive workshops [75, 115]							✓	✓	✓			
Multifaceted educational intervention [75, 115]							✓	✓	✓	✓	✓	✓

Table 20. Factors that influenced processes and outcomes of the SHARE support services

Reproduced with permission from SHARE Paper 7 [15]

CHARACTERISTICS OF THE DETERMINANTS OF EFFECTIVENESS*			ES[#]	DS	CBS	PSS
**External environment**	Political	Disinvestment was a priority topic for Department of Treasury which encouraged Department of Human Services to investigate it further	✓	✓	✓	✓
	Financial	Department of Human Services funding for SHARE enabled all the activities	✓	✓	✓	✓
		Withdrawal of funding in final year of program prevented implementation of some interventions and many of the evaluation activities		×	×	×
**Organisation**	Financial	Monash Health funding for SHARE also enabled all the activities	✓	✓	✓	✓
		Monash Health funding for ES continued after Department of Human Services funding withdrawn	✓			
	Leadership	Support and endorsement was provided at senior levels (Board, Executive Management Team, Clinical Program Directors)	✓			
	Processes	Monash Health had multiple databases, housed with different custodians, with a range of methods of access; there was no coordination		×		
		Evidence Service was implemented in a governance framework requiring mandatory responses from decision-makers	✓			
	Culture	Organisational (ES) and departmental (CBS) culture was supportive of evidence-based practice	✓		✓	
**Potential adopters**	Attitudes	Most target users viewed the proposals positively	✓		✓	✓
		Target users acknowledged their limitations, were enthusiastic about training and support and were willing to take advice and direction			✓	✓
		Committees declined support in accessing and using data		×		
	Support	Pharmacy staff had support from management to attend training			✓	
	Leadership	Pharmacy staff, pharmacy-related committee members and SHARE pilot project teams demonstrated leadership by their participation			✓	✓
**Innovation**	Evidence	Developed from research and local data identifying barriers, enablers and expressed needs for content and format	✓	✓	✓	✓
		Good supporting evidence of effectiveness of chosen interventions	✓	✓	✓	✓
	Engagement and champions	Centre for Clinical Effectiveness has ownership of the project and authority to implement change	✓		✓	✓
		Centre for Clinical Effectiveness does not have ownership of the project and authority to implement change		×		
	Compatibility with status quo	Within Centre for Clinical Effectiveness skill sets and priorities	✓		✓	✓
		Not within Centre for Clinical Effectiveness skill sets and priorities		×		
		Proposal is not deliverable in original format (multiple often inaccessible datasets, lack of local capacity and capability)		×		
	Trialability	All services were implemented in pilot mode and participants were informed that their feedback would be used to refine the processes	✓		✓	✓
		Implementing with small groups resulted in lack of critical mass for ongoing support services			×	
**Implementation strategy**	Tailored to barriers and enablers	Barrier and enabler analysis focused on development of the innovation and not on development of implementation strategies	×	×	×	
		Tailored to needs of individual projects and project teams				✓
	Knowledge and skills	Centre for Clinical Effectiveness team had skills in implementation of change	✓		✓	✓
		Health economist and health program evaluator engaged as consultants to the project team	✓		✓	✓
	Resources	Adequate resources initially	✓	✓	✓	✓
		Inadequate resources after Department of Human Services funding withdrawn		×	×	×
* Not all factors from the taxonomy are listed, only those that influenced the pilot projects. Some factors only influenced some of the support services.		# ES = Evidence Service, DS = Data Service, CBS = Capacity Building Service, PPS = Project Support Service<br>✓ = Positive influence, × = Negative influence				

Aim 3.1 Evidence Dissemination Service

The Evidence Dissemination Service (EDS) was conceived as a method of identifying disinvestment opportunities by delivering recently published, high quality, synthesised evidence directly to decision-makers [43]. But it became clear during development that this could be a way to ensure that all practice at Monash Health was consistent with current evidence. Two models were implemented (Figure 9).

Figure 9. Development, implementation and evaluation of an in-house Evidence Dissemination Service

Based on UK Medical Research council framework for evaluation of complex interventions (three phases) [1] and the SEAchange model for sustainable, appropriate and effective evidence-based change (four steps) [116].

Reproduced with permission from SHARE Paper 8 [43]

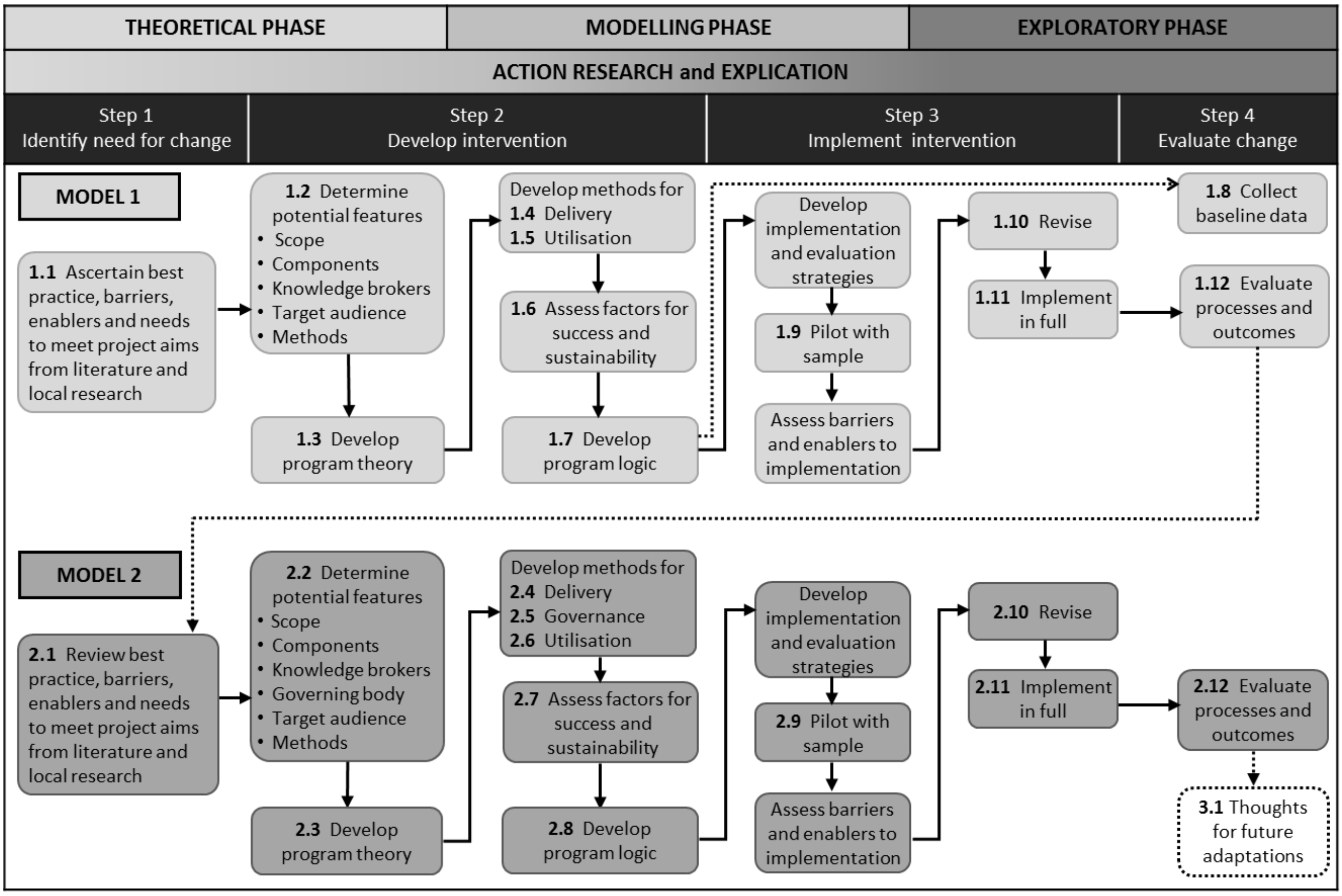

Model 1 involved identification, capture, classification and storage of eligible publications; repackaging into user-friendly formats; and dissemination to decision-makers. 'Evidence Alerts' were sent weekly by email or RSS feed and publications were stored in a searchable website. Alerts contained citations which were hyperlinked to abstracts which were hyperlinked to full text. This was undertaken by the EDS team, knowledge brokers from CCE. Decision-makers were required to subscribe to receive the Alerts, appraise the evidence for quality and local applicability, take appropriate action, and report the decisions and actions within their routine monthly reporting structure using a template developed for this purpose.

This model could not achieve its aims. The main factor was lack of governance; there was no process to ensure that the appropriate person with authority in the area affected by the evidence had considered the information, made a decision or taken any action. The second factor was lack of time to undertake the steps required; this was reported by both the EDS team and the decision-makers. In addition, many publications were already known to recipients, not relevant to their area of practice, not applicable at Monash Health, consistent with current practice, not important enough to instigate change or reported lack of evidence; hence required no action. This resulted in time wasted by both the EDS team and the decision-makers.

Model 2 addressed these issues by adding a governance element to ensure the evidence was reviewed and acted upon by the appropriate decision-makers, by limiting selection of publications to areas of high priority to reduce the workload of the EDS team, and by reallocating most of the decision-maker's activities to either the EDS team or the new governing body to reduce the workload of busy clinicians and managers (Figure 10). The Technology/Clinical Practice Committee

(TCPC) already had the authority to require responses from organisational decision-makers and impose changes in practice related to introduction of new TCPs and was deemed an appropriate body to undertake governance of TCPs in current use.

Figure 10. Comparison of stakeholder roles in two models for an in-house Evidence Dissemination Service

Reproduced with permission from SHARE Paper 8 [43]

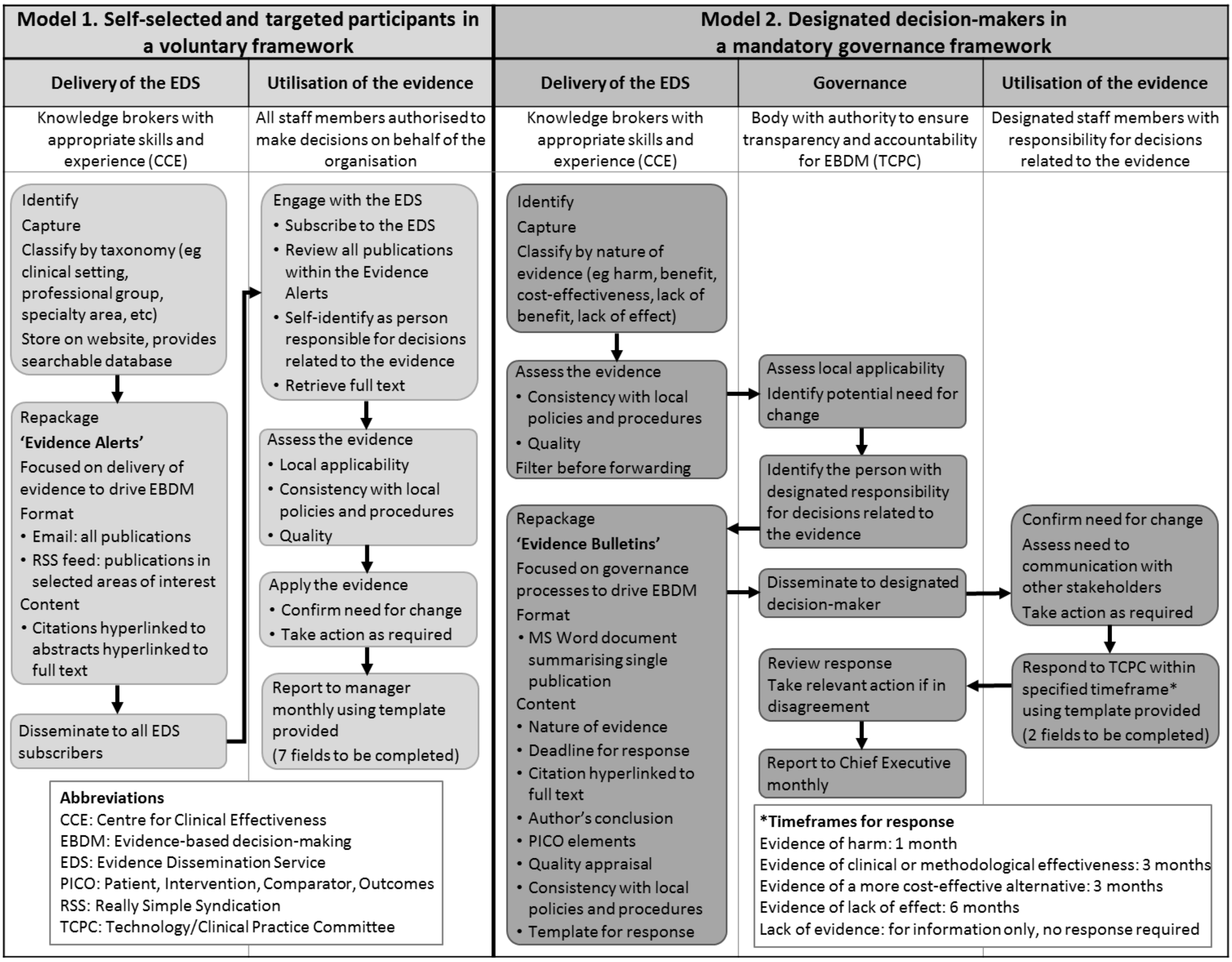

In the final version of Model 2, processing of publications was limited to those demonstrating evidence of harm, lack of effect and availability of a cost-effective alternative which were priorities of Monash Health at the time. The findings of these studies were compared with current documented practice in local policies and procedures. If there was no local documentation, or it was inconsistent with the evidence, the publication was appraised for quality and forwarded to the TCPC to assess local applicability and identify the relevant organisational decision-maker, usually a department head or committee chair. The EDS team then developed an 'Evidence Bulletin' which included information extracted from the publication, the quality appraisal findings and a reporting template (Figure 11). Bulletins were sent to the relevant Executive or Program Director who forwarded them to the authorised decision-maker within their portfolio with a request to confirm whether current practice was consistent with the evidence, and if not, what measures were being taken to address this, or an explanation of why change was not required. The Chief Executive determined that this was an organisational priority; when there was evidence of harm, responses to the TCPC were required within one month and were reported to the Chief Executive the following month. Responses to other Evidence Bulletins were required in three or six months. Publications containing evidence of effectiveness or lack of evidence were not processed and were disseminated for information only, no response was required.

While this was successful in aligning local practice with current evidence, it was a very resource-intensive process and CCE had insufficient staff capacity to maintain it while meeting other commitments. The EDS was suspended in the last few months of the SHARE Program, however it has subsequently been reinstated and is focused on the 'Choosing Wisely' literature [117].

Figure 11. Example of an Evidence Bulletin

Reproduced with permission from SHARE Paper 8 [43]

Southern Health

Technology/Clinical Practice Committee

Evidence Bulletin_164

This bulletin is part of a process to ensure that Southern Health practice is consistent with current evidence. Your response is required by the date below. You can find more information about this process on the TCPC website.

The publication below indicates evidence of **Potential HARM (due to significant adverse events/side effects but lack of evidence of effectiveness)** related to Tricyclic antidepressants for autism spectrum disorders (ASD) in children and adolescents.

Responses related to evidence of **Potential HARM** are required within **ONE month.**

Please complete and return this bulletin to marie.garrubba@monash.edu by **11 June 2012**

Bibliographic Source

Hurwitz R, Blackmore R, Hazell P, Williams K, Woolfenden S. Tricyclic antidepressants for autism spectrum disorders (ASD) in children and adolescents. Cochrane Database of Systematic Reviews 2012, Issue 3. Art. No.: CD008372. DOI:10.1002/14651858.CD008372.pub2. http://onlinelibrary.wiley.com/doi/10.1002/14651858.CD008372.pub2/pdf

Author's Conclusion

Clinicians considering the use of TCAs need to be aware of the limited and conflicting evidence of effect and the side effect profile when discussing this treatment option with people who have ASD and their carers. Further research is required before TCAs can be recommended for treatment of individuals with ASD.

Applicability to Southern Health

**Patient / Population**	Inclusion was limited to children and adolescents (birth to 18 years of age) with a diagnosis of an autism spectrum disorder (ASD), using a standardised diagnostic instrument (for example, ADOS, ADI-R, DISCO, CARS) or using established diagnostic criteria as defined by DSM-IV or ICD-10, that is Pervasive Developmental Disorder, excluding Rett Syndrome and Childhood Disintegrative Disorder.
**N**	3 studies – number of participants unclear
**Setting**	Outpatient setting
**Intervention**	Any oral tricyclic antidepressants, regardless of dosage used, duration of use or frequency of administration.<br>Tricyclic antidepressants include amitriptyline (amitriptyline hydrochloride), amoxapine, clomipramine (clomipramine hydrochloride), dothiepin (dosulepin hydrochloride or dothiepin hydrochloride), doxepin, imipramine (imipramine hydrochloride), iofepramine, nortriptyline, trimipramine, desipramine, florpiramine, dibenzepin, iprindole, protriptyline and modified tricyclic antidepressants such as tianeptine.
**Comparison**	Placebo
**Outcomes**	**Primary outcomes**<br>• Core symptoms of autism, for example, impairments in communication, reciprocal social interaction and behavioural problems, such as repetitive behaviours and rituals, obsessional behaviour and stereotypy.<br>• Non-core symptoms, including challenging behaviours, sleep disturbance and aggression.<br>• Comorbidities, including depression and anxiety.<br>• Adverse effects.<br>**Secondary outcomes**<br>• Parental, child or family quality of life.<br>• Parental or family stress.<br>We planned to examine short-term (up to three months), medium term (three to 12months) and long-term (greater than 12 months) outcomes if the data were available.<br>We used the primary and secondary outcomes to populate the 'Summary of findings' tables.<br>**Types of measures:**<br>1. Standardised diagnostic assessment instruments (Childhood Autism Rating Scale, Autism Diagnostic Interview- Revised, Autism Diagnostic Observation Schedule, Diagnostic Interview for Social and Communication Disorders).<br>2. Standardised communication assessments.<br>3. Quality of life questionnaires.<br>4. Rating scales of emotions and behaviour, including depression, anxiety, aggression, obsessive-compulsive behaviour and social reciprocity.<br>5. Global Clinical Impression Rating Scales.<br>6. Other Health Outcome Rating Scale.
**Inclusion Criteria**	Randomised controlled trials (RCTs).
**Exclusion Criteria**	

Quality of Evidence

Quality of this Systematic Review or Health Technology Assessment

CCE staff appraised the methods used in this publication and found the **risk of bias** to be **LOW.** This means that you can use the findings of the review with confidence as all of the quality criteria have been fulfilled or where criteria have not been fulfilled it is very unlikely the conclusions of the study would be affected.

Quality of the *evidence contained* in this Systematic Review or Health Technology Assessment

The review authors appraised the available evidence and found it to consist of **Level II Evidence (one or more randomised controlled trials)**. The available evidence included in the review is of **variable quality.**

Consistency with Southern Health documented practice

No Southern Health policies or procedures on this topic were identified.

Response

- Click once on the shaded box to select the appropriate response
- Click once on the shaded rectangle to provide a typed comment

Practice at Southern Health (please select one response only, tick the box and provide relevant details)

☐ Not applicable at Southern Health *eg the patient group is not treated at Southern Health* (please explain)

☐ Practice is consistent with the evidence (please add comments if relevant)

☐ Practice is not consistent with the evidence for a good reason (please explain)

☐ Practice was not consistent with the evidence, remedial action has been undertaken and completed (please explain)

☐ Practice is not consistent with the evidence and remedial action has been commenced/planned (please explain)

Communication

Should this information be disseminated more widely? If so, to whom?

Other comments

Feedback

This is a pilot of new processes being implemented by the Technology Clinical Practice Committee and the Centre for Clinical Effectiveness Evidence Dissemination Service.

We would appreciate any comments regarding what works, what doesn't work and how we can improve the process.

**Name:**			
**Position:**		**Date:**	

Thank you

This study provides the details of a systematic process for recently published, high quality, synthesised evidence to be *"captured from outside, circulated internally, adapted, reframed, implemented, and routinized in a service organization"* [117]. To our knowledge, this is the only report of development, implementation and evaluation of an in-house EDS implemented in a governance framework within a local healthcare setting.

Existing evidence services deliver bulletins on selected topics to individual subscribers [118-120]. Types of evidence products have also been defined [121]. There are many similarities between these examples and the SHARE EDS, however there are several key differences between the models explored here and those trialled by others.

The main distinctions are related to the in-house systematic approach to using evidence proactively to ensure organisational practice is consistent with current evidence.

Many studies have explored the characteristics and use of publications as evidence products [90, 107, 121-130]. In addition to content and format of the products, others have noted the need to target individual decision-makers [121, 123, 126] who are authorised to implement change [16, 51, 54, 77, 91, 131, 132] with timely [75, 133] and locally relevant information [90, 107, 121]; actively deliver the evidence directly to decision-makers [126, 133]; create an organisational culture supportive of EBDM [121, 126]; make use of existing formal infrastructure [16, 102, 133, 134] in a governance framework to provide legitimacy and engagement [135] particularly in the case of disinvestment where a governance committee is thought to "*make contentious decisions more palatable and defensible*" [10, 53, 136, 137]; and clearly identify requirements for accountability [91, 124, 127, 135] including mandated responses [138] and use of reporting tools [135].

The EDS Model 2 may be the first to integrate all of these. It builds on earlier findings by focusing on new organisation-wide systems and processes embedded in existing infrastructure, such as CCE, TCPC, authorised decision-makers, and reporting networks, in which to disseminate evidence within a governance framework.

The Evidence Bulletins had elements of each of the defined categories [121] – summaries, overviews and policy briefs – but they also had critical differences with other disseminated evidence products (Table 21).

Table 21. Unique characteristics of the SHARE EDS

- The nature of the evidence, such as evidence of harm, clinical or cost-effectiveness, lack of effect, or lack of evidence, was defined for each publication and used to determine the next steps for knowledge brokers and decision-makers.
- Each article was critically appraised for quality and an appraisal summary and its implications was provided for the reader; low quality reviews were not disseminated.
- Local implications were considered.
  - Publications were only disseminated if they were inconsistent with organisational policies and protocols or there was no relevant local guidance on this topic.
  - Applicability was assessed by senior managers prior to dissemination and PICO characteristics were extracted and summarised to enable the authorised decision-maker to confirm local applicability.
- Specific time-critical actions were required of the recipients; for example in the case of evidence of harm, decision-makers had to determine whether practice change was required, develop a plan for action, and respond with the details within one month.
- The governance elements ensured transparency through clear systems and processes and accountability through reporting requirements. The EDS was given high priority by the Chief Executive who instigated the mandatory responses and implementation was integrated into the organisational Business Plan.

Aim 3.2 Data Service

The Data Service was initiated to complement the EDS by delivering local data to decision-makers. The aims were 1) to interrogate routinely-collected data to identify potential disinvestment opportunities and communicate this information to appropriate decision-makers; 2) to respond to requests from decision-makers to assess local data related to potential disinvestment opportunities that had been identified from the research literature; and 3) to provide training, advice and support in accessing and utilising local data to the Capacity Building and Project Support Services [15]. Investigation of routinely-collected data would include:

- patterns of current practice to identify areas where disinvestment might have the greatest impact such as high volume; high cost; high rates of mortality, adverse events, readmission, reoperation; and long length of stay.
- variations in practice that might indicate overuse or inappropriate practices, for example between sites, departments and individuals at Monash Health; between Monash Health and similar health services; or over time.

Four models of a Data Service were explored, but none were implemented due to local factors such as limited staff capacity and problems with local data access and coordination [15]. As a result, proactive use of health service data was not employed to identify disinvestment targets for pilot projects.

Aim 3.3 Capacity Building Service

The aim of this service was to train and support staff to use research evidence and data in decision-making and then implement and evaluate these decisions in successful projects [15]. The proposed activities included (Table 22):

- education and upskilling programs in critical appraisal, data interpretation, change management, implementation and evaluation through teaching modules, online resources and masterclasses.
- support programs such as problem solving workshops, clinical fellowships and mentoring programs.

The Pharmacy Department and four medication-related committees (Therapeutics, Medication Safety, Adverse Drug Reaction and High Cost Drugs) were chosen to pilot the Capacity Building Service based on their roles in decisions for purchase and/or use of pharmaceuticals and their interest in disinvestment. Staff involved in the SHARE disinvestment projects were also invited to participate [27].

Evaluation immediately after workshops showed participants' knowledge and confidence improved in all aspects of the evidence-based change process and the concepts of EBP, implementation and evaluation. There were further improvements after three months, however there were only a small number of responses. Participants reported high rates of satisfaction and noted that the workshops met or exceeded their expectations [15].

Due to the reduced funding in the final year of the SHARE Program, the service was not expanded beyond the target audience of the pilot and the online resources, fellowships and mentoring program were not established.

Aim 3.4 Project Support Service

Health service staff report that they do not have the necessary skills and frequently have insufficient time and resources to deliver projects effectively [15]. The Project Support Service was established to investigate the nature and amount of guidance and support required to meet the needs of the SHARE disinvestment project teams [27].

Four SHARE disinvestment projects were commenced. It was anticipated that methodological advice and support would be delivered in a range activities related to project planning, governance and administration; implementation and evaluation and practical assistance provided for data capture, entry and analysis (Table 23). One of the clinical project teams required support in all of these areas. The other three were still in the decision-making and development phase and needed assistance in searching the literature, appraising evidence, analysing local data, determining the nature and scope of the problem, clarifying the intervention and assessing feasibility and risk before they were ready to proceed. These projects were subsequently withdrawn based on the outcomes of this process.

Each of the four clinical project teams acknowledged their lack of skills and experience in using evidence in decision-making, project management, implementation and evaluation. They were appreciative that support was available and were willing to accept guidance.

Due to the reduced funding, the fourth project had not completed implementation when the SHARE Program ceased. Although evaluation of project outcomes could not be undertaken as planned, the clinical project team provided feedback on the Project Support Service. Expertise of CCE staff, practical support in development of the evaluation plan and design of a Microsoft Access database, and assistance with data entry and reporting were noted as positive factors.

Table 22. Activities of the Capacity Building Service

Reproduced with permission from SHARE Paper 7 [15]

Training workshops

Interactive workshops to improve knowledge and skills

- Evidence-based change process (½ day)
  - To understand the steps in developing, implementing and evaluating a change process
  - To apply the principles of evidence based practice to each step
  - To outline methods of collecting the information required to develop, implement and evaluate your project using this framework
  - To learn and share practical hints and tips for successful evidence-based change
- Evidence-based practice (4 x ½ day)
  - To understand PICO elements and develop a searchable question
  - To learn the best research design to answer specific questions
  - To learn methods for searching health databases and undertake your own searches
  - To understand the role of chance, bias and confounding
  - To learn methods for critical appraisal and undertake appraisal exercises
- Introduction to implementation (½ day)
  - To understand the principles of evidence-based implementation
  - To learn methods for identifying barriers and enablers and developing implementation strategies
  - To learn and share practical hints and tips for successful evidence-based implementation
  - To design an implementation plan for your project
- Introduction to evaluation (½ day)
  - To understand evaluation: What? Why? When?
  - To understand evaluation frameworks and plans and data collection methods and sources
  - To consider the role of ethics in evaluation
  - To understand Program Logic Models
- Using evidence in decision-making (1½ hours) (planned but not delivered)
  - To consider the deliberation process and the role of decision-making criteria
  - To discuss the principles of evidence-based decision-making (EBDM)
  - To understand the implications of research design, level of evidence, quality, applicability, lack of evidence
  - To apply the learnings in worked examples
  - To be introduced to resources and services that support EBDM

Problem solving/support sessions

Rotating 4 weekly series of open workshops to provide ongoing support to workshop participants undertaking projects.

- Week 1: Finding and appraising evidence and interpreting results
- Week 2: Planning and implementing projects
- Week 3: Evaluating programs and projects
- Week 4: Developing guidelines and protocols

Online resources/teaching (to be sourced or developed)

- Electronic workbook
- PowerPoint presentation/s
- Self-assessment quizzes

Table 23. Activities of the Project Support Service

Reproduced with permission from SHARE Paper 7 [15]

Stage of Project	Activities	Proposed	SHARE projects			
			1	2	3	4
Decision-making and project development	Searching literature			✓	✓	
	Appraisal of evidence			✓	✓	
	Analysis of local data				✓	
	Determination of nature and scope of problem			✓	✓	
	Clarification of the intervention			✓	✓	
	Analysis of feasibility and risk				✓	
Project planning	Confirmation and documentation of scope, objectives, background, etc	✓	✓	✓	✓	✓
	Identification of needs of clinical project team	✓		✓	✓	✓
	Identification of stakeholders	✓				✓
Project management	Confirmation and documentation of governance processes	✓				✓
	Establishment of management and administration systems and processes	✓				✓
Implementation planning	Capture and analysis of barriers and enablers	✓				✓
	Identification of strategies to address barriers and enablers	✓				
	Development of implementation plan (including communication plan)	✓				✓
	Liaison with committees/departments for authorisation of practice change	✓				✓
	Liaison with committees/departments for authorisation of documentation	✓				✓
Evaluation planning	Development of evaluation framework and plan	✓				✓
	Development of costing/economic evaluation plan	✓				✓
	Identification of relevant tools	✓				✓
Development of data collection systems	Liaison with Health Information Management to determine codes	✓				✓
	Liaison with Clinical Information Management to access patient data	✓				✓
	Liaison with data analysts, statistician, health economist, other experts	✓				✓
	Development of data collection tools	✓				✓
	Development of electronic database (eg Access or Excel)	✓				✓
	Training project workers in use of database programs	✓				✓
Evaluation	Assistance with data entry	✓				✓
	Assistance with data cleaning	✓				
	Assistance with data analysis	✓				
Reporting	Development of reporting schedule	✓				✓
	Assistance with reporting	✓				

Aim 4. Program evaluation and research

Aim 4 addresses the lack of information about factors influencing resource allocation, processes for implementing disinvestment decisions, and perspectives and experiences of healthcare staff undertaking disinvestment.

Although each of the first three aims included evaluation in their pilot and implementation phases, a fourth aim was specified to highlight the importance of evaluation, research and dissemination in capturing and understanding what happened and sharing this with others interested in developing similar models.

Aim 4.1 Evaluation and explication

An evaluation framework and plan was developed for the overall SHARE Program and included evaluation domains, audience, scope, evaluation questions, outcomes hierarchy, sources of data, methods of collection and analysis, reporting and timelines [139]. More detailed evaluation plans were developed for individual projects.

Due to the size and complexity of the SHARE Program, its interconnectedness with other Monash Health activities, and the inability to separate out factors that influenced economic outcomes, an economic evaluation of the overall program was not possible. Economic evaluations were planned for the disinvestment pilot projects and support services, but were not undertaken due to the reduction in funding in the final year of the program.

Factors that influenced development, processes and outcomes of individual projects were identified using an existing framework and taxonomy for evaluation and explication of evidence-based innovations [140] which was adapted for use in the SHARE Program (Figure 12) [141].

Figure 12. Four adaptations of a framework for evaluation and explication

These adaptations are based on an existing framework for evaluation of implementation of an evidence-based innovation [140]

1. SHARE Program

Reproduced with permission from SHARE Paper 1 [141].

A taxonomy for this framework is also available in Paper 1.

A. Components

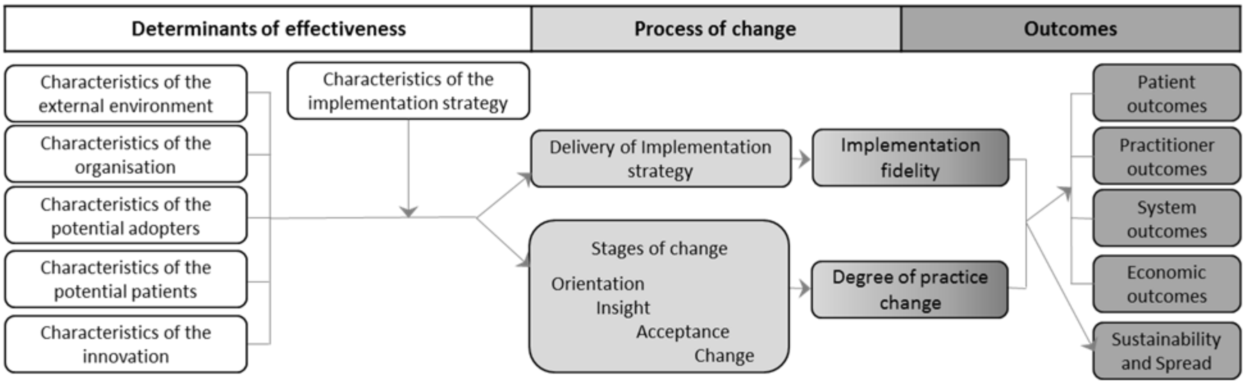

B. Evaluation and research activities for SHARE Program and pilot projects

Determinants of effectiveness	Process of change	Outcomes
Analysis of barriers and enablers	Assessment of perceptions of participants, adopters and patients	Process, impact and outcome evaluation
Documentation of observable characteristics	Detailed documentation of implementation and evaluation process	Assessment of sustainability and spread
Reflective self-evaluation of project team's experience		

2. Investigation of organisational decision-making

Reproduced with permission from SHARE Paper 3 [14]

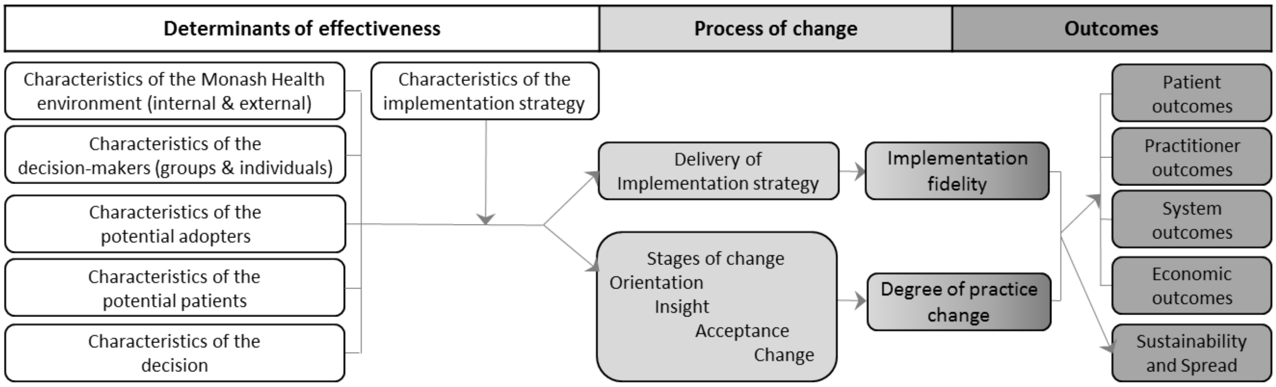

3. Investigation of disinvestment process

Reproduced with permission from SHARE Paper 6 [27]

A taxonomy for this framework is also available in Paper 6.

A. Components

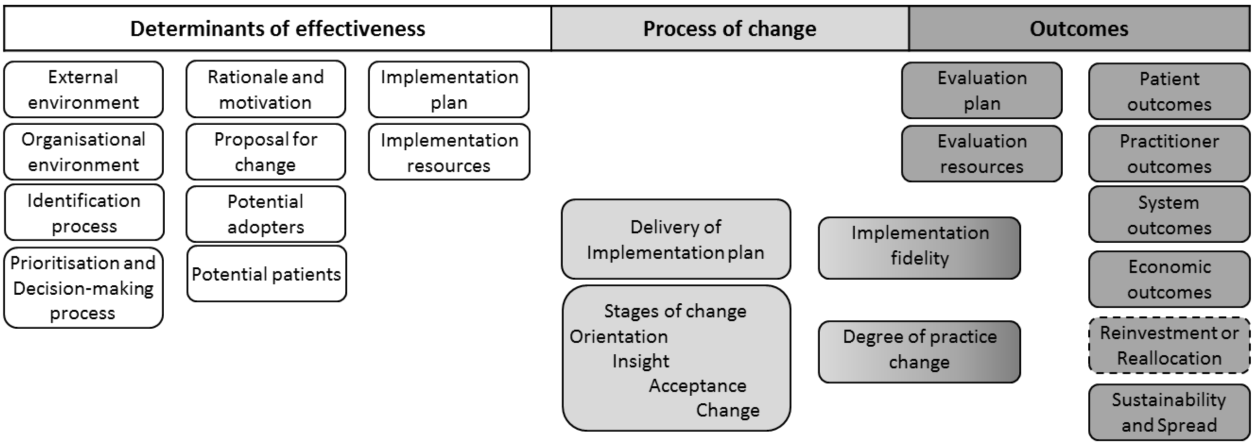

B. Evaluation and research activities for SHARE Program and pilot projects

<table>
<tr><th>Determinants of effectiveness</th><th>Process of change</th><th>Outcomes</th></tr>
<tr><td rowspan="2">Analysis of barriers and enablers</td><td rowspan="2">Assessment of perceptions of participants, adopters and patients</td><td>Process, impact and outcome evaluation</td></tr>
<tr><td>(Assessment of reinvestment or reallocation)</td></tr>
<tr><td>Documentation of observable characteristics</td><td>Detailed documentation of implementation and evaluation process</td><td>Assessment of sustainability and spread</td></tr>
<tr><td colspan="3">Reflective self-evaluation of project team's experience</td></tr>
</table>

4. Investigation of an in-house Evidence Dissemination Service

Reproduced with permission from SHARE Paper 8 [43]

A taxonomy for this framework is also available in Paper 8.

A. Components

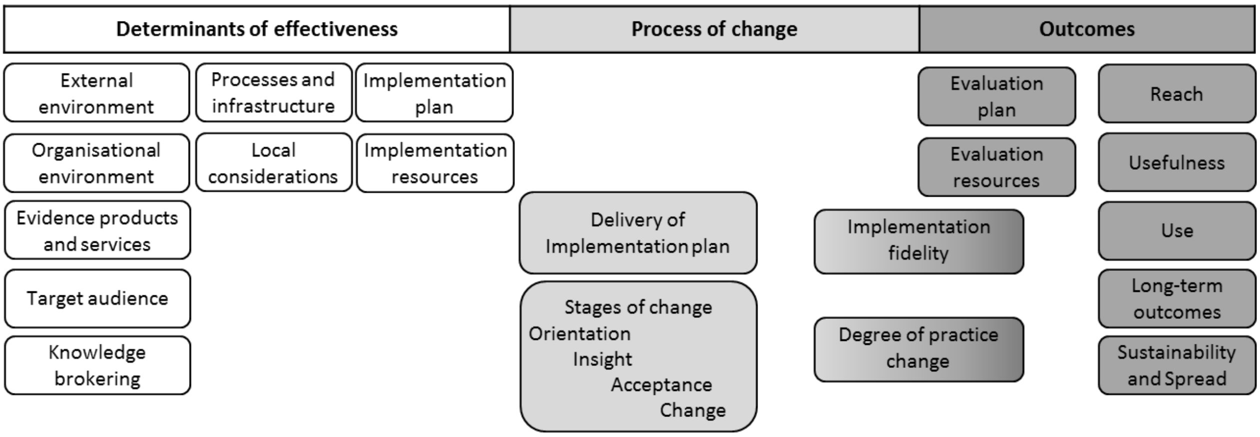

B. Evaluation and research activities for in-house evidence products and services in a local healthcare setting

Determinants of effectiveness	Process of change	Outcomes
Analysis of barriers and enablers	Assessment of perceptions of participants, adopters and patients	Process, impact and outcome evaluation
Documentation of observable characteristics	Detailed documentation of implementation and evaluation process	Assessment of sustainability and spread
Reflective self-evaluation of project team's experience		

Aim 4.2 Action research

Action research was undertaken based on the "*researcher as facilitator for change*" model defined by Meyer: researchers working explicitly with and for people rather than undertaking research on them [142, 143]. In this capacity, CCE staff were both the SHARE project team and the action researchers. An agenda item for 'Learnings' was scheduled at the beginning of every team meeting. Participants were invited to consider anything that had affected the project since the last meeting using the framework 'what worked, what didn't, why and how it could be improved' [116]. Each issue, its effect on the project, and potential changes that would build on positive outcomes or remove or minimise future problems were discussed. The learnings and actions were documented; actions were assigned, given timeframes and followed up to ensure completion. Project team observations and reflections were used for ongoing improvements to the program components, implementation and evaluation processes, and explication of the influencing factors. These methods worked well.

Aim 4.3 National workshop

The first Australian national workshop on disinvestment was conducted to share knowledge and develop links for future collaboration. More than 70 participants attended from Australia and New Zealand representing national and state government departments, health services and providers, academic and research groups, professional associations and consumers. Disinvestment was considered from three perspectives: health policy researchers, health economists and health service decision-makers. All findings and presentation materials were published [144, 145].

Aim 4.4 Dissemination

To address some of the gaps in knowledge and contribute to the understanding of systematic approaches to disinvestment and resource allocation in the local healthcare context, the SHARE Program activities are presented in this thematic series.

PHASE THREE (SHARE Papers 9 & 10)

To achieve this aim of addressing some of the gaps in knowledge and understanding of disinvestment at the local level, a review of the current literature incorporating the SHARE findings was undertaken. This is presented as two papers; the contents of both reviews are listed in Table 24. Paper 9 considers the conceptual elements of disinvestment across four themes that have specific relevance to disinvestment in local healthcare services and proposes a new definition and two potential approaches to disinvestment [146]. Paper 10 presents the literature from an operational perspective in the context of a new framework for disinvestment as a component of resource allocation in the local setting [45].

Some of the findings from the reviews have been integrated into the discussions above; some additional findings particularly relevant to the SHARE Program are summarised briefly below.

Table 24. Contents of the literature overviews

Reproduced with permission from SHARE Paper 9 [146]

Conceptual overview (Paper 9)	Operational overview (Paper 10)
▪ Terminology and concepts<br>– Health technologies<br>– Disinvestment<br>– Resource allocation<br>– Optimising health care<br>– Reinvestment<br>▪ Motivation and purpose<br>– Impetus for disinvestment<br>– Rationale for disinvestment<br>▪ Relationships with other health paradigms<br>– Evidence based health care<br>– Quality improvement<br>– System redesign<br>– Health economic approaches<br>▪ Challenges<br>▪ New approach to disinvestment	▪ Existing theories, frameworks and models<br>▪ New framework<br>– Audience<br>– Application<br>– Definitions<br>– Concepts<br>– Components<br>▪ Principles of decision-making<br>▪ Settings<br>– Decision-making infrastructure<br>– Specific initiatives<br>– Individual decision-makers<br>▪ Prompts and triggers<br>▪ Steps in the disinvestment process<br>▪ Methods and tools<br>▪ Barriers and enablers

Terminology and concepts

There are multiple definitions for the terms ‘disinvestment’ and ‘health technology’, a lack of common understanding of the reasons or objectives that underpin the concepts, and disparity in use of the terms between the research and practice settings (Tables 25 and 26). This creates difficulties in the interpretation of disinvestment, application of research findings, and establishment of a systematic approach in the local healthcare setting.

In the absence of common terminology, there is one notably consistent message: that the word ‘disinvestment’ has negative connotations and is likely to be a barrier to successful implementation of disinvestment-related change. To reduce undesirable effects, other terms have been intentionally introduced to replace ‘disinvestment’ (Table 27) and other concepts have been proposed as alternative, potentially more successful, approaches. For example, ‘resource allocation’ and ‘optimisation of healthcare’ draw the focus away from cost-cutting and redirect it towards effective use of limited resources to maximise health gain.

Table 25. Examples of definitions for disinvestment

Reproduced with permission from SHARE Paper 9 [146]

Definition	Measure	Decision criteria	Position	Action
Disinvestment is an explicit process of taking resources from one service in order to use them for other purposes that are believed to be of better value [35]	Any	Less value than available alternative	Relative	Reallocation
Disinvesting in health interventions that offer no or low health gain (eg are unproven, outdated or cost ineffective) provides an opportunity to invest in alternative proven and cost effective health interventions [147]	Effectiveness, Currency or Cost-effectiveness	Unproven, outdated or cost-ineffective	Absolute	Reallocation
Disinvestment is the process of reducing or ceasing health technologies and clinical practices that provide less favourable outcomes than known alternatives [4]	Any	Less favorable outcome than available alternative	Relative	Removal or Reduction
Disinvestment relates to the withdrawing (partially or completely) of health care practices, procedures, technologies and pharmaceuticals that are deemed to deliver no or low health gain and are thus not efficient or appropriate health resources allocations [82]	Effectiveness	No or low health gain	Absolute	Removal, Reduction or Restriction
Disinvestment can take a number of forms in a healthcare setting…and includes full withdrawal or decommissioning, retraction, restriction and substitution [12]	Any	Unspecified	Unspecified	Removal, Restriction or Replacement
Disinvestment refers to processes by which a health system or service removes technologies, without necessarily replacing them [33]	Any	Unspecified	Unspecified	Removal
Disinvestment relates to the withdrawal of funding from a provider organisation and the subsequent stopping of the service [104]	Any	Unspecified	Unspecified	Defunding (resulting in Removal)
Disinvestment includes the withdrawal or reduction of relatively ineffective healthcare, as well as full withdrawal or rationing of equally worthy alternatives due to resource constraints [10]	▪ Effectiveness<br>▪ Affordability	▪ Relatively ineffective<br>▪ Unspecified	▪ Relative<br>▪ Absolute	Removal,Reductior or Restriction
Disinvestment: the displacement of non–cost-effective technologies for resource reinvestment or reallocation [137]	Cost-effectiveness	Non–cost-effective	Absolute	Reallocation
Disinvestment involves the development & application of epidemiological, economic, ethical & policy appraisals of existing health care interventions that are cost-ineffective or inappropriately applied within health care, leading to displacement of these practices to make way for resource re-allocation towards practices and programs offering greater benefit [148]	▪ Cost-effectiveness<br>▪ Appropriate use	▪ Cost-ineffective<br>▪ Inappropriate use	Absolute	Removal and Reallocation

Table 26. Examples of use of the term 'health technologies'

Reproduced with permission from SHARE Paper 9 [146]

Scope	Definition or use
Definition encompasses all elements across the spectrum of healthcare delivery	"drugs, diagnostic tests, including indicators and reagents, devices, equipment and supplies, medical and surgical procedures, support systems, and organizational and managerial systems used across the spectrum of health care"
Definition based on a selection of elements from the extensive list above	"drugs, devices, procedures and screening" , "drugs, devices and procedures", "pharmaceuticals, devices, diagnostic tests and interventional procedures"
No definition, but wording suggests that health technologies are separate from other elements	"health care practices, procedures, technologies and pharmaceuticals", "health technology, drug or intervention", "health technologies, practices, and procedures"
No definition, but wording suggests that health technologies are products and devices	"purchasing health technologies", "maintenance and repair", "sunk costs and capital infrastructure", "manufacturers and industry stakeholders", "technology lifecycle"

Table 27. Examples of alternatives for the term 'disinvestment'

Reproduced with permission from SHARE Paper 9 [146]

Scope	Alternative terms
Used interchangeably with disinvestment	decommission, remove ineffective services, resource release, defund, ration
Introduced to capture an aspect of disinvestment	health technology reassessment, de-implementation
Proposed to capture the process of disinvestment better	displacement, reallocation, reinvestment
Used to avoid the word disinvestment	prioritisation, reappraisal, reprioritisation, optimisation, substitutional reinvestment, evidence-based reassessment, value for money, therapeutic equivalence, allocative reinvestment, reducing waste, bending the cost curve, contract variation, contract management, service redesign

Motivation and purpose

While definitions and terminology related to disinvestment are common in the literature, the reasons underpinning specific disinvestment activities are not widely discussed although they are likely to affect all aspects of the process from identification and prioritisation through to implementation and evaluation.

Many of the multiple definitions include or imply a reason for disinvestment which can be summarised in seven main themes. An eighth option, 'for any reason', is added for completeness (Table 28 and Figure 13). There is considerable overlap between some themes but others appear to be mutually exclusive. There are many more reasons for removing, reducing or restricting use of TCPs from the perspective of a local healthcare service than those captured in the definitions for disinvestment (Table 29).

Understanding the reason for disinvestment is crucial to project planning. If the objective is to reinvest, the savings need to be measured and explicit decisions about redeployment of the funds are required. However if the purpose is to reduce patient harm or improve health outcomes, the evaluation parameters will be patient measures and there may no savings to reinvest and possibly increased costs to find. The barriers and enablers to implementation and evaluation of these two scenarios are likely to be quite different.

Table 28. Examples of reasons for disinvestment from the literature

Reproduced with permission from SHARE Paper 9 [146]

Objective	Scope
Any reason	This is the broadest sense of disinvestment and refers to cessation or limitation of something that was previously in practice. It could apply to services, programs, use of equipment or clinical interventions. Words used interchangeably with disinvestment in this context are decommissioning, de-implementation, removal, replacement, restriction
To optimise health care	This is also a broad concept. It incorporates investment, disinvestment and reinvestment. The focus is on effective allocation of resources to achieve maximum benefit and combines the concepts of safety, effectiveness, cost-effectiveness and eliminating waste. The approach of 'optimal targeting' is also captured here.
To optimise resource use	A similarly broad concept to optimising health care with considerable overlap of intentions. The difference is in the emphasis on economic outcomes rather than other aspects of health care. This is the objective of Program Budgeting Marginal Analysis (PBMA) and other prioritisation activities.
To improve patient outcomes	This relates to removal of harmful or ineffective practices which result in adverse outcomes for patients and/or replacement with more effective alternatives. The focus is safety and effectiveness but the terms 'low value' and 'of little or no health gain' are also used in this context. There is potential to increase costs rather than save money.
To reduce waste	This could also be thought of as improvement in health service outcomes. From the perspective of disinvestment this primarily addresses inappropriate use of diagnostic tests and therapeutic interventions and failure of care coordination.
To get value for money	This is based on consideration of cost-effectiveness and/or risk-benefit analysis. It may be defined by specifying acceptable cost/QALY ratios or based on local values.
To release resources	This can have two elements: to save money in times of financial constraint or to redirect funds to a preferred alternative. Terms used in this context are cost saving, rationing, priority setting, reinvestment and reallocation. Priority setting exercises may also have this as an objective to use disinvestment to enable investment.
To withdraw funding	The focus of this concept is on the process of disinvestment rather than the reason for doing it. Disinvestment defined in this way refers to the act of withdrawing funding from a provider organisation which results in cessation of a service.

Figure 13. Relationships between reasons for disinvestment

Reproduced with permission from SHARE Paper 9 [146]

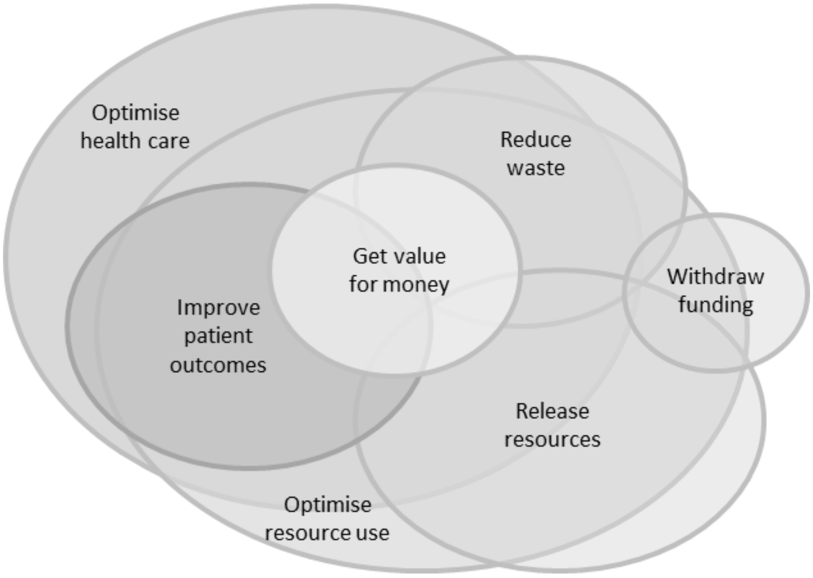

Table 29. Potential reasons for disinvestment in the local healthcare setting

Reproduced with permission from SHARE Paper 9 [146]

**Financial**<br>▪ To save money to meet budget cuts<br>▪ To find money to spend on something else<br>▪ To prioritise where money is spent<br>▪ To redistribute within or between budgets<br>▪ To support investment in new technologies<br>▪ To support continued investment<br>▪ To get value for money	**Patient care**<br>▪ To improve patient health outcomes<br>▪ To reduce patient harm<br>▪ To target populations or indications for best results<br>▪ To improve patient flow and reduce waiting times<br>▪ To improve patient satisfaction or reduce inconvenience<br>▪ To improve patient access and equity of service provision<br>▪ To reduce unnecessary tests or treatment
**Organisational**<br>▪ To meet strategic goals and priorities<br>▪ To ensure sustainability<br>▪ To increase productivity<br>▪ To work within organisational capacity<br>▪ To work within staff capability<br>▪ To rationalise services eg only provide orthopaedics at hospital A and oncology at hospital B<br>▪ To enable system redesign<br>▪ To reduce health service utilisation<br>▪ To reduce risk to staff, finances or reputation<br>▪ To reduce waste<br>▪ To address specific problems	**Health technology, clinical practice or service**<br>▪ To keep equipment up-to-date<br>▪ To remove obsolete or superseded technology<br>▪ To remove or restrict TCPs that are harmful<br>▪ To remove or restrict TCPs that have little or no value<br>▪ To replace TCPs with alternatives of greater benefit<br>▪ To remove services that are not performing as intended<br>▪ To remove services that are not meeting the needs of the target population
**Economic**<br>▪ To maximise benefits from resource use<br>▪ To improve efficiency<br>▪ To maintain quality without extra expenditure<br>▪ To remove TCPs with unacceptable cost per QALY	**External**<br>▪ To address political priorities<br>▪ To meet legislative, regulatory or accreditation requirements and professional standards<br>▪ To meet national recommendations<br>▪ To address legal and ethical issues<br>▪ To be sensitive to the environment
**Evidence Based Practice**<br>▪ To ensure practice is consistent with current evidence<br>▪ To actively identify evidence of harm or lack of effect and remove relevant TCPs<br>▪ To update evidence-based guidelines and protocols	**Social judgement**<br>▪ To ensure public funds are spent wisely<br>▪ To reduce public funding on discretionary services eg some cosmetic procedures

Relationship with other healthcare improvement paradigms

Disinvestment is frequently portrayed as if it is a new paradigm for health improvement. It has been described as an 'emerging field'; disinvestment approaches, processes and initiatives are discussed; 'research agendas' are considered; and the need for mechanisms, frameworks, methods and tools are widely acknowledged. Although there are existing health improvement paradigms that address disinvestment-type activities, these are not routinely promoted in implementation and evaluation of disinvestment. For example, EBP, quality improvement and system redesign all have mature frameworks with validated methods that are widely-used and well-accepted in local health services. It is not clear why there is a need for new methods specific to disinvestment in preference to building on existing familiar processes.

Challenges

The nature of disinvestment brings some particular challenges to achieving change. These include a sense of loss experienced by patients and health professionals; challenges to the clinical expertise of providers; need for more convincing evidence before change is accepted; possibility of benefit of the TCP in some cases; heterogeneity of outcomes that suggest benefits to some groups but not others; lack of data and formal methods for quantifying savings and benefits; lack of standardised methods for decision-making; lack of transparency in disinvestment processes; nomination of disinvestment targets by 'outsiders' who are not directly involved in use of the TCP; lack of clarity and rationale and insufficient information to support disinvestment proposals; and conflicting roles of local decision-makers who wear many 'hats' as advocates for their patients, their department, the health service and the wider population.

Redefining disinvestment

There is little evidence of active and successful implementation of specific 'disinvestment initiatives' in the local healthcare setting and specifically seeking out targets when the expressed aim is 'to disinvest' has not been effective. Yet successful removal, restriction and replacement of technologies, clinical practices, programs and services are commonplace at the health service level. These changes are not called disinvestment and the impetus for change is not 'to disinvest' but to meet more constructive aims such as to improve patient safety, implement evidence-based practices, address changing population needs or redirect resources to more pressing priorities. This suggests that the construct of 'disinvestment' may be problematic in the local healthcare setting. After more than a decade of limited success, it may be time to consider new ways of approaching disinvestment. To stimulate research and debate, we put forward two options that address some of the issues identified [146].

The first proposed that if the concept of 'disinvestment' is to remain as a specific aim and activity, it must be clarified and consolidated from three perspectives: 1) Terminology, to achieve a common understanding of disinvestment between researchers and decision-makers and improve communication in disinvestment initiatives; 2) Research, to define and agree upon theoretical underpinnings, scope and methodologies; and 3) Application, to define and agree upon frameworks, models, methods and tools.

The second proposed that the concept of disinvestment is 1) simplified so that it is not a specific aim or activity, but is the outcome of a resource allocation decision, and 2) assimilated within familiar health improvement paradigms to build on existing knowledge and expertise in the health workforce.

In the second option, the term 'disinvestment' would be used in the broadest sense, effectively the opposite of investment, as 'removal, reduction or restriction of any aspect of the health system for any reason'. This can be applied to products, devices and equipment; clinical practices and procedures; health services and programs; information technology and corporate systems. Unlike most of the research definitions for disinvestment, this version is not constrained by a specified purpose (eg withdrawing practices of low value), defined criteria (eg effectiveness or cost-effectiveness) or anticipated outcome (eg reallocation of resources) which do not address cessation or limitation of TCPs for other purposes, based on other criteria, for different outcomes, which are likely to arise in local health services [146].

In contrast, we propose that 'health technologies' is defined in the narrowest sense, as products, devices and equipment (eg prostheses, implantable devices, vaccines, pharmaceuticals, surgical instruments and diagnostic tools) which reflects common use by health service staff and consumers but excludes clinical practices, health programs and services, support systems, information technologies, and managerial systems which are included in research definitions [45].

Theories, frameworks and models

There is little discussion of the role of theory or theoretical approaches to disinvestment in the literature, however 15 frameworks and models related to disinvestment, resource allocation and priority setting were identified; eight of them from the SHARE Program (Table 30). These are mostly conceptual and untested. They include projects to identify and disinvest individual TCPs, programs for sector-wide investment and disinvestment, evaluation, and stakeholder engagement; but none consider a systematic, integrated, transparent, evidence-based, organisation-wide approach to disinvestment at the local level.

Table 30. Examples of frameworks and models related to disinvestment

Reproduced with permission from SHARE Paper 10 [45]

Framework/Model	Setting	Aims	Method of development	Components
**Projects to identify and disinvest individual TCPs**				
Framework of potential settings and methods for disinvestment [2]	Organisation-wide program in local health service network	To identify potential settings and methods for disinvestment decision-making within local health service systems and processes	Literature review; survey of external experts, interviews and workshops with local stakeholders	Three organisational contexts that provide potential opportunities to introduce disinvestment decisions into health service systems and processes are presented in order of complexity, time to achieve outcomes and resources required: 1. Explicit consideration of potential disinvestment in routine decision-making for purchasing and procurement and development of guidelines and protocols, 2. Proactive decision-making about disinvestment driven by available evidence from published research and local data, 3. Specific exercises in priority setting and system redesign.
Algorithm for selecting a disinvestment project from a catalogue of potential opportunities [27]	Organisation-wide program in local health service network	To facilitate decision-making for identification of potential and selection of actual disinvestment projects	Literature reviews; surveys, interviews and workshops with local stakeholders; document analysis; consultation with experts; taxonomy development	Five steps in selection process: 1. Assess highest risk, 2. Assess importance and potential, 3. Assess quality and strength of evidence, 4. Assess extent of problem, 5. Assess implications of change.<br>Three key decision-making steps between Steps 2 and 3, 3 and 4, and after 5.<br>After selection: Notify decision; Implement; Evaluate; Report<br>Each step includes the activities, who will undertake them, and the decision options
Model for an Evidence Dissemination Service [43]	Organisation-wide program in local health service network	To facilitate use of recently published synthesised evidence in organisational decision-making	Literature reviews; surveys, interviews and workshops with local stakeholders; document analysis; consultation with experts; taxonomy development	Methods and tools to identify sources of high quality synthesised evidence; automate methods of capture; classify, collate and store materials in useful categories; prioritise based on user and health service needs; repackage into suitable formats based on user needs; identify relevant individuals or groups to receive information; disseminate to the appropriate target groups, and report use of evidence
Guideline for Not Funding Health Technologies (GuNFT) [60]	Two versions are provided, one for application at national and regional level and the other at local level.	To facilitate establishment of a transparent, systematic and explicit process for assessing the potential for disinvestment in certain health technologies or in some of their indications	Literature review; face-to-face meeting, teleconference and emails using Nominal Group Technique with ten experts representing health care delivery, administration, technology assessment and consumers to draft the guideline; validation by two external experts in HTA; wide circulation for comment and approval	Seven phases: 1. Identification through applications; 2. Validation of applications; 3. Prioritisation (if necessary); 4. Assessment; 5. Decision making; 6. Development of an action plan; 7. Diffusion of the decision, the reasons why it has been taken and the action plan. Applications are submitted by health care professionals; validation, prioritisation and assessment of the applications are undertaken by a HTA agency or the health service Technology Assessment Committee; and the decision, development of the action plan and diffusion is undertaken by the health service or regional health authority management team or other multidisciplinary body. Tools are available.
Disinvestment framework to guide resource allocation decisions in health service delivery [59]	Health service delivery organisations	To aid disinvestment activity in the local setting.	Thematic analysis of systematic review and a scoping review of the public sector and business literatures. Draft framework critiqued by Decision Maker Advisory Committee (Chief Financial Officers from Canadian health services) and External Reference Group (international academics) before being finalised.	Seven steps: 1. Determine objectives and scope; 2. Identify strategic priorities; 3. Identify options and risk; 4. Rank options; 5. Develop implementation plan; 6. Conduct disinvestment; 7. Assess outcomes and processes. Oversight Committee (senior managers and clinical leaders) is responsible for the majority of the process components including making final decisions; independent Assessment Committee (managers, clinicians, other staff and public representatives) defines the criteria, weights and scale used to assess disinvestment options, Support Committee (researchers and financial personnel) assists in the assessment of disinvestment options in the form of evidence, financial analysis and evaluative measures.

**Programs for sector-wide investment and disinvestment**				
Framework of components in the resource allocation process [14]	Organisation-wide program in local health service network	To represent components in the process of resource allocation and the relationships between them	Interviews and workshops with stakeholders, thematic analysis of responses, document analysis, use of existing frameworks to synthesise findings	Eight components: Governance, Administration, Stakeholder engagement, Resources Decision-Making, Implementation, Evaluation, and, when appropriate, Reinvestment.<br>Details of elements of structure and practice within each component are provided. Structure is described as 'who' and 'what' and includes people, systems, policies, requirements, relationships and coordination. Practice addresses 'how' through processes, procedures, rules, methods, criteria and customs.
Model for Sustainability in Health care by Allocating Resources Effectively (SHARE) [3]	Organisation-wide program in local health service network	To develop, implement and evaluate organisation-wide systematic, transparent, accountable and evidence-based decision-making systems and processes	Three literature reviews; online survey, interviews and structured workshops with stakeholders; consultation with experts in disinvestment, health economics and health program evaluation; drafted in consultation with staff, consumers and external experts; assessed against framework for success and sustainability	Four components, each with multiple elements: 1. Systems and processes; 2. Disinvestment projects; 3. Support services; 4. Program evaluation and research<br>The model outlines each component and the relationships between them, their aims and activities as well as the underlying principles and the preconditions required for success and sustainability. There is also detailed discussion of the antecedents, barriers and enablers.
New Zealand National Health Committee Workplan [61]	National government decision-making	To provide the Minister of Health with recommendations for use and funding of health technologies	Not documented	The program addresses which technologies should be publicly funded, to what level and where technology should be provided and how new technology should be introduced and old technology removed.<br>Six phases: 1. Identification, 2. Prioritisation, 3. Analyse and Assess, 4. Recommend, 5. Implement, 6. Evaluate.
Health technology reassessment and decommissioning framework/model [137]	National or provincial government decision-making	To create a model for assessing the health technology life cycle to identify and delist obsolete technologies	Focused narrative literature review and input from experts.	Two components: 1. Health technology life cycle and reassessment, 2. Reassessment and Decommissioning Model, with Oversight Committee, Triggers, and Possible Outcomes.<br>Second component includes triggers and processes, structure (oversight committee), decisions and outcomes
**Program evaluation**				
Framework for evaluation of priority setting [149]	National, regional and individual healthcare facilities	To develop a framework for the evaluation of priority setting practice at macro and meso levels	Literature review and thematic analysis	Two evaluation domains: 1. Consequentialist outcomes: Efficiency, Equity, Stakeholder satisfaction, Stakeholder understanding, Shifted (reallocation of resources), Implementation of decisions, 2. Proceduralist conditions: Stakeholder engagement, Empowerment, Transparency, Revisions, Use of evidence, Enforcement, Community values
SHARE Program Evaluation Framework and Plan [3]	Organisation-wide program in local health service network	To assess the effectiveness of the SHARE program, implementation fidelity and factors for successful change	Drafts prepared by project team in consultation with Consultant in Health Program Evaluation to meet the information needs of key stakeholders and the internal capacity of staff conducting the project; revised and finalised in consultation with key stakeholders	Seven evaluation domains: 1. Improved patient care, 2. Improved resource allocation for health technologies and clinical practices, 3. Improved decision-making, 4. Improved staff capacity in use of evidence and data in decision-making and implementation of practice change, 5. Barriers and enablers, 6. Implementation fidelity, 7. Sustainability and spread. Includes an outcomes hierarchy based on the SHARE program components and a research program based on a theoretical framework for implementation of an evidence-based innovation.

Framework for evaluation and explication of the processes and outcomes of a disinvestment project [27]	Organisation-wide program in local health service network	To adapt a framework and taxonomy for evaluation of evidence-based innovations to enable evaluation and explication of disinvestment projects	Literature review, surveys and interviews with stakeholders	Three components: 1. Determinants of effectiveness (characteristics of external environment, organisation, proposal for change, rationale and motivation, potential adopters, potential patients, identification process, prioritisation and decision-making process, implementation plan, implementation resources); 2. Process of change (delivery of implementation strategy and stages of change); 3. Outcomes (process and impact for patient, practitioner, systems, economic, reinvestment, sustainability and spread).<br>Taxonomy containing details within each component is provided.
Integrative framework for measuring overuse [150]	Relevant settings within health care systems	To assess the impact of efforts to reduce low-value care.	Not documented	Provides list of measurement tools linked to specific project/program goals and discusses advantages and disadvantages of each approach
**Stakeholder engagement**				
SHARE model for incorporating consumer views into decisions for resource allocation [19]	Organisation-wide program in local health service network	To involve consumers in organisation-wide decision-making, capture their perspectives and incorporate them into decisions for resource allocation.	Literature review, individual and group interviews with Consumer Working Group and health service staff, workshop with Community Advisory Committee, drafting and revision with consumer participation.	Four components: 1. Principles, 2. Scope, 3. Preconditions, 4. Activities<br>Activities include Consumer engagement (communication, consultation and participation) and use of Consumer evidence (consumer perspectives found in publications and data sources). Details of activities are reported in the context of the components of the resource allocation process noted above
New Zealand National Health Committee Workplan [61]	National government decision-making	To seek advice and engage with the health sector	Not documented	Tiered approach to engage with and seek advice from clinicians via colleges and specialty societies; providers such as District Health Boards, NGOs and private facilities via Health Sector Forum; international Health Technology Assessment agencies; Universities and Research Institutes, international and domestic manufacturers.

New framework for an organisation-wide approach to disinvestment in the local healthcare setting

While there is no overarching framework for disinvestment in this context, there are clear and consistent messages in the literature which are used as the basis for a new framework for operationalising disinvestment (Figure 14). The details of each of the framework components are clearly articulated in the literature and many are derived from extensive work with stakeholder groups including decision-makers, policy-makers, health service staff, patients and members of the public.

The framework is proposed as an organisation-wide application, embedded within existing systems and processes, which can be responsive to local needs and priorities, and employed in policy, management or clinical contexts.

It brings together the definitions, concepts, principles, decision-making settings, potential prompts and triggers to consider disinvestment, and steps in the disinvestment process found in the literature. It also seeks to remove barriers when it is possible to do so through establishment of new or adjustment of existing operational mechanisms.

Definitions for essential terms and key concepts underpinning the framework have been made explicit to address the lack of consistent terminology. To avoid the negative connotations of the term 'disinvestment' and the problems inherent in considering disinvestment in isolation, the proposed framework is based on 'resource allocation' to address the spectrum of decision-making from investment to disinvestment.

The framework is composed of three interconnected and interdependent components: 1) a program for organisation-wide decision-making, 2) projects to implement decisions and evaluate outcomes, and 3) research to understand and improve the program and project activities. The program consists of principles for decision-making and settings that provide opportunities to introduce systematic prompts and triggers to initiate disinvestment. The projects follow the steps in the disinvestment process. Each component has a number of elements which are outlined in detail in Paper 10 and summarised in Tables 31-35.

Potential methods and tools are presented and discussed in Paper 10, however the framework does not stipulate project design or conduct; allowing application of any theories, methods or tools at each step. Barriers are discussed and examples illustrating constituent elements are provided (Table 36).

Key findings and recommendations

The key findings and recommendations from the overall SHARE Program are summarised in Table 37.

Figure 14. Framework for an organisation-wide approach to disinvestment in the local healthcare setting

Reproduced with permission from SHARE Paper 10 [45]

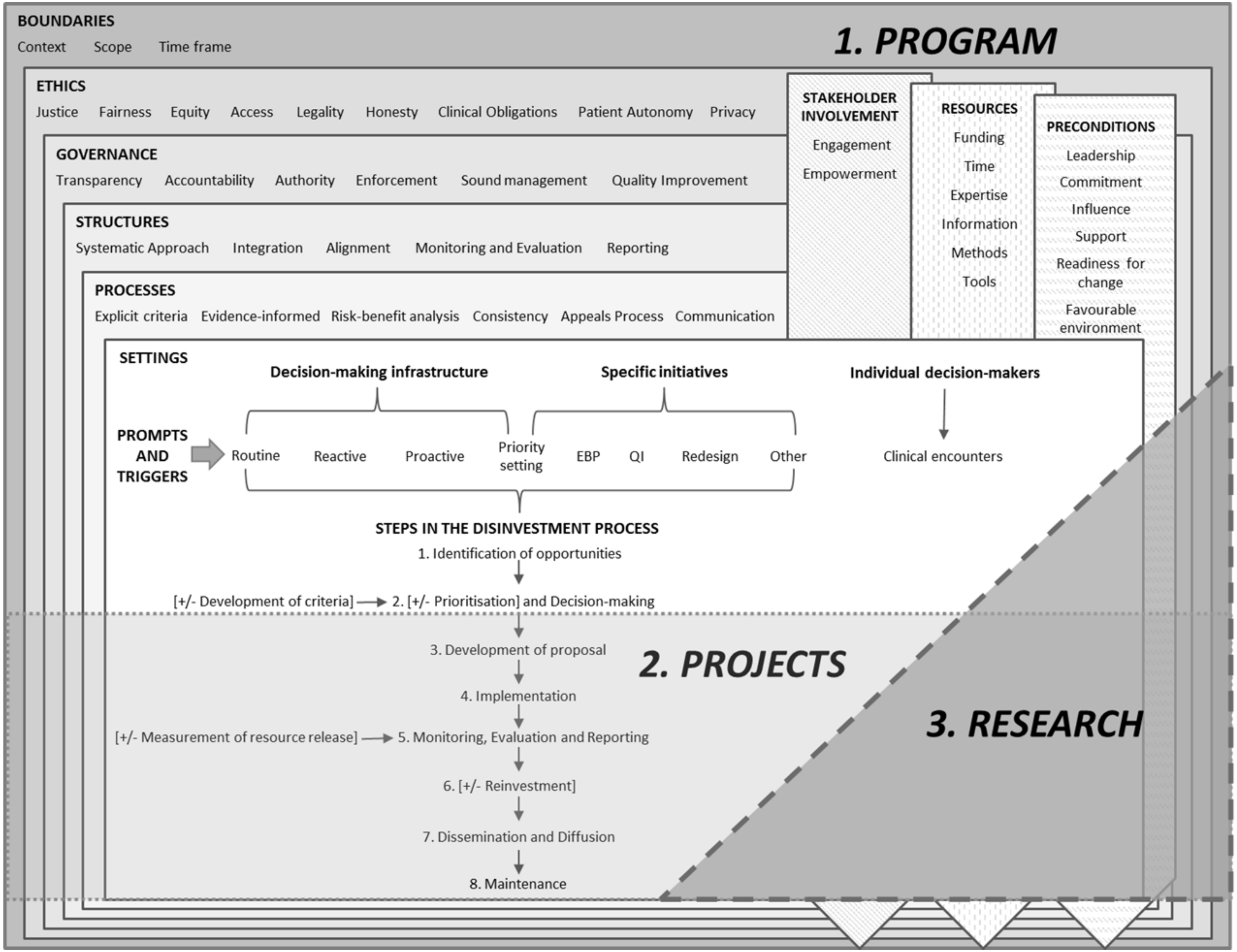

Table 31. Definitions underpinning the framework for an organisation-wide approach to disinvestment
Reproduced with permission from SHARE Paper 10 [45]

Health technologies	Health products, devices and equipment used to deliver health care (eg prostheses, implantable devices, vaccines, pharmaceuticals, surgical instruments, telehealth, interactive IT and diagnostic tools). This is a narrow definition reflecting the common use by health care decision-makers and consumers. Clinical practices, support systems, or organisational and managerial systems are NOT considered to be health technologies in this context.
Health technologies and clinical practices (TCPs)	Therapeutic, preventative and diagnostic procedures (eg use of products, devices and equipment PLUS medical, surgical, nursing, allied health and population health interventions). This is a pragmatic term to reflect the scope of most resource allocation decisions in the local healthcare context.
Health programs and services	Agencies, facilities, institutions and the components within them that deliver health care, rehabilitation or population health practices such as health promotion and education.
Disinvestment	Removal, reduction or restriction of any aspect of the health system for any reason. Removal indicates complete cessation, reduction is a decrease in current volume or delivery sites, and restriction is narrowing of current indications or eligible populations. This is a broad definition, in essence the conceptual opposite of investment. This could apply equally to products, devices and equipment; clinical practices and procedures; health services and programs; information technology and corporate systems.
Principles	Fundamental qualities or elements that represent what is desirable or essential in a system.
Criteria	Standards against which alternatives can be judged in decision-making.
Routine decisions	Decisions made on a recurring basis or scheduled via a timetable eg annual budget setting processes, six-monthly practice audits, monthly Therapeutics Committee meetings, reviews of protocols at specified intervals after their introduction, etc.
Reactive decisions	Decisions made in response to situations as they arise eg new legislation, product alerts and recalls, applications for new drugs to be included in the formulary, critical incidents, emerging problems, etc.
Proactive decisions	Decisions driven by information that was actively sought for the purpose of healthcare improvement eg accessing newly published synthesised research evidence such as Cochrane reviews or Health Technology Assessments to compare against current practice, interrogating routinely-collected datasets to ascertain practices with high costs or high rates of adverse events, etc.
Prompt	An informal reminder or encouragement for thought or action.
Trigger	A formal mechanism that initiates or activates a reaction, process or chain of events.
Diffusion	Passive processes by which an innovation is communicated over time among members of a social system; usually unplanned, informal, untargeted, uncontrolled, decentralised, and largely horizontal or mediated by peers.
Dissemination	Active processes to spread knowledge or research eg publications, presentations and other deliberate strategies; planned, formal, often targeted, controlled or centralised, and likely to occur more through vertical hierarchies.
Maintenance	Active processes to sustain recently implemented change after project support is removed; to integrate the change into organisational systems, processes and practices; and to attain long-term viability of the change.
Methods and tools	Approaches, instruments or other resources that identify 'what' tasks are needed at each step and/or 'how' to undertake them. This is a pragmatic inclusive definition developed for use in this overview to assist health service staff in disinvestment. This broad definition allows frameworks and models to be included if they meet these criteria.

Table 32. Concepts underpinning the framework for an organisation-wide approach to disinvestment
Reproduced with permission from SHARE Paper 10 [45]

<table>
<tr><th>Concept</th><th>Implication for framework</th></tr>
<tr><td>Use of the term disinvestment as a driver or justification for change is associated with negative connotations such as focusing on cost cutting, engendering suspicion and distrust, and getting stakeholders offside.</td><td>Do not use ‘disinvestment’ as the basis for the framework or the aim of change initiatives</td></tr>
<tr><td>Conducting disinvestment activities independently of existing systems and processes does not represent the reality of health service decision-making. It may be counterproductive: lacking incentives for change and introducing disincentives. Disinvestment should not be considered as an isolated activity, but integrated within existing systems and processes in the context of all resource allocation decisions, covering the spectrum from investment to disinvestment.</td><td>Implement disinvestment activities in the context of ‘resource allocation’</td></tr>
<tr><td>Removal or restriction of practices that are harmful or of little or no value; replacement of inferior practices with more effective or cost-effective alternatives; and reduction of organisational waste, systematic error and inappropriate use of TCPs all arise from good policy, management and clinical decisions. If these are based on evidence from research, local data and/or stakeholder views there are sound positive drivers for action. There is no need for the concept of disinvestment to be introduced as a reason for change.</td><td rowspan="4">Focus on the positive reasons driving removal, reduction or restriction of current practices

Use existing systems, processes, expertise, methods and tools whenever possible</td></tr>
<tr><td>It has been proposed that disinvestment activities are more likely to be successful if decisions are transparent, integrated into everyday decision-making and central to local planning rather than ad hoc decisions, individuals ‘championing’ causes or standalone projects</td></tr>
<tr><td>Disinvestment driven from a positive perspective focusing on optimisation of health care through allocation or reallocation of finite resources for maximum effectiveness and efficiency is more likely to be successful.</td></tr>
<tr><td>Existing healthcare improvement paradigms such as Knowledge Translation, Evidence Based Practice, Quality Improvement, System Redesign and Health Economics offer theories, frameworks, methods and tools for decision-making, implementation and evaluation that can be applied to disinvestment.</td></tr>
</table>

Table 33. Principles for a program of decision-making for resource allocation

Reproduced with permission from SHARE Paper 10 [45]

**BOUNDARIES**	
**Context**	Specify the context where decisions will apply. These might include, but are not restricted to, 1) acute, subacute, rehabilitation, community or mental health services; health promotion and education programs; or residential aged care at 2) region, local network, institution, department, ward or committee. [49, 50, 151]
**Scope**	Specify the type of decisions and topics to be addressed. These might include, but are not restricted to, policy, management or clinical decisions to address capital works, plant and equipment; human resources; organisational systems and processes; guidelines and protocols; procurement or commissioning of TCPs, models of care or health programs and services. [144, 152]
**Timeframes**	Specify timeframes for decision-making programs (eg long-term ongoing or defined limited application such as 5 years), implementation of decisions and delivery of outcomes. [3, 10, 49, 53, 59, 147, 153-155]
**ETHICS**	
**Justice**	Maximise outcomes; direct resources for the greatest utility or benefit for the most people, the 'greatest good for the greatest number'. [51, 136, 156-161]
**Fairness**	Act impartially; not discriminating on the basis of race, nationality, colour, language, religion, gender, marital status, sexual orientation, social status, political or other opinion, capacity to pay, location of residence, ownership of property, the need for treatment arising out of past behaviour, or age (except where age may affect the outcome). [44, 51, 53, 69, 71, 72, 104, 159-165]
**Equity**	Horizontal equity: Offer treatment to all patients that meet the relevant criteria, or to none; 'treating like cases alike' or 'equal access for equal clinical need.' The decision should be made for all patients in a group with similar clinical need and not for individuals. Vertical equity: Provide unequal but equitable treatment for people with unequal health needs by giving priority to groups with greater need, for example disadvantage due to social determinants of health. [26, 49, 52, 62, 71, 104, 149, 158-162, 164-168]
**Access**	Ensure consumers or communities are able to use appropriate services determined by five dimensions of accessibility (approachability, acceptability, availability and accommodation, affordability, appropriateness) and five abilities of populations (ability to perceive, seek, reach, pay and engage). [26, 52, 69, 159, 162-164, 169]
**Legality**	Act within the law. Ensure decisions are made by those who are legally accountable for the resources and not made by external groups such as pharmaceutical companies, research bodies, or others with vested interests. [104, 153, 162]
**Honesty**	Be truthful. Do not lie or hide things. [153, 164]
**Clinical obligations**	Guarantee that removal, reduction or replacement of services or TCPs do not compromise clinical ethical obligations, such as beneficence, or other professional standards. [156]
**Patient autonomy**	Empower and encourage patients to make informed decisions about their treatment. Safeguard patient choice and informed consent. [156, 159, 170]
**Privacy**	Ensure patient confidentiality at all times. [156]
**GOVERNANCE**	
**Transparency**	Make all elements clear and visible eg who makes decisions, how decisions are made, reasons for decisions, how they are documented, how they will be implemented and evaluated. Seek declarations of conflict of interest and address them openly. Implement single system ie no parallel system where those who lobby could get undue priority. Record departures from process and subject them to scrutiny. [5, 7-11, 13, 14, 44, 48, 49, 51, 53, 62, 69, 71, 81, 103, 147, 149, 153, 155, 158, 162-165, 167, 170-172]
**Accountability**	Ensure decisions are only made by those who have the authority to do so. Make the lines of authority and responsibility clear and be prepared to acknowledge if errors or complications occur and be accountable for correcting them. [11, 14, 49, 53, 54, 103, 104, 158, 163]
**Authority**	Ensure decision-makers have the knowledge and capability to make the decisions, the control and power to enact them, and the ability to move resources within and between programs, services, facilities, etc as appropriate. [26, 51, 53, 54]
**Enforcement**	Implement mechanisms to ensure firstly that all principles are adhered to and secondly that decisions are enacted as planned. [51, 53, 135, 149, 159, 160, 163, 172-174]
**Sound management**	Establish sound organisational, performance management and resource management structures to ensure due process is followed and implementation of decisions is achieved. Include appropriate corporate expertise from areas such as Finance, Human Resources, Contracting, Communications, Procurement, etc. [3, 10, 13, 26, 53, 59, 62, 104, 153]
**Quality improvement**	Embed opportunities for ongoing reflection on the processes and outcomes of administration of the framework and take the appropriate actions to increase effectiveness, satisfaction and other measures relevant to the stated objectives. [26, 171, 175]

**STRUCTURE**	
**Systematic approach**	Establish systems that are planned, methodical, purposeful and coherent and do not rely on ad hoc, impromptu or improvised mechanisms for decision-making and change. [3, 5, 13, 49, 53, 59, 70, 151, 163, 176, 177]
**Integration**	Incorporate decision-making systems and processes for resource allocation into existing infrastructure and implement system-wide at each level ie region, local network, institution, department, ward or committee. [3, 8, 11, 49, 53, 54, 59, 100, 104, 133, 147, 162, 171, 178]
**Alignment**	Align decision-making systems and processes with the institutional mandate, priorities, strategic goals and objectives. Integrate operational aspects within relevant business plans. [3, 11, 49, 59, 62, 147, 155, 177, 179]
**Monitoring and Evaluation**	Assess compliance with, and effectiveness of, the administration of the program to enable improvement in the systems and processes. Assess outcomes of decisions introducing, removing, reducing or replacing services or TCPs to inform ongoing use and appropriateness of funding. [26, 62, 147, 149, 161, 163, 167, 171, 175]
**Reporting**	Report outcomes of monitoring and evaluation to relevant stakeholders in a transparent and timely manner to enable enforcement and quality improvement and inform future decisions. [11, 26, 153, 161, 180]
**PROCESS**	
**Explicit criteria**	Develop appropriate and achievable criteria to meet the desired objectives, document them explicitly and adhere to them in the decision-making process. [10, 11, 26, 49, 51, 53, 70, 71, 104, 147, 158-160, 163]
**Evidence-informed**	Use the best available evidence for each of the specified criteria. This may include published research or research syntheses (eg systematic reviews, health technology assessments and evidence-based guidelines), population health data, health service utilisation data, cost data, health economic analyses or models, consumer and staff perceptions, or other sources. [3, 5, 8, 10, 11, 29, 33, 50, 51, 59, 62, 70, 71, 81, 104, 133, 149, 153, 155, 157, 159, 163, 164, 167, 177, 179, 181-185]
**Risk-benefit analysis**	Assess the risks and benefits of introducing, continuing, expanding, removing, reducing, restricting or replacing individual services or TCPs. Assess the risks and benefits of implementing a significant change initiative. [158, 162-164, 177, 180]
**Consistency**	Internal consistency: Ensure that the systems, processes, values and reasoning that underpin the program are consistent. In some cases, standardisation may be beneficial.<br>External consistency: Ensure that local programs are consistent with regional programs, regional programs are consistent with national programs, etc.<br>Consistency of information: Ensure that all materials used in communication are consistent with each other and with the systems, processes, values and reasoning of the program.<br>[7, 69, 71, 104, 158, 160, 162-164, 171, 186]
**Appeals process**	Establish formal mechanisms, transparent rules and requirements, to review, revise or appeal decisions. Correct errors and address disagreements constructively. [104, 149, 158, 160, 161, 164, 167, 171]
**Communication**	Document decisions.<br>Develop channels of communication, methods and tools to:<br>▪ Convey information to stakeholders so they are aware of processes, requirements, decisions and actions taken.<br>▪ Seek input from stakeholders to identify issues and drive decisions.<br>▪ Seek feedback from stakeholders to evaluate the processes and outcomes of making and implementing decisions.<br>▪ Ensure ‘top down’ and ‘bottom up’ mechanisms to convey information and seek input and feedback are available, promoted to stakeholders and user-friendly.<br>▪ Distribute information to mass media and social media to educate and inform the community and facilitate public dialogue on healthcare decisions.<br>▪ Share information with the international community to avoid duplication of effort by publishing assessments, decisions, project initiatives and research activities.<br>[8, 19, 62, 69, 147, 153, 155, 158, 161, 171, 177, 180]
**STAKEHOLDER INVOLVEMENT**	
**Engagement**	Identify all relevant stakeholder groups, internal and external to the program. Examples include, but are not restricted to, government departments, local authorities, health agencies, health services, professional associations, representative organisations, advocacy groups, policy makers, managers, health practitioners, researchers, resource personnel (eg systematic reviewers, data analysts, health economists, etc) and representatives of the public. Public participation can involve patients, service users, consumers, community members, citizens, taxpayers, voters, etc. Select an appropriate model, framework or guidance document to follow and use methods and tools for stakeholder engagement relevant to the setting and context.

**Empowerment**	Ensure that stakeholders have the power to contribute to and influence decisions. Implement mechanisms to minimize the effect of the power differences among actors in healthcare organizations; for example give each stakeholder equal opportunities to participate at different stages of the decision-making [149].
**RESOURCES**	
**Funding**	Provide adequate funding to underpin the systems and processes to make, implement and evaluate decisions. [14, 62, 147, 155, 163, 167, 177, 179, 187]
**Time**	Allow all relevant stakeholders to take sufficient time for participation. [48, 50, 53-55, 153, 188]
**Expertise**	Ensure appropriate expertise is available to make, implement and evaluate decisions. Relevant expertise includes, but is not restricted to, finding and using information, health technology assessment, health economics, data analysis and interpretation, negotiation and meeting facilitation, project management, change management, health program evaluation and knowledge and experience in the topic under consideration. [3, 8, 10, 12, 30, 49, 50, 54, 59, 60, 82, 83, 147, 155, 163, 177, 179, 185]
**Information**	Provide adequate and appropriate access to high quality information to underpin decisions including, but not restricted to, research evidence, population health data, local health service data, consumer feedback and economic analyses. [26, 30, 49, 53, 54, 62, 153, 163, 167, 171]
**Methods and tools**	Assist decision-makers, implementers, evaluators and support personnel to find and use appropriate, valid and reliable methods and tools relevant to program and project activities. [3, 8, 10, 12, 30, 49, 50, 54, 59, 60, 82, 83, 147, 163, 185]
**PRECONDITIONS**	
**Leadership**	Appoint and train established and emerging leaders with strengths in negotiation and conciliation, political and cultural awareness and sensitivity. [3, 8, 10, 49, 53, 54, 60-62, 153, 155, 171, 176, 177, 179, 180]
**Commitment**	Establish the program in a way that allows those who are responsible and accountable, the leaders and champions, the decision-makers and support staff to be fully and openly committed, dedicated and loyal to the principles and practices within it. [3, 8, 10, 49, 53, 54, 60-62, 153, 171, 176, 180]
**Influence**	Engage key stakeholders with sufficient and appropriate influence in relevant areas to facilitate and enable rigorous decision-making and effective action. Considerations might include, but are not restricted to, level of seniority, authority, credibility amongst peers, representation on relevant committees, extent of internal and external networks, etc. [3, 26, 149, 155, 177, 179, 188-191]
**Support**	Provide support to those involved by endorsing and promoting decisions, trouble-shooting and problem solving, addressing personal and professional needs, etc. [3, 6, 8, 26, 54, 62, 69, 82, 103, 151, 153, 155, 157, 170, 173, 177, 180, 192-196]
**Readiness for change**	Assess readiness for change at all the relevant levels prior to establishing the program and prior to implementing the decisions taken. Use a valid and reliable instrument. [3, 54, 62, 136, 179, 180]
**Favourable environment**	Consider factors within the internal and external environments that may influence the establishment, delivery and outcomes of the program and what the impacts might be. Examples include, but are not restricted to, setting and context, politics, economic climate, power dynamics and other relationships, priorities, values and culture. [10, 52, 62, 104, 151, 163, 171]
**RESEARCH**	
Consider the role of and opportunities for research in new systems and processes; theories, frameworks and models; methods and tools.	

Table 34. Examples of activities and settings for disinvestment within decision-making infrastructure
Reproduced with permission from SHARE Paper 10 [45]

Activity	Example	Routine	Reactive	Proactive	Priority Setting
Meeting external requirements	▪ Addressing legislative, regulatory and accreditation requirements, national and professional standards, etc	✓	✓		
	▪ Responding to product alerts and recalls		✓		
Setting budgets	▪ Determining sources of income and items of expenditure	✓			✓
Spending money	▪ Introducing new items to funding lists. Examples include, but are not limited to, national health schemes, insurance benefits schedules, institutional lists of permitted TCPs, formularies.	✓	✓	✓	✓
	▪ Commissioning health services and programs	✓	✓	✓	✓
	▪ Procuring capital works, plant and equipment	✓	✓	✓	✓
	▪ Purchasing clinical consumables	✓	✓	✓	✓
	▪ Assessing grant and funding applications	✓	✓		
Allocating non-monetary resources	▪ Allocating people, time, access to facilities, etc	✓	✓	✓	✓
	▪ Developing guidance documents, promotional information or educational materials that indirectly allocate resources. Examples include, but are not limited to, peak body recommendations, clinical guidelines, protocols, standard operating procedures, decision support systems, posters, presentations.	✓	✓	✓	✓
Making strategic and operational decisions	▪ Developing goals and strategies for Strategic Plans	✓			✓
	▪ Developing outcomes measures and targets for Business Plans	✓			✓
Using evidence to initiate and/or inform decisions	▪ Updating existing evidence, undertaking Health Technology Reassessment, etc	✓	✓	✓	
	▪ Accessing and utilising research evidence, population health data, local health service data, consumer and staff feedback	✓	✓	✓	✓
Evaluating outcomes of previous decisions and projects	▪ Monitoring, evaluating and reporting of all newly introduced TCPs to see if they perform as expected, post marketing surveillance	✓			
	▪ Monitoring, evaluating and reporting of purposive or random samples of decisions	✓	✓	✓	
	▪ Monitoring, evaluating and reporting of purposive or random samples of projects	✓	✓	✓	

Table 35. Examples of systematic prompts and triggers to initiate disinvestment decisions
Reproduced with permission from SHARE Paper 10 [45]

Approve introduction or continuation of TCPs for limited time only and require review of desired outcomes, costs, etc before re-approval is granted at end of time period
Approve new guidelines and protocols for limited time only and require review of evidence, costs, etc and appropriate revision before re-approval is granted at end of time period
Include steps that consider disinvestment of existing practices in manuals for guideline and protocol development
Include steps that consider disinvestment of existing practices in checklists for a range of organisational decisions
Add consideration of disinvestment to templates for meeting agendas where appropriate
Mandate consideration of disinvestment in procurement processes: include in requistion documents and require sign off by relevant body overseeing disinvestment at appropriate level
Systematically ascertain evidence from research, data or stakeholder feedback, send directly to decision-makers and seek and/or require response
Incorporate flags and/or question use of low value TCPs in clinical decision support systems
Build questions about potential disinvestment into business case templates and application forms for grants, changes to formulary, introduction of new TCPs, etc
Introduce requirements for consideration of disinvestment into documents governing scope of decisions such as position descriptions and committee Terms of Reference
Add prompts to consider disinvestment to data reports, scorecards, dashboards, etc
Add prompts to consider disinvestment in project management templates and training programs for project management, change management, quality improvement processes, etc
Build disinvestment into strategic planning processes
Build disinvestment KPIs into business plans or performance plans
Consider 'one for one' swaps where a new TCP can only be introduced if an old one is removed

Table 36. Examples of potential barriers to disinvestment

Reproduced with permission from SHARE Paper 10 [45]

Common to all aspects of disinvestment

- Lack of common terminology, theories, tested frameworks and models, proven methods and tools
- The word 'disinvestment' generates negativity and mistrust
- Divergent understanding of the concept of disinvestment between researchers and health service decision-makers
- Lack of guidance and/or successful examples to follow
- Lack of resources particularly time, funds and skills
- Lack of any of the elements of the framework
- Resistance to change

Establishment and delivery of program

- Lack of communication between agencies
- Autonomy of agencies resulting in multiple different systems
- Wastage of resources by duplication of effort, particularly in HTA
- Lack of resources to support policy mechanisms
- Lack of appropriate data collection systems
- Cost of appropriate data collection systems
- Lack of political, clinical, or administrative will to achieve change
- Difficulty establishing systems and processes to assess choices and reallocate resources across and between programs. Easier when done within programs but this has limited effectiveness.
- Difficulty establishing systems and processes between competing sectors or paradigms eg cure versus prevention, acute versus community care, drug therapy versus counselling
- Lack of coordination and integration of systems and processes
- Short-termism in government policy
- Conflicting priorities – at individual levels, and/or between levels
- System inertia
- Longstanding structures, institutional practices and organisational relationships
- Poor understanding of organisational practices and relationships
- Lack of established triggers to initiate disinvestment discussions
- Scarcity of strategic plans that include disinvestment
- Lack of incentives, presence of disincentives
- Fee for service models reward quantity not quality

Stakeholder engagement

- Lack of stakeholder commitment
- Stakeholder inertia
- Difficulty identifying and engaging multiple diverse stakeholders
- Resistance to, or lack of understanding of consumer participation

Identification of disinvestment opportunities

- Health Technology Reassessment (HTR) not conducted routinely
- Public and private funding focused on HTA rather than HTR
- Insufficient 'unequivocal' evidence to disinvest
- Lack of mechanisms to identify disinvestment targets
- Difficulties in producing, accessing & interpreting economic data
- Willingness to use lower quality evidence to maintain status quo

Prioritisation and decision-making

- Lack of knowledge of available tools
- Unfamiliarity with economic evaluations
- Disagreement with assumptions in economic evaluations
- Difficulties estimating marginal costs
- Reluctance to disinvest if there are sunk costs in existing technology and supporting capital infrastructure
- Reluctance to expend effort in disinvestment if benefits not clear
- Gains from disinvestment are less readily measured and may not happen but losses from disinvestment are immediate
- Strength of vested interests and lobby groups
- Lack of negotiating skills making it difficult to resist opposition
- Conflicting priorities between decision-makers
- Conflicting priorities between local, regional and national levels
- Reluctance to disinvest due to heterogeneity of outcomes and/or if there is potential for benefit in some subgroups or individuals
- Controversy associated with removal of an effective TCP in favour of a more cost-effective alternative and/or where there is lack of evidence of effect but general perception that it works
- Sensitivity of disinvestment target eg children, cancer, end of life
- Lack of decision-making processes
- Lack of integration with other decision-making processes
- Requirement for prospective data collection or further research to provide enough information for decision
- Difficulty making choices and reallocating resources across and between programs. Easier when done within programs but this has limited effectiveness.
- Difficulty making choices between competing sectors or paradigms eg cure versus prevention, acute versus community care, drug therapy versus counselling
- Decision-makers not held in sufficiently high regard for decisions to be respected and enforced
- Perceived influence of power imbalances and hidden agendas
- Political challenges

Implementation

- Inadequate project timelines
- Lack of funding for implementation
- Lack of skills in project management
- Lack of skills in change management
- Loss of patient choice
- Loss of perceived entitlement to treatment
- Loss of clinical autonomy
- Clinician reluctance to remove practices they perceive as integral to their professional practice and identity
- Loss of perceived benefit of intervention being removed
- Perceived criticism of practice and/or practitioners
- Perception that management priority is only to save money
- Lack of incentives, presence of disincentives
- Lack of data to substantiate need
- Gains from disinvestment less readily measured and may not happen, but losses from disinvestment are immediate
- Complexity of practice change if disinvestment limited to certain groups or for certain indications
- Lack of coordination between projects resulting in gaps and duplication
- Stakeholder fatigue and disillusionment with constant change

Monitoring and evaluation

- Routinely-collected data not valid or reliable, often out-of-date
- Routinely-collected data not precise or specific enough
- Cost of obtaining appropriate data
- Lack of post-market surveillance
- Lack of methods to quantify savings
- Distrust of reasons for monitoring and evaluation

Reinvestment

- Lack of methods for reallocating resources released
- Lack of examples of successful reinvestment
- Some cost savings may not be realised eg length of stay reduced but beds immediately filled with other patients of greater acuity

Research

- Assumptions that current practice is effective
- Ethical objections to randomising patients to control groups
- Resistance to enrolling patients in trials due to belief in intervention
- Difficulty getting funding to research existing practices

Table 37. Key messages and recommendations
Reproduced with permission from SHARE Paper 11 [197]

**Disinvestment in general**	**Source***
Understanding of systems, processes and influencing factors at the local health service level are important for successful disinvestment.	A
Single definitions for disinvestment and health technologies, are needed with agreement between researchers, policy makers and health service decision-makers [45, 146]. We propose the following definitions.<br>▪ Disinvestment is removal, reduction or restriction of any aspect of the health system for any reason. Removal indicates complete cessation, reduction is a decrease in current volume or delivery sites, and restriction is narrowing of current indications or eligible populations. This is a broad definition, in essence the conceptual opposite of investment. It is an outcome of, rather than a reason for, a resource allocation decision. It is not burdened with the explanations and caveats of current research definitions. This could apply equally to products, devices and equipment; clinical practices and procedures; health services and programs; information technology and corporate systems.<br>▪ Health technologies are products, devices and equipment used to deliver health care (eg prostheses, implantable devices, vaccines, pharmaceuticals, surgical instruments, telehealth, interactive IT and diagnostic tools). This is a narrow definition which reflects the common use by decision-makers and consumers in the local health care setting. Clinical practices, support systems, and organisational and managerial systems are not considered to be health technologies in this context.<br>▪ Health technologies and clinical practices (TCPs) are therapeutic, diagnostic and preventative interventions (eg use of products, devices and equipment PLUS medical, surgical, nursing, allied health and population health activities). This is a pragmatic definition that reflects the scope of most resource allocation decisions related to delivery of health care in the local setting.<br>▪ Health programs and services are agencies, facilities, institutions and the components within them that deliver acute health care, rehabilitation or population health practices such as health promotion and education.	C
Avoid the term 'disinvestment', it is viewed negatively and perceived as 'cost-cutting'. [2, 3, 45, 146]	A
Do not to aim 'to disinvest' [27, 146]<br>▪ TCPs, services and programs that harm patients, diminish health outcomes, impair health care delivery, increase costs unnecessarily or result in organisational waste should be removed, reduced or restricted to address these adverse outcomes.<br>▪ If there are opportunities to replace TCPs, services and programs that are safe, effective and cost-effective with others that offer greater advantage no explanation is needed other than the expected benefit.<br>▪ If budgets are cut or funding is required for high priority activities it is worth remembering that health service staff place a high value on transparency and are disillusioned by attempts to disguise cost reduction methods.	A
Do not develop 'disinvestment' as a health improvement strategy or research domain [27, 146].<br>Expand existing healthcare improvement paradigms and research domains (eg EBP, health technology assessment, guideline development, implementation science, knowledge translation, quality improvement, system redesign, health economics, etc) to address the need for theories, frameworks, methods and tools for [2, 3, 14, 15, 27, 43, 45, 146]:<br>▪ systematic and proactive identification of harmful, ineffective and inefficient TCPs, services and programs<br>▪ implementation of interventions to remove, reduce or restrict TCPs, services and programs<br>▪ evaluation of the process, impact and outcomes of these changes<br>▪ measurement of savings if possible<br>▪ reallocation of resources if appropriate	A
The principles for a rigorous, evidence-based approach to decision-making for disinvestment in the context of all resource allocation decisions are incorporated into the Framework for an organisation-wide approach to disinvestment in the local healthcare setting (Figure 5)	A

**Disinvestment in the local health service setting**	
Decisions to proceed with a project to implement change are often made without consideration of research evidence and local data and are not well-defined in terms of the intervention, practitioner group, patient population, indications, etc.<br>Clinicians are frequently asked to undertake projects in their area of clinical expertise but they lack knowledge and skills in project management, implementation and evaluation.<br>Clinicians are usually required to conduct a project in addition to their normal duties but without additional time or resources.<br>Health service staff are well aware of their limitations and those of their colleagues in undertaking projects and they welcome advice and support.<br>There are many decision-making settings and processes within health services<br>There are many components in the research allocation process in addition to decision-making that need to be addressed<br>Insufficient resources and skills in decision-making, implementation and evaluation<br>Staff need support	A
Decision-making for resource allocation at the local level is not homogenous. Contrary to some assumptions in previous studies, there are multiple layers of decision-making with different actors, criteria, systems and processes. [14]	D
There is a need for proactive methods to access and utilise high quality synthesised evidence in the research literature, routinely-collected local health service data and sources of consumer information to identify and drive disinvestment initiatives [2, 19, 45]	A
Introduce a framework for an organisation-wide approach to disinvestment underpinned by evidence-based principles [45]	A
Focus on optimising health care and using resource effectively rather than cost-cutting	A
Implement systematic, transparent, evidence-based methods that integrate with, or build upon, existing decision-making systems and processes to identify TCPs that should be removed, reduced or restricted. [2, 45]	D
Consider settings for decisions about both monetary (eg capital procurement and clinical purchasing) and non-monetary (eg development and authorisation of guidelines and protocols that stipulate use of drugs or equipment, recommend diagnostic tests, specify referral mechanisms etc) resources as opportunities to identify TCPs that should be removed, reduced or restricted. [2, 3, 27, 45]	D
If seeking opportunities to save money by removing, reducing or restricting TCPs, use a systematic transparent process rather than *ad hoc* nominations from individuals. [27, 146]	A
Ensure that proposals are fully developed before making decisions to proceed including consideration of research evidence and local data to determine the nature and scope of the problem and the most effective solution; clarification of the intervention and scope of the project in terms of practitioner group, patient population, indications, etc; and assessment of feasibility, risk and cost of implementation and evaluation.[15]	D
Integrate activities to remove, reduce or restrict TCPs within the language and methods and tools of familiar health service improvement paradigms such as EBP, quality improvement and system redesign rather than the construct of 'disinvestment'.[14, 45, 146]	A
Include appropriate stakeholder consultation in making, implementing and evaluating decisions to disinvest. [19, 45]	A
Develop mechanisms to receive and act upon consumer or community-initiated feedback on resource allocation decisions. [19]	D
***Key**	

A	Based on findings from literature reviews, and local and/or expert respondents, and outcomes of SHARE investigations
B	Based on findings from literature reviews, and local and/or expert respondents (SHARE investigations incomplete due to local barriers or reduced timelines)
C	Based on findings from literature reviews alone [45, 146] (not investigated in SHARE projects)
D	Based on findings of SHARE investigations alone (not found in other literature)

REFERENCES

1. Campbell NC, Murry E, Darbyshire J, Emery J, Farmer A, Griffiths F et al. Designing and evaluating complex interventions to improve health care. BMJ. 2007;334:455-9.
2. Harris C, Allen K, King R, Ramsey W, Kelly C, Thiagarajan M. Sustainability in Health care by Allocating Resources Effectively (SHARE) 2: Identifying opportunities for disinvestment in a local healthcare setting BMC health services research. 2017. doi:10.1186/s12913-017-2211-6.
3. Harris C, Allen K, Waller C, Green S, King R, Ramsey W et al. Sustainability in Health care by Allocating Resources Effectively (SHARE) 5: Developing a model for evidence-driven resource allocation in the local healthcare setting BMC health services research. 2017. doi:10.1186/s12913-017-2208-1.
4. Rumbold G, Allen K, Harris C. Disinvestment of technologies and clinical practices in health services: Conceptual and policy perspectives. Centre for Clinical Effectiveness, Southern Health 2008. Available from: http://arrow.monash.edu.au/hdl/1959.1/1218935. Accessed: 17/04/2017
5. HealthPACT. Disinvestment in Australia and New Zealand. Health Policy Advisory Committee on Technology 2013. Available from: http://www.health.qld.gov.au/healthpact/docs/papers/workshop/disinvestment-report.pdf. Accessed: 29/09/2017
6. Watt AM, Willis CD, Hodgetts K, Elshaug AG, Hiller JE. Engaging clinicians in evidence-based disinvestment: role and perceptions of evidence. International journal of technology assessment in health care. 2012;28(03):211-9. doi:doi:10.1017/S0266462312000402.
7. Healthcare Improvement Scotland. What approaches have been taken and efforts made to ensure public involvement in decision making relating to potential disinvestment in healthcare interventions and technologies? . In: Public involvement in decision making relating to potential: Technologies scoping report 16 2013. http://www.healthcareimprovementscotland.org/our_work/technologies_and_medicines/shtg_scoping_reports/technologies_scoping_report_16.aspx?theme=mobile. Accessed 29/09/2017.
8. Henshall C, Schuller T, Mardhani-Bayne L. Using health technology assessment to support optimal use of technologies in current practice: the challenge of "disinvestment". International journal of technology assessment in health care. 2012;28(3):203-10. doi:10.1017/s0266462312000372.
9. Rooshenas L, Owen-Smith A, Hollingworth W, Badrinath P, Beynon C, Donovan JL. "I won't call it rationing...": an ethnographic study of healthcare disinvestment in theory and practice. Social science & medicine. 2015;128:273-81. doi:10.1016/j.socscimed.2015.01.020.
10. Robinson S, Williams I, Dickinson H, Freeman T, Rumbold B. Priority-setting and rationing in healthcare: evidence from the English experience. Social science & medicine. 2012;75(12):2386-93. doi:10.1016/j.socscimed.2012.09.014.
11. Rumbold B, Alakeson V, Smith P. Rationing health care. Quest for NHS Efficiency Series 2012. Available from: https://www.nuffieldtrust.org.uk/files/2017-01/rationing-health-care-web-final.pdf. Accessed: 29/09/2017
12. Daniels T, Williams I, Robinson S, Spence K. Tackling disinvestment in health care services. The views of resource allocators in the English NHS. Journal of health organization and management. 2013;27(6):762-80.
13. Garner S, Littlejohns P. Disinvestment from low value clinical interventions: NICEly done? BMJ. 2011;343:d4519. doi:10.1136/bmj.d4519.
14. Harris C, Allen K, Waller C, Brooke V. Sustainability in Health care by Allocating Resources Effectively (SHARE) 3: Examining how resource allocation decisions are made, implemented and evaluated in a local healthcare setting BMC health services research. 2017. doi:10.1186/s12913-017-2207-2.
15. Harris C, Allen K, Waller C, Dyer T, Brooke V, Garrubba M et al. Sustainability in Health care by Allocating Resources Effectively (SHARE) 7: Supporting staff in evidence-based decision-making, implementation and evaluation in a local healthcare setting BMC health services research. 2017. doi:10.1186/s12913-017-2388-8.
16. Solomons NM, Spross JA. Evidence-based practice barriers and facilitators from a continuous quality improvement perspective: an integrative review. Journal of nursing management. 2011;19(1):109-20. doi:10.1111/j.1365-2834.2010.01144.x.
17. Hider PN, Griffin G, Walker M, Coughlan E. The information-seeking behavior of clinical staff in a large health care organization. Journal of the Medical Library Association : JMLA. 2009;97(1):47-50. doi:10.3163/1536-5050.97.1.009.
18. Younger P. Internet-based information-seeking behaviour amongst doctors and nurses: a short review of the literature. Health information and libraries journal. 2010;27(1):2-10. doi:10.1111/j.1471-1842.2010.00883.x.
19. Harris C, Ko H, Waller C, Sloss P, Williams P. Sustainability in Health care by Allocating Resources Effectively (SHARE) 4: Exploring opportunities and methods for consumer engagement in resource allocation in a local healthcare setting BMC health services research. 2017. doi:10.1186/s12913-017-2212-5.
20. Australian Commission on Safety and Quality in Health Care. Safety and Quality Improvement Guide Standard 2: Partnering with Consumers ACSQHC 2012. Available from: http://www.safetyandquality.gov.au/wp-content/uploads/2012/10/Standard2_Oct_2012_WEB.pdf. Accessed: 17/04/2017
21. Consumers' Health Forum of Australia and National Health and Medical Research Council. A Model Framework for Consumer and Community Participation in Health and Medical Research. NHMRC, Australian Government 2004. Available from: https://www.nhmrc.gov.au/_files_nhmrc/file/publications/r33.pdf. Accessed: 17/04/2017

22. Sarrami-Foroushani P, Travaglia J, Debono D, Braithwaite J. Key concepts in consumer and community engagement: a scoping meta-review. BMC health services research. 2014;14:250. doi:10.1186/1472-6963-14-250.
23. O'Mara-Eves A, Brunton G, McDaid D, Oliver S, Kavanagh J, Jamal F et al. Community engagement to reduce inequalities in health: a systematic review, meta-analysis and economic analysis. Public Health Res. 2013;1(4). doi:10.3310/phr01040.
24. Paediatric Integrated Cancer Service in collaboration with the Australian and New Zealand Children's Haematology Oncology Group. A Toolkit for Consumer Participation and Engagement. 2013. Available from: https://pics.org.au/wp-content/uploads/2016/09/Toolkit_CPE_Final_CompleteDocument_24052013.pdf. Accessed: 17/04/2017
25. Department of Health. Doing it with us not for us: Strategic direction 2010–13. Participation in your health service system: Victorian consumers, carers, and community working together with their health services and the Department of Health. Hospital and Health Service Performance Division, Victorian Government 2010. Available from: http://docs.health.vic.gov.au/docs/doc/A6FECA5B2FFB4503CA2578B500229CD0/$FILE/1104007_DIWUNFU_StratDirection_FA3_web.pdf. Accessed: 17/04/2017
26. Harris C, Garrubba M, Allen K, King R, Kelly C, Thiagarajan M et al. Development, implementation and evaluation of an evidence-based program for introduction of new health technologies and clinical practices in a local healthcare setting. BMC health services research. 2015;15(1):575. doi:10.1186/s12913-015-1178-4.
27. Harris C, Allen K, Brooke V, Dyer T, Waller C, King R et al. Sustainability in Health care by Allocating Resources Effectively (SHARE) 6: Investigating methods to identify, prioritise, implement and evaluate disinvestment projects in a local healthcare setting. BMC health services research. 2017. doi:10.1186/s12913-017-2269-1.
28. Bennett CC. Are we there yet? A journey of health reform in Australia. The Medical journal of Australia. 2013;199(4):251-5.
29. Mitchell MD, Williams K, Brennan PJ, Umscheid CA. Integrating local data into hospital-based healthcare technology assessment: two case studies. International journal of technology assessment in health care. 2010;26(3):294-300. doi:10.1017/s0266462310000334.
30. Rubinstein A, Belizan M, Discacciati V. Are economic evaluations and health technology assessments increasingly demanded in times of rationing health services? The case of the Argentine financial crisis. International journal of technology assessment in health care. 2007;23(2):169-76. doi:10.1017/s0266462307070274.
31. Centre for Clinical Effectiveness. Guide to implementation of health service protocols, procedures and guidelines. Southern Health 2010. Available from: http://arrow.monash.edu.au/hdl/1959.1/1225381. Accessed: November 2015
32. Chamberlain CA, Martin RM, Busby J, Gilbert R, Cahill DJ, Hollingworth W. Trends in procedures for infertility and caesarean sections: was NICE disinvestment guidance implemented? NICE recommendation reminders. BMC public health. 2013;13:112. doi:10.1186/1471-2458-13-112.
33. Haas M, Hall J, Viney R, Gallego G. Breaking up is hard to do: why disinvestment in medical technology is harder than investment. Australian health review : a publication of the Australian Hospital Association. 2012;36(2):148-52. doi:10.1071/AH11032.
34. Owens DK, Qaseem A, Chou R, Shekelle P. High-value, cost-conscious health care: concepts for clinicians to evaluate the benefits, harms, and costs of medical interventions. Annals of internal medicine. 2011;154(3):174-80. doi:10.7326/0003-4819-154-3-201102010-00007.
35. Pearson S, Littlejohns P. Reallocating resources: how should the National Institute for Health and Clinical Excellence guide disinvestment efforts in the National Health Service? Journal of Health Services & Research Policy. 2007;12(3):160-5.
36. Cochrane Library. http://www.cochranelibrary.com/. Accessed 24/10/2017.
37. International Network of Agencies for Health Technology Assessment (INAHTA). http://www.inahta.org/. Accessed August 2015.
38. National Institute for Health and Care Excellence (UK). https://www.nice.org.uk/. Accessed August 2015.
39. National Prescribing Service (Australia). http://www.nps.org.au/. Accessed August 2015.
40. Australia and New Zealand Horizon Scanning Network http://www.horizonscanning.gov.au/. Accessed August 2015.
41. National Institute of Health and Clinical Excellence. 'Do Not Do' Recommendations. National Institute of Health and Clinical Excellence, UK. http://www.nice.org.uk/savingsAndProductivity/collection?page=1&pageSize=2000&type=Do%20not%20do&published=&impact=Unclassified&filter=. Accessed 17/04/2017.
42. Jones C. Procedures not routinely funded. Report to the Board, South Birmingham Primary Care Trust. Birmingham, UK, 2006.
43. Harris C, Garrubba M, Melder A, Voutier C, Waller C, King R et al. Sustainability in Health care by Allocating Resources Effectively (SHARE) 8: Developing, implementing and evaluating an Evidence Dissemination Service in a local healthcare setting. BMC health services research. 2017;in press.
44. Elshaug AG, McWilliams J, Landon BE. The value of low-value lists. JAMA. 2013;309(8):775-6. doi:10.1001/jama.2013.828.
45. Harris C, Green S, Elshaug AG. Sustainability in Health care by Allocating Resources Effectively (SHARE) 10: Operationalising disinvestment in an evidence-based framework for resource allocation BMC health services research. 2017. doi:10.1186/s12913-017-2506-7.

46. Gallego G, Haas M, Hall J, Viney R. Reducing the use of ineffective health care interventions: an Evidence Check rapid review brokered by the Sax Institute for the NSW Treasury 2010. Available from: https://www.saxinstitute.org.au/wp-content/uploads/21_Reducing-the-use-of-ineffective-health-care-interventions.pdf. Accessed: August 2015
47. Duckett S, Breadon P, Romanes D, Fennessy P, Nolan J. Questionable care: Stopping ineffective treatments. Grattan Institute 2015. Available from: http://grattan.edu.au/wp-content/uploads/2015/08/828-Questionable-Care3.pdf. Accessed: 13/06/2017
48. Hollingworth W, Rooshenas L, Busby J, Hine CE, Badrinath P, Whiting PF et al. Using clinical practice variations as a method for commissioners and clinicians to identify and prioritise opportunities for disinvestment in health care: a cross-sectional study, systematic reviews and qualitative study. Southampton UK: Queen's Printer and Controller of HMSO 2015; 2015.
49. Marks L, Weatherly H, Mason A. Prioritizing investment in public health and health equity: what can commissioners do? Public health. 2013;127(5):410-8. doi:10.1016/j.puhe.2013.01.027.
50. Polisena J, Clifford T, Elshaug AG, Mitton C, Russell E, Skidmore B. Case studies that illustrate disinvestment and resource allocation decision-making processes in health care: A systematic review. International journal of technology assessment in health care. 2013;29(2):174-84. doi:10.1017/s0266462313000068.
51. Rubenfeld GD. Cost-effective critical care: cost containment and rationing. Seminars in respiratory and critical care medicine. 2012;33(4):413-20. doi:10.1055/s-0032-1322411.
52. Hauck K, Smith P, Goddard M. The Economics of Priority Setting for Health Care: A Literature Review. Health, Nutrition and Population Discussion Paper World Bank 2004. Available from: http://siteresources.worldbank.org/HEALTHNUTRITIONANDPOPULATION/Resources/281627-1095698140167/Chapter3Final.pdf. Accessed: 17/04/2017
53. Mitton C, Dionne F, Donaldson C. Managing healthcare budgets in times of austerity: the role of program budgeting and marginal analysis. Applied health economics and health policy. 2014;12(2):95-102. doi:10.1007/s40258-013-0074-5.
54. Cornelissen E, Mitton C, Davidson A, Reid RC, Hole R, Visockas AM et al. Changing priority setting practice: The role of implementation in practice change. Health policy (Amsterdam, Netherlands). 2014. doi:10.1016/j.healthpol.2014.04.010.
55. Donaldson C, Bate A, Mitton C, Dionne F, Ruta D. Rational disinvestment. QJM. 2010;103(10):801-7. doi:10.1093/qjmed/hcq086.
56. Martin L, Neumann C, Mountford J, Bisognano M, Nolan T. Increasing Efficiency and Enhancing Value in Health Care: Ways to Achieve Savings in Operating Costs per Year. IHI Innovation. Innovation Series 2009 Institute for Healthcare Improvement 2009. Available from: http://www.imagefirst.com/Portals/276565/docs/856157170.pdf. Accessed: June 2015
57. Nolan T. Execution of Strategic Improvement Initiatives to Produce System-Level Results. Innovation Series 2007 Institute for Healthcare Improvement 2007. Available from: http://www.ihi.org/resources/Pages/IHIWhitePapers/ExecutionofStrategicImprovementInitiativesWhitePaper.aspx. Accessed: June 2015
58. Gabow P, Eisert S, Karkhanis A, Knight A, Dickson P. A Toolkit for Redesign in Health Care AHRQ Publication No 05-0108-EF Agency for Healthcare Research and Quality 2005. Available from: http://archive.ahrq.gov/professionals/quality-patient-safety/patient-safety-resources/resources/toolkit/toolkit.pdf. Accessed: June 2015
59. Schmidt DE. The development of a disinvestment framework to guide resource allocation decisions in health service delivery organizations. The University of British Columbia 2010. Available from: https://open.library.ubc.ca/cIRcle/collections/ubctheses/24/items/1.0073252. Accessed: 24/10/2017
60. Ibargoyen-Roteta N, Gutierrez-Ibarluzea I, Asua J. Guiding the process of health technology disinvestment. Health policy (Amsterdam, Netherlands). 2010;98(2-3):218-26. doi:10.1016/j.healthpol.2010.06.018.
61. National Health Committee NZ. Business Plan 2013/14-2015/16. Wellington: New Zealand Ministry of Health, 2013
62. Robert G, Harlock J, Williams I. Disentangling rhetoric and reality: an international Delphi study of factors and processes that facilitate the successful implementation of decisions to decommission healthcare services. Implementation science : IS. 2014;9(1):123. doi:10.1186/s13012-014-0123-y.
63. Leggat SG, Scheil W, Williams H, Kerin K. Tools for priority setting: lessons from South Australia. Australian Health Review. 2006;30(1):65-72.
64. Leggat S. Developing a clinical priority setting framework. South Australian Department of Health, Adelaide & La Trobe University, School of Public Health, Melbourne 2004. Available from: http://www.health.vic.gov.au/archive/archive2010/ambulatorycare/ciyctrial/downloads/developing_clinical_priority_setting_framework.pdf. Accessed: June 2015
65. National Institute of Health and Clinical Excellence. Guide to the multiple technology appraisal process. 2009. Available from: https://www.nice.org.uk/Media/Default/About/what-we-do/NICE-guidance/NICE-technology-appraisals/Guide-to-the-multiple-technology-appraisal-process.pdf. Accessed: 05/05/2017
66. Golan O, Hansen P, Kaplan G, Tal O. Health technology prioritization: which criteria for prioritizing new technologies and what are their relative weights? Health policy (Amsterdam, Netherlands). 2011;102(2-3):126-35. doi:10.1016/j.healthpol.2010.10.012.

67. Noorani HZ, Husereau DR, Boudreau R, Skidmore B. Priority setting for health technology assessments: a systematic review of current practical approaches. International journal of technology assessment in health care. 2007;23(3):310-5. doi:10.1017.s026646230707050x.
68. Sabik LM, Lie RK. Priority setting in health care: Lessons from the experiences of eight countries. International journal for equity in health. 2008;7:4. doi:10.1186/1475-9276-7-4.
69. Ibargoyen-Roteta N, Gutiérrez-Ibarluzea I, Asua J. Report on the development of the GuNFT Guideline. Guideline for Not Funding existing health Technologies in health care systems. Quality Plan for the NHS of the MHSP: Basque Office for Health Technology Assessment (Osteba), 2009.
70. Elshaug A, Moss J, Littlejohns P, Karnon J, Merlin T, Hiller J. Identifying existing health care services that do not provide value for money. Medical Journal of Australia. 2009;190(5):269-73.
71. Guindo LA, Wagner M, Baltussen R, Rindress D, van Til J, Kind P et al. From efficacy to equity: Literature review of decision criteria for resource allocation and healthcare decisionmaking. Cost effectiveness and resource allocation : C/E. 2012;10(1):9. doi:10.1186/1478-7547-10-9.
72. Tromp N, Baltussen R. Mapping of multiple criteria for priority setting of health interventions: an aid for decision makers. BMC health services research. 2012;12:454. doi:10.1186/1472-6963-12-454.
73. Tanios N, Wagner M, Tony M, Baltussen R, van Til J, Rindress D et al. Which criteria are considered in healthcare decisions? Insights from an international survey of policy and clinical decision makers. International journal of technology assessment in health care. 2013;29(04):456-65. doi:doi:10.1017/S0266462313000573.
74. Golan O, Hansen P. Which health technologies should be funded? A prioritization framework based explicitly on value for money. Israel journal of health policy research. 2012;1(1):44. doi:10.1186/2045-4015-1-44.
75. Wallace J, Byrne C, Clarke M. Improving the uptake of systematic reviews: a systematic review of intervention effectiveness and relevance. BMJ open. 2014;4(10):e005834. doi:10.1136/bmjopen-2014-005834.
76. Ellen ME, Leon G, Bouchard G, Ouimet M, Grimshaw JM, Lavis JN. Barriers, facilitators and views about next steps to implementing supports for evidence-informed decision-making in health systems: a qualitative study. Implementation science : IS. 2014;9(1):179. doi:10.1186/s13012-014-0179-8.
77. Oliver K, Innvar S, Lorenc T, Woodman J, Thomas J. A systematic review of barriers to and facilitators of the use of evidence by policymakers. BMC health services research. 2014;14(1):2. doi:10.1186/1472-6963-14-2.
78. Nutley T, Reynolds HW. Improving the use of health data for health system strengthening. Global health action. 2013;6:20001. doi:10.3402/gha.v6i0.20001.
79. Gagliardi AR, Majewski C, Victor JC, Baker GR. Quality improvement capacity: a survey of hospital quality managers. Quality & safety in health care. 2010;19(1):27-30. doi:10.1136/qshc.2008.029967.
80. Tricco AC, Cardoso R, Thomas SM, Motiwala S, Sullivan S, Kealey MR et al. Barriers and facilitators to uptake of systematic reviews by policy makers and health care managers: a scoping review. Implementation science : IS. 2016;11(1):4. doi:10.1186/s13012-016-0370-1.
81. Gerdvilaite J, Nachtnebel A. Disinvestment: overview of disinvestment experiences and challenges in selected countries. HTA-Projektbericht., vol Nr. 57. Vienna: Ludwig Boltzmann Institut für Health Technology Assessment; 2011.
82. Elshaug AG, Hiller JE, Tunis SR, Moss JR. Challenges in Australian policy processes for disinvestment from existing, ineffective health care practices. Australia and New Zealand health policy. 2007;4:23. doi:10.1186/1743-8462-4-23.
83. Williams I, McIver S, Moore D, Bryan S. The use of economic evaluations in NHS decision making: A review and empirical investigation. Health Technology Assessment 2008;12(7).
84. Bowers B, Cohen LW, Elliot AE, Grabowski DC, Fishman NW, Sharkey SS et al. Creating and supporting a mixed methods health services research team. Health services research. 2013;48(6 Pt 2):2157-80. doi:10.1111/1475-6773.12118.
85. van Panhuis WG, Paul P, Emerson C, Grefenstette J, Wilder R, Herbst AJ et al. A systematic review of barriers to data sharing in public health. BMC public health. 2014;14:1144. doi:10.1186/1471-2458-14-1144.
86. Clarke MA, Belden JL, Koopman RJ, Steege LM, Moore JL, Canfield SM et al. Information needs and information-seeking behaviour analysis of primary care physicians and nurses: a literature review. Health information and libraries journal. 2013;30(3):178-90. doi:10.1111/hir.12036.
87. Davies K, Harrison J. The information-seeking behaviour of doctors: a review of the evidence. Health information and libraries journal. 2007;24(2):78-94. doi:10.1111/j.1471-1842.2007.00713.x.
88. Nail-Chiwetalu B, Bernstein Ratner N. An assessment of the information-seeking abilities and needs of practicing speech-language pathologists. Journal of the Medical Library Association : JMLA. 2007;95(2):182-8, e56-7. doi:10.3163/1536-5050.95.2.182.
89. Dawes M, Sampson U. Knowledge management in clinical practice: a systematic review of information seeking behavior in physicians. International journal of medical informatics. 2003;71(1):9-15.
90. Dobbins M, DeCorby K, Twiddy T. A knowledge transfer strategy for public health decision makers. Worldviews on evidence-based nursing / Sigma Theta Tau International, Honor Society of Nursing. 2004;1(2):120-8. doi:10.1111/j.1741-6787.2004.t01-1-04009.x.

91. Bowen S, Erickson T, Martens PJ, Crockett S. More than "using research": the real challenges in promoting evidence-informed decision-making. Healthcare Policy. 2009;4(3):87-102.
92. Revere D, Turner AM, Madhavan A, Rambo N, Bugni PF, Kimball A et al. Understanding the information needs of public health practitioners: a literature review to inform design of an interactive digital knowledge management system. Journal of biomedical informatics. 2007;40(4):410-21. doi:10.1016/j.jbi.2006.12.008.
93. Niedzwiedzka BM. Barriers to evidence-based decision making among Polish healthcare managers. Health services management research : an official journal of the Association of University Programs in Health Administration / HSMC, AUPHA. 2003;16(2):106-15. doi:10.1258/095148403321591429.
94. Gilman IP. Evidence-based information-seeking behaviors of occupational therapists: a survey of recent graduates. Journal of the Medical Library Association : JMLA. 2011;99(4):307-10.
95. Braa J, Heywood A, Sahay S. Improving quality and use of data through data-use workshops: Zanzibar, United Republic of Tanzania. Bulletin of the World Health Organization. 2012;90(5):379-84. doi:10.2471/blt.11.099580.
96. Finch E, Cornwell P, Ward EC, McPhail SM. Factors influencing research engagement: research interest, confidence and experience in an Australian speech-language pathology workforce. BMC health services research. 2013;13:144.
97. Prendiville TW, Saunders J, Fitzsimons J. The information-seeking behaviour of paediatricians accessing web-based resources. Archives of disease in childhood. 2009;94(8):633-5. doi:10.1136/adc.2008.149278.
98. Vest JR, Kirk HM, Issel LM. Quality and integration of public health information systems: A systematic review focused on immunization and vital records systems. Online journal of public health informatics. 2012;4(2). doi:10.5210/ojphi.v4i2.4198.
99. Jorm L. Routinely collected data as a strategic resource for research: priorities for methods and workforce. Public health research & practice. 2015;25(4):e2541540. doi:10.17061/phrp2541540.
100. Evans BA, Snooks H, Howson H, Davies M. How hard can it be to include research evidence and evaluation in local health policy implementation? Results from a mixed methods study. Implementation science : IS. 2013;8:17. doi:10.1186/1748-5908-8-17.
101. Stansfield SK, Walsh J, Prata N, Evans T. Information to Improve Decision Making for Health. In: Jamison DT, Breman JG, Measham AR, Alleyne G, Claeson M, Evans DB et al., editors. Disease Control Priorities in Developing Countries. Washington DC: The International Bank for Reconstruction and Development/The World Bank Group; 2006.
102. Gifford W, Davies B, Edwards N, Griffin P, Lybanon V. Managerial leadership for nurses' use of research evidence: an integrative review of the literature. Worldviews on evidence-based nursing / Sigma Theta Tau International, Honor Society of Nursing. 2007;4(3):126-45. doi:10.1111/j.1741-6787.2007.00095.x.
103. Watt AM, Hiller JE, Braunack-Mayer AJ, Moss JR, Buchan H, Wale J et al. The ASTUTE Health study protocol: deliberative stakeholder engagements to inform implementation approaches to healthcare disinvestment. Implementation science : IS. 2012;7:101. doi:10.1186/1748-5908-7-101.
104. Hughes E, McKenny K. Decommissioning and Disinvestment Toolkit 2013-2014 Rayleigh, Essex, UK: Castle Point and Rochford Clinical Commissioning Group, 2013.
105. Morrato EH, Elias M, Gericke CA. Using population-based routine data for evidence-based health policy decisions: lessons from three examples of setting and evaluating national health policy in Australia, the UK and the USA. Journal of public health (Oxford, England). 2007;29(4):463-71. doi:10.1093/pubmed/fdm065.
106. Kosteniuk JG, Morgan DG, D'Arcy CK. Use and perceptions of information among family physicians: sources considered accessible, relevant, and reliable. Journal of the Medical Library Association : JMLA. 2013;101(1):32-7. doi:10.3163/1536-5050.101.1.006.
107. Cilenti D, Brownson RC, Umble K, Erwin PC, Summers R. Information-seeking behaviors and other factors contributing to successful implementation of evidence-based practices in local health departments. Journal of public health management and practice : JPHMP. 2012;18(6):571-6. doi:10.1097/PHH.0b013e31825ce8e2.
108. Riley BL, Robinson KL, Gamble J, Finegood DT, Sheppard D, Penney TL et al. Knowledge to action for solving complex problems: insights from a review of nine international cases. Health promotion and chronic disease prevention in Canada. 2015;35(3):47-53.
109. Giles-Corti B, Sallis JF, Sugiyama T, Frank LD, Lowe M, Owen N. Translating active living research into policy and practice: One important pathway to chronic disease prevention. Journal of public health policy. 2015;36(2):231-43. doi:10.1057/jphp.2014.53.
110. Murthy L, Shepperd S, Clarke MJ, Garner SE, Lavis JN, Perrier L et al. Interventions to improve the use of systematic reviews in decision-making by health system managers, policy makers and clinicians. Cochrane database of systematic reviews (Online). 2012;9:CD009401. doi:10.1002/14651858.CD009401.pub2.
111. Wallace J, Byrne C, Clarke M. Making evidence more wanted: a systematic review of facilitators to enhance the uptake of evidence from systematic reviews and meta-analyses. International journal of evidence-based healthcare. 2012;10(4):338-46. doi:10.1111/j.1744-1609.2012.00288.x.
112. LaRocca R, Yost J, Dobbins M, Ciliska D, Butt M. The effectiveness of knowledge translation strategies used in public health: a systematic review. BMC public health. 2012;12:751. doi:10.1186/1471-2458-12-751.

113. Moore G, Redman S, Haines M, Todd A. What works to increase the use of research in population health policy and programmes: a review. Evidence and Policy: A Journal of Research, Debate and Practice. 2011;7(3):277-305. doi:10.1332/174426411X579199.
114. Perrier L, Mrklas K, Lavis JN, Straus SE. Interventions encouraging the use of systematic reviews by health policymakers and managers: a systematic review. Implementation science : IS. 2011;6:43. doi:10.1186/1748-5908-6-43.
115. Young T, Rohwer A, Volmink J, Clarke M. What Are the Effects of Teaching Evidence-Based Health Care (EBHC)? Overview of Systematic Reviews. PLoS One. 2014;9(1):e86706. doi:10.1371/journal.pone.0086706.
116. Harris C, Turner T, Wilkinson F. SEAchange: Guide to a pragmatic evidence-based approach to Sustainable, Effective and Appropriate change in health services. 2015. Available from: http://arrow.monash.edu.au/hdl/1959.1/1225377. Accessed: 24/10/2017
117. Greenhalgh T, Robert G, Macfarlane F, Bate P, Kyriakidou O. Diffusion of innovations in service organizations: systematic review and recommendations. The Milbank quarterly. 2004;82(4):581-629. doi:10.1111/j.0887-378X.2004.00325.x.
118. McMaster University. Evidence Alerts. DynaMed Plus and McMaster University's Health Information Research Unit https://plus.mcmaster.ca/EvidenceAlerts/. Accessed 24/10/2017.
119. BMJ. Clinical Evidence. http://clinicalevidence.bmj.com/x/index.html. Accessed 24/10/2017.
120. Centre for Evidence Based Dermatology. CEBD Evidence Updates. University of Nottingham. https://www.nottingham.ac.uk/research/groups/cebd/resources/cebd-evidence-updates.aspx. Accessed 24/10/2017.
121. Lavis JN. How can we support the use of systematic reviews in policymaking? PLoS medicine. 2009;6(11):e1000141. doi:10.1371/journal.pmed.1000141.
122. Dobbins M, Cockerill R, Barnsley J. Factors affecting the utilization of systematic reviews. A study of public health decision makers. International journal of technology assessment in health care. 2001;17(2):203-14.
123. Dobbins M, Ciliska D, Cockerill R, Barnsley J, DiCenso A. A framework for the dissemination and utilization of research for health-care policy and practice. Online J Knowl Synth Nurs. 2002;9:7.
124. Dobbins M, Jack S, Thomas H, Kothari A. Public health decision-makers' informational needs and preferences for receiving research evidence. Worldviews on evidence-based nursing / Sigma Theta Tau International, Honor Society of Nursing. 2007;4(3):156-63. doi:10.1111/j.1741-6787.2007.00089.x.
125. Armstrong R, Waters E, Crockett B, Keleher H. The nature of evidence resources and knowledge translation for health promotion practitioners. Health promotion international. 2007;22(3):254-60. doi:10.1093/heapro/dam017.
126. Dobbins M, Hanna SE, Ciliska D, Manske S, Cameron R, Mercer SL et al. A randomized controlled trial evaluating the impact of knowledge translation and exchange strategies. Implementation science : IS. 2009;4:61. doi:10.1186/1748-5908-4-61.
127. Lavis JN, Robertson D, Woodside JM, McLeod CB, Abelson J. How can research organizations more effectively transfer research knowledge to decision makers? The Milbank quarterly. 2003;81(2):221-48, 171-2.
128. Haynes RB, Holland J, Cotoi C, McKinlay RJ, Wilczynski NL, Walters LA et al. McMaster PLUS: a cluster randomized clinical trial of an intervention to accelerate clinical use of evidence-based information from digital libraries. Journal of the American Medical Informatics Association : JAMIA. 2006;13(6):593-600. doi:10.1197/jamia.M2158.
129. Dobbins M, Rosenbaum P, Plews N, Law M, Fysh A. Information transfer: what do decision makers want and need from researchers? Implementation science : IS. 2007;2:20. doi:10.1186/1748-5908-2-20.
130. Chambers D, Wilson PM, Thompson CA, Hanbury A, Farley K, Light K. Maximizing the impact of systematic reviews in health care decision making: a systematic scoping review of knowledge-translation resources. The Milbank quarterly. 2011;89(1):131-56. doi:10.1111/j.1468-0009.2011.00622.x.
131. Karkos B, Peters K. A Magnet community hospital: Fewer barriers to nursing research utilization. The Journal of nursing administration. 2006;36(7-8):377-82.
132. Brown CE, Wickline MA, Ecoff L, Glaser D. Nursing practice, knowledge, attitudes and perceived barriers to evidence-based practice at an academic medical center. Journal of advanced nursing. 2009;65(2):371-81. doi:10.1111/j.1365-2648.2008.04878.x.
133. Fronsdal KB, Facey K, Klemp M, Norderhaug IN, Morland B, Rottingen JA. Health technology assessment to optimize health technology utilization: using implementation initiatives and monitoring processes. International journal of technology assessment in health care. 2010;26(3):309-16. doi:10.1017/s0266462310000309.
134. Greenhalgh T, Humphrey C, Hughes J, Macfarlane F, Butler C, Pawson R. How do you modernize a health service? A realist evaluation of whole-scale transformation in london. The Milbank quarterly. 2009;87(2):391-416. doi:10.1111/j.1468-0009.2009.00562.x.
135. Robinson S, Dickinson H, Williams I, Freeman T, Rumbold B, Spence K. Setting priorities in health: A study of English primary care trusts: Health Services Management Centre, University of Birmingham and the Nuffield Trust, 2011.
136. Peacock SJ, Mitton C, Ruta D, Donaldson C, Bate A, Hedden L. Priority setting in healthcare: towards guidelines for the program budgeting and marginal analysis framework. Expert review of pharmacoeconomics & outcomes research. 2010;10(5):539-52. doi:10.1586/erp.10.66.
137. Joshi NP, Stahnisch FW, Noseworthy TW. Reassessment of Health Technologies: Obsolescence and Waste. 2009.
138. O'Leary D F, Mhaolrunaigh SN. Information-seeking behaviour of nurses: where is information sought and what processes are followed? Journal of advanced nursing. 2012;68(2):379-90. doi:10.1111/j.1365-2648.2011.05750.x.

139. Centre for Clinical Effectiveness. Sustainability in Health care by Allocating Resources Effectively (SHARE): Evaluation and Research Plan. Southern Health 2009. Available from: https://figshare.com/articles/Sustainability_in_Healthcare_by_Allocating_Resources_Effectively_SHARE_Evaluation_and_Research_Plan/3979575. Accessed: 24/10/2017
140. Harris C, Brooke V, Turner T, Wilkinson F. Implementation of evidence-based paediatric guidelines: evaluation of complex interventions based on a theoretical framework. Centre for Clinical Effectiveness 2007. Available from: http://arrow.monash.edu.au/hdl/1959.1/1218931. Accessed: 24/10/2017
141. Harris C, Green S, Ramsey W, Allen K, King R. Sustainability in Health care by Allocating Resources Effectively (SHARE) 1: Introducing a series of papers reporting an investigation of disinvestment in a local healthcare setting BMC health services research. 2017. doi:10.1186/s12913-017-2210-7.
142. Meyer J. Evaluating action research. Age Ageing. 2000;29 Suppl 2:8-10.
143. Meyer J. Qualitative research in health care. Using qualitative methods in health related action research. BMJ. 2000;320(7228):178-81.
144. Harris C, Allen K, Waller C, Voutier C, Brooke V. Health technology disinvestment: tests, drugs and clinical practice. Report on a national disinvestment workshop. Part 1: Report. Centre for Clinical Effectiveness 2009. Available from: http://arrow.monash.edu.au/hdl/1959.1/1218926. Accessed: 17/04/2017
145. Harris C, Allen K, Waller C, Voutier C, Brooke V. Health technology disinvestment: tests, drugs and clinical practice. Report on a national disinvestment workshop. Part 2: Appendices. Centre for Clinical Effectiveness 2009. Available from: http://arrow.monash.edu.au/hdl/1959.1/1218922. Accessed: 17/04/2017
146. Harris C, Green S, Ramsey W, Allen K, King R. Sustainability in Health care by Allocating Resources Effectively (SHARE) 9: Conceptualising disinvestment in the local healthcare setting BMC health services research. 2017. doi:10.1186/s12913-017-2388-8.
147. Department of Human Services. Future directions for health technology uptake, diffusion and disinvestment in Victorian public health services. Department of Human Services, Victoria, Australia. 2007.
148. Adelaide Health Technology Assessment. https://www.adelaide.edu.au/ahta/services/hta/. Accessed 10 August 2017.
149. Barasa EW, Molyneux S, English M, Cleary S. Setting Healthcare Priorities at the Macro and Meso Levels: A Framework for Evaluation. International journal of health policy and management. 2015;4(11):719-32. doi:10.15171/ijhpm.2015.167.
150. Bhatia RS, Levinson W, Shortt S, Pendrith C, Fric-Shamji E, Kallewaard M et al. Measuring the effect of Choosing Wisely: an integrated framework to assess campaign impact on low-value care. BMJ quality & safety. 2015;24(8):523-31. doi:10.1136/bmjqs-2015-004070.
151. Smith N, Mitton C, Peacock S, Cornelissen E, MacLeod S. Identifying research priorities for health care priority setting: a collaborative effort between managers and researchers. BMC health services research. 2009;9:165. doi:10.1186/1472-6963-9-165.
152. Mortimer D. Reorienting programme budgeting and marginal analysis (PBMA) towards disinvestment. BMC health services research. 2010;10:288. doi:10.1186/1472-6963-10-288.
153. Robinson S, Glasby J, Allen K. 'It ain't what you do it's the way that you do it': lessons for health care from decommissioning of older people's services. Health & social care in the community. 2013;21(6):614-22. doi:10.1111/hsc.12046.
154. Eddama O, Coast J. A systematic review of the use of economic evaluation in local decision-making. Health policy (Amsterdam, Netherlands). 2008;86(2-3):129-41. doi:10.1016/j.healthpol.2007.11.010.
155. Mayer J, Nachtnebel A. Disinvesting from ineffective technologies: Lessons learned from current programs. International journal of technology assessment in health care. 2015:1-8. doi:10.1017/s0266462315000641.
156. Decamp M, Farber NJ, Torke AM, George M, Berger Z, Keirns CC et al. Ethical Challenges for Accountable Care Organizations: A Structured Review. Journal of general internal medicine. 2014. doi:10.1007/s11606-014-2833-x.
157. Rawlins MD. Evidence, values, and decision making. International journal of technology assessment in health care. 2014:1-6. doi:10.1017/s0266462314000154.
158. Indiana University Center for Bioethics. Ethics Guide for Health Care Practitioners. Working Under Conditions of an Influenza Pandemic. 2009. Available from: https://scholarworks.iupui.edu/bitstream/handle/1805/2011/EthicsGuide-Pandemic_Pocket.pdf?sequence=1&isAllowed=y. Accessed: September 2015
159. Lindstrom H, Waldau S. Ethically acceptable prioritisation of childless couples and treatment rationing: "accountability for reasonableness". European journal of obstetrics, gynecology, and reproductive biology. 2008;139(2):176-86. doi:10.1016/j.ejogrb.2008.02.018.
160. World Health Organisation. Guidance on ethics and equitable access to HIV treatment and care. 2004. Available from: http://www.who.int/hiv/pub/advocacy/en/guidanceethics_en.pdf?ua=1. Accessed: September 2015
161. Transplantation Society of Australia and New Zealand. Organ transplantation from deceased donors: Consensus statement on eligibility criteria and allocation protocols. 2014. Available from: http://www.donatelife.gov.au/sites/default/files/files/Concensus_Statement_v3.pdf. Accessed: September 2015
162. NHS Commissioning Board. Commissioning Policy: Ethical framework for priority setting and resource allocation 2013. Available from: http://www.england.nhs.uk/wp-content/uploads/2013/04/cp-01.pdf. Accessed: 29/09/2017

163. Stafinski T, Menon D, McCabe C, Philippon DJ. To fund or not to fund: development of a decision-making framework for the coverage of new health technologies. Pharmacoeconomics. 2011;29(9):771-80. doi:10.2165/11539840-000000000-00000.
164. Singer PA, Martin DK, Giacomini M, Purdy L. Priority setting for new technologies in medicine: qualitative case study. BMJ. 2000;321(7272):1316-8.
165. World Health Organisation. Guiding principles on human cell, tissue and organ transplantation. 2008. Available from: http://www.who.int/transplantation/Guiding_PrinciplesTransplantation_WHA63.22en.pdf. Accessed: September 2015
166. Panteli D, Kreis J, Busse R. Considering equity in health technology assessment: An exploratory analysis of agency practices. International journal of technology assessment in health care. 2015;31(5):314-23. doi:10.1017/s0266462315000549.
167. Organisation for Economic Co-operation and Development. The OECD Health Project: Health Technologies and Decision Making. OECD Publishing 2005. Available from: http://www.keepeek.com/Digital-Asset-Management/oecd/science-and-technology/health-technologies-and-decision-making_9789264016224-en#page1. Accessed: 13/06/2017
168. Culyer A. Efficiency, equity and equality in health and health care. CHE Research Paper 120. Centre for Health Economics, University of York, UK, 2015.
169. Levesque JF, Harris MF, Russell G. Patient-centred access to health care: conceptualising access at the interface of health systems and populations. International journal for equity in health. 2013;12:18. doi:10.1186/1475-9276-12-18.
170. Wolfson D, Santa J, Slass L. Engaging physicians and consumers in conversations about treatment overuse and waste: a short history of the choosing wisely campaign. Academic medicine : journal of the Association of American Medical Colleges. 2014;89(7):990-5. doi:10.1097/acm.0000000000000270.
171. Sibbald SL, Singer PA, Upshur R, Martin DK. Priority setting: what constitutes success? A conceptual framework for successful priority setting. BMC health services research. 2009;9:43. doi:10.1186/1472-6963-9-43.
172. Daniels N, Sabin J. Accountability for reasonableness: an update. BMJ. 2008;337. doi:10.1136/bmj.a1850.
173. Smith N, Mitton C, Bryan S, Davidson A, Urquhart B, Gibson JL et al. Decision maker perceptions of resource allocation processes in Canadian health care organizations: a national survey. BMC health services research. 2013;13:247. doi:10.1186/1472-6963-13-247.
174. Audit Commission. Reducing spending on low clinical value treatments. Health Briefing. London, 2011
175. Leggett L, MacKean G, Noseworthy T, Sutherland L, Clement F. Current status of health technology reassessment of non-drug technologies: survey and key informant interviews. Health Research Policy and Systems 2012. doi:10.1186/1478-4505-10-38.
176. NHS Confederation. Two sides of the same coin. Balancing quality and finance to deliver greater value. 2014. Available from: http://www.nhsconfed.org/~/media/Confederation/Files/Publications/Documents/two_sides_same_coin150413.pdf. Accessed: 05/05/2017
177. Wiltsey Stirman S, Kimberly J, Cook N, Calloway A, Castro F, Charns M. The sustainability of new programs and innovations: a review of the empirical literature and recommendations for future research. Implementation science : IS. 2012;7:17. doi:10.1186/1748-5908-7-17.
178. Armstrong R, Waters E, Dobbins M, Anderson L, Moore L, Petticrew M et al. Knowledge translation strategies to improve the use of evidence in public health decision making in local government: intervention design and implementation plan. Implementation science : IS. 2013;8(1):121. doi:10.1186/1748-5908-8-121.
179. Massatti RR, Sweeney HA, Panzano PC, Roth D. The de-adoption of innovative mental health practices (IMHP): why organizations choose not to sustain an IMHP. Administration and policy in mental health. 2008;35(1-2):50-65. doi:10.1007/s10488-007-0141-z.
180. Poulin P, Austen L, Scott CM, Poulin M, Gall N, Seidel J et al. Introduction of new technologies and decision making processes: a framework to adapt a Local Health Technology Decision Support Program for other local settings. Medical devices (Auckland, NZ). 2013;6:185-93. doi:10.2147/mder.s51384.
181. Garcia-Armesto S, Campillo-Artero C, Bernal-Delgado E. Disinvestment in the age of cost-cutting sound and fury. Tools for the Spanish National Health System. Health policy (Amsterdam, Netherlands). 2013;110(2-3):180-5. doi:10.1016/j.healthpol.2013.01.007.
182. Leggett L, Noseworthy TW, Zarrabi M, Lorenzetti D, Sutherland LR, Clement FM. Health technology reassessment of non-drug technologies: current practices. International journal of technology assessment in health care. 2012;28(3):220-7. doi:10.1017/S0266462312000438.
183. Noseworthy T, Clement F. Health technology reassessment: Scope, methodology, & language. International journal of technology assessment in health care. 2012;28(03):201-2. doi:doi:10.1017/S0266462312000359.
184. Ruano-Ravina A, Velasco-Gonzalez M, Varela-Lema L, Cerda-Mota T, Ibargoyen-Roteta N, Gutierrez-Ibarluzea I et al. Identification, prioritisation and assessment of obsolete health technologies. A methodolgical guideline. HTA Reports: avalia-t No. 2007/01: Galician Health Technology Assessment Agency, 2009.
185. Iglesias CP, Drummond MF, Rovira J. Health-care decision-making processes in Latin America: problems and prospects for the use of economic evaluation. International journal of technology assessment in health care. 2005;21(1):1-14.
186. Segal L, Mortimer D. A population-based model for priority setting across the care continuum and across modalities. Cost Effectiveness & Resource Allocation. 2006;4:6.

187. Bryan S, Mitton C, Donaldson C. Breaking the addiction to technology adoption. Health Economics. 2014;23(4):379-83. doi:10.1002/hec.3034.
188. Boivin A, Lehoux P, Lacombe R, Burgers J, Grol R. Involving patients in setting priorities for healthcare improvement: a cluster randomized trial. Implementation science : IS. 2014;9:24. doi:10.1186/1748-5908-9-24.
189. Blumenthal-Barby JS. “Choosing Wisely” to Reduce Low-Value Care: A Conceptual and Ethical Analysis. Journal of Medicine and Philosophy. 2013;38(5):559-80. doi:10.1093/jmp/jht042.
190. Liverani M, Hawkins B, Parkhurst JO. Political and institutional influences on the use of evidence in public health policy. A systematic review. PLoS One. 2013;8(10):e77404. doi:10.1371/journal.pone.0077404.
191. Jewell CJ, Bero LA. "Developing good taste in evidence": facilitators of and hindrances to evidence-informed health policymaking in state government. The Milbank quarterly. 2008;86(2):177-208. doi:10.1111/j.1468-0009.2008.00519.x.
192. Garner S, Docherty M, Somner J, Sharma T, Choudhury M, Clarke M et al. Reducing ineffective practice: challenges in identifying low-value health care using Cochrane systematic reviews. Journal of health services research & policy. 2013;18(1):6-12. doi:10.1258/jhsrp.2012.012044.
193. Vogel JP, Oxman AD, Glenton C, Rosenbaum S, Lewin S, Gulmezoglu AM et al. Policymakers' and other stakeholders' perceptions of key considerations for health system decisions and the presentation of evidence to inform those considerations: an international survey. Health research policy and systems / BioMed Central. 2013;11:19. doi:10.1186/1478-4505-11-19.
194. Bastian H, Scheibler F, Knelangen M, Zschorlich B, Nasser M, Waltering A. Choosing health technology assessment and systematic review topics: the development of priority-setting criteria for patients' and consumers' interests. International journal of technology assessment in health care. 2011;27(4):348-56. doi:10.1017/s0266462311000547.
195. Larmour I, Pignataro S, Barned KL, Mantas S, Korman MG. A therapeutic equivalence program: evidence-based promotion of more efficient use of medicines. The Medical journal of Australia. 2011;194(12):631-4.
196. Oxman AD, Lewin S, Lavis JN, Fretheim A. SUPPORT Tools for evidence-informed health Policymaking (STP) 15: Engaging the public in evidence-informed policymaking. Health research policy and systems / BioMed Central. 2009;7 Suppl 1:S15. doi:10.1186/1478-4505-7-s1-s15.
197. Harris C, Allen K, King R, Ramsey W, Green S. Sustainability in Health care by Allocating Resources Effectively (SHARE) 11: Reporting outcomes of an evidence-driven approach to disinvestment in a local healthcare setting. BMC health services research. 2017;in press.
